# Just Soaping Them: The Simplest Method for Converting Metal Organic Frameworks into Superhydrophobic Materials

**DOI:** 10.1021/acsami.3c19536

**Published:** 2024-02-29

**Authors:** Dimitrios A. Evangelou, Anastasia D. Pournara, Vasiliki I. Karagianni, Christos Dimitriou, Evangelos K. Andreou, Yiannis Deligiannakis, Gerasimos S. Armatas, Manolis J. Manos

**Affiliations:** † Department of Chemistry, University of Ioannina, Ioannina GR-45110, Greece; ‡ Department of Physics, University of Ioannina, Ioannina GR-45110, Greece; § Department of Materials Science and Technology, University of Crete, Heraklion GR-70013, Greece

**Keywords:** MOFs, post-synthetic modification, superhydrophobic materials, porous materials, oil-in-water separation

## Abstract

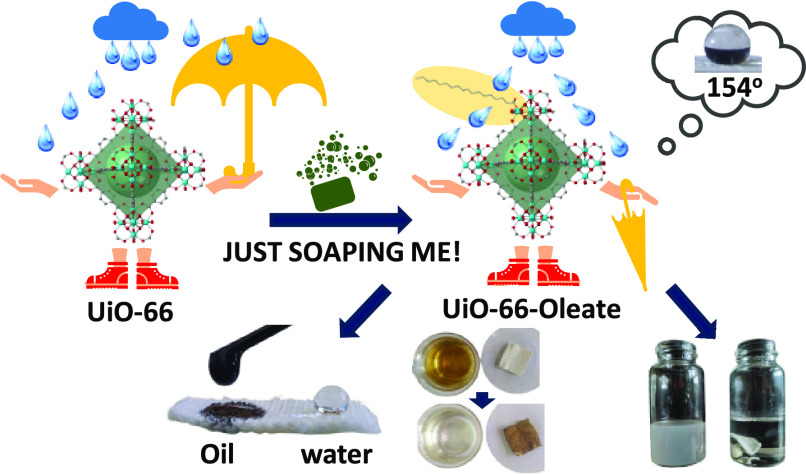

The incorporation of superhydrophobic properties into metal organic framework (MOF) materials is highly desirable to enhance their hydrolytic stability, gas capture selectivity in the presence of humidity and efficiency in oil–water separations, among others. The existing strategies for inducing superhydrophobicity into MOFs have several weaknesses, such as increased cost, utilization of toxic reagents and solvents, applicability for limited MOFs, etc. Here, we report the simplest, most eco-friendly, and cost-effective process to impart superhydrophobicity to MOFs, involving a rapid (90 min) treatment of MOF materials with solutions of sodium oleate, a main component of soap. The method can be applied to both hydrolytically stable and unstable MOFs, with the porosity of modified MOFs approaching, in most cases, that of the pristine materials. Interestingly, this approach was used to isolate superhydrophobic magnetic MOF composites, and one of these materials formed stable liquid marbles, whose motion could be easily guided using an external magnetic field. We also successfully fabricated superhydrophobic MOF-coated cotton fabric and fiber composites. These composites exhibited exceptional oil sorption properties achieving rapid removal of floating crude oil from water, as well as efficient purification of oil-in-water emulsions. They are also regenerable and reusable for multiple sorption processes. Overall, the results described here pave the way for an unprecedented expansion of the family of MOF-based superhydrophobic materials, as virtually any MOF could be converted into a superhydrophobic compound by applying the new synthetic approach.

## Introduction

Hydrophobic and superhydrophobic materials, i.e., materials with a water contact angles greater than 90 and 150°, respectively, are of interest for a variety of applications, including oil–water separations, moisture-repellent coatings, waterproof clothing, etc.^1−6^ Incorporating porosity into such materials broadens the range of their applications, enabling the removal of trace lipophilic contaminants from water or air, carbon dioxide capture under humid conditions, and selective recognition of hydrophobic molecules, among other functions.^7−9^

Metal–organic frameworks (MOFs) represent a class of porous materials that have gained substantial attention over the past two decades due to their remarkable properties and utility in various fields such as gas storage, ion exchange, catalysis, and so on.^10−20^

Most of these materials are hydrophilic and therefore either hydrolytically unstable or their properties are significantly altered in the presence of moisture. To date, several strategies have been employed to render MOFs hydrophobic, with the two most common approaches involving: (a) the utilization of polytopic organic ligands with fluorinated, polyaromatic, or long-chain alkyl groups, and (b) postsynthetic modification (PSM).^21−25^

PSM methods appear to be more attractive for applications as they primarily modify the outer surface of the MOF particles rather than the internal structure, thus largely preserving the porosity of the original materials.^26−28^ However, several limitations are associated with reported PSM approaches such as high cost, the use of toxic reagents, and applicability to only a limited number of MOFs, among others.^22,29−35^ Moreover, for applications related to the sorption/removal of lipophilic contaminants from aqueous media, the hydrophobic/superhydrophobic MOFs should be prepared in a form that enables easy recovery after use.^36,37^ Thus, research efforts should also be focused on this direction.

Oleic acid, in its sodium salt form (CH_3_(CH_2_)_7_CH=CH(CH_2_)_7_COONa), is a primary component of soap, functioning as an emulsifying agent with significant surface activity and solubility in aqueous media. Sodium oleate is particularly cost-effective since it can be readily prepared by neutralizing free oleic acid or saponifying triglycerides.^38^ In addition, it exhibits low toxicity and no carcinogenic effects.^39,40^ Oleate anion is an excellent ligand for metal ions, coordinating to them through its carboxylate group,^41,42^ and has been widely used as capping agent for metal nanoparticles.^43−45^ Considering all above features of sodium oleate, it appears to be an ideal reagent for modifying the wetting properties of MOFs through a straightforward and affordable route involving coordination of metal centers with the powerful oleate ligand. It is anticipated that sodium oleate could effectively transform most MOFs into hydrophobic/superhydrophobic materials, regardless of the type of metal ion, ligand, or metal coordination environment. This modification may proceed through the binding of oleate anions to the external surface of MOF particles, replacing the surface-terminated hydroxyl/water ligands.^46,47^

Here, we demonstrate the facile and rapid transformation of several MOFs, including Zr_6_O_4_(OH)_4_(BDC)_6_ (**UiO-66**, with BDC^2–^=terephthalate anion),^48^ Zr_6_O_4_(OH)_4_(NH_3_^+^–BDC)_6_Cl_6_ (**MOR-1**),^49^ Zn(mIm)_2_ (**ZIF-8**, with mIm=2-methylimidazole),^50^ Cu_3_(BTC)_2_(H_2_O)_3_ (**HKUST-1**, with BTC^3–^=trimesate),^51^ and Al(OH)(BDC) (**MIL-53(Al)**)^52^ into superhydrophobic materials, simply by treating them with solutions of the inexpensive and harmless sodium oleate (purity ≥ 82%). Simultaneously, these materials, except for modified **MIL-53(Al)**, retain a significant percentage (70–100%) of the internal porosity of the original (untreated) MOFs. By applying this newly developed method, we also managed to isolate superhydrophobic MOF-Fe_3_O_4_ composites. Interestingly, one of these materials was able to form robust magnetic liquid marbles, whose motion could be controlled with an external magnet. Additionally, we successfully prepared superhydrophobic MOF-coated cotton fabrics and fibers either via *in situ* modification of MOFs immobilized on the substrate or postsynthetic immobilization of the superhydrophobic material onto the substrate with the aid of poly(methyl methacrylate) (PMMA). These composite materials can quickly sorb floating oil from aqueous media and efficiently purify oil-in-water emulsions. At the same time, these fabric/fiber-based sorbents are also regenerable and reusable for multiple cycles and can be easily recovered after the separation process by simply pulling them out.

## Results and Discussion

### Post-Synthetic Modification of UiO-66

**Figure 1:**
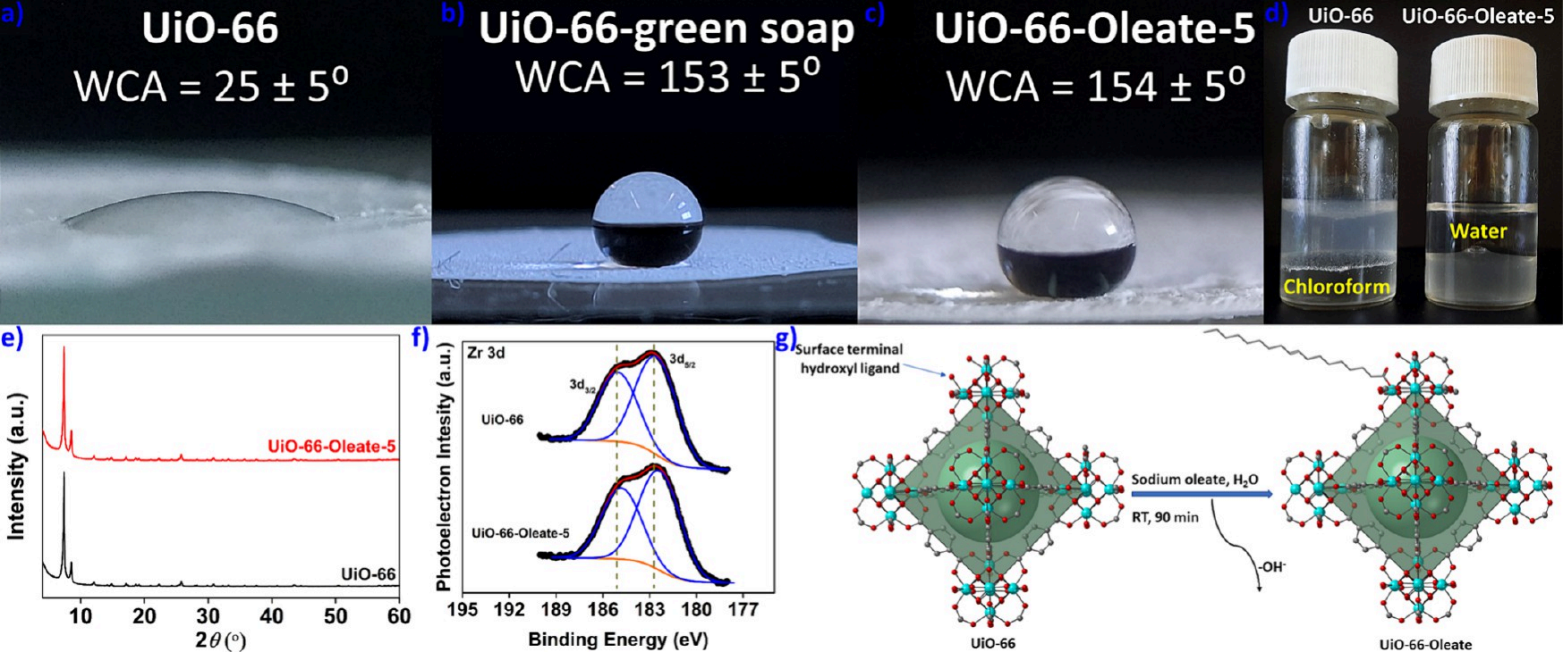
Digital images of water droplets on thin films of (a) **UiO-66**, (b) **UiO-66-green soap**, and (c) **UiO-66-Oleate-5** along with the determined WCA values. (d) Digital image of **UiO-66** and **UiO-66-Oleate-5** dispersing in water/chloroform mixtures. (e) Comparative PXRD data for **UiO-66** and **UiO-66-Oleate-5**. (f) High resolution Zr 3d core-level photoelectron spectra of **UiO-66** and **UiO-66-Oleate-5**. The peaks assigned to Zr 3d_3/2_ and 3d_5/2_ appear at 182.7/185.1 and 182.4/184.8 eV, respectively. (g) Suggested mechanism for the interaction of oleate anions with the **UiO-66** framework.

Our studies on the conversion of MOFs into superhydrophobic materials initiated with water stable MOFs, specifically focusing on **UiO-66**, one of the most extensively studied MOFs.^6,13,16,17,20,21,36,37,48,49^ At an early stage, **UiO-66** was treated with an aqueous solution of sodium oleate with a concentration of 18.5 mM for 90 min at room temperature. Subsequently, the MOF was isolated by filtration and underwent rinsing with water and then acetone to remove all weakly bound oleate anions. The resultant material, namely, **UiO-66-Oleate-1**, floated on the water surface (Figure S1), which was the initial evidence of its hydrophobicity. In contrast, **UiO-66** formed a fine colloidal suspension in water. Contact angle measurements of **UiO-66-Oleate-1** demonstrated its superhydrophobic nature, revealing a water contact angle (WCA) of 167 ± 5° (Figure S2), whereas the WCA of the untreated **UiO-66** sample was 25 ± 5° (Figure 1a). PXRD measurements and Le Bail refinement revealed that the **UiO-66-Oleate-1** retains the structural features of the original MOF (Figure S3).

Considering that oleate is a main component of soap, we attempted to convert **UiO-66** into a superhydrophobic material by treating it with an aqueous solution of green soap. To our delight, such treatment resulted in imparting superhydrophobicity to **UiO-66**, exactly like its treatment with the oleate solution, as revealed by WCA measurements (Figure 1b). The above finding emphasizes the simplicity and effectiveness of the new method.

To find out whether the inserted oleate molecules affect the internal surface area of the MOF, nitrogen gas physisorption studies were performed for the activated **UiO-66-Oleate-1**. The calculated Brunauer–Emmett–Teller (BET) surface area for **UiO-66-Oleate-1** was determined to be 336 m^2^/g, indicating a significant void reduction compared to pristine **UiO-66** (1041 m^2^/g) (Figure S4). These findings prompted us to investigate whether we could be able to generate superhydrophobic materials while maintaining the inherent porosity of **UiO-66**. Thus, we altered our experimental procedure by modifying the sodium oleate concentrations (i.e., 12.3, 6.2, 4.3, 2.5, and 1.8 mM and the materials denoted as **UiO-66-Oleate-2** up to **UiO-66-Oleate-6**, respectively). The new MOFs displayed superhydrophobic properties (WCAs > 151 ± 5°), except for **UiO-66-Oleate-6**, which turned out to be hydrophilic with a WCA of 58 ± 5° (Figure S2). Therefore, the optimum concentration to impart superhydrophobicity to **UiO-66** was found to be 2.5 mM. The completely different wetting properties of **UiO-66** and **UiO-66-Oleate-5** are illustrated in Figure 1a,c,d. Nitrogen gas physisorption data for the activated **UiO-66-Oleate-5** showed that a significant percentage of the original porosity is retained (BET surface area of 725 m^2^/g) proving that the oleate molecules are predominantly located on the outer surface of the MOF particles (Figure S5). The BET surface area for the activated **UiO-66-Oleate-3** (683 m^2^/g) turned out to be somewhat lower compared to **UiO-66-Oleate-5** (Figure S6).

The nonlocal density functional theory (NLDFT) pore size distribution analysis showed that both **UiO-66** and **UiO-66-Oleate-5** materials have pore sizes of ≈5.6 and ≈9 Å (Figures S7 and S8), consistent with those reported in the literature.^53,54^ The absence of changes in pore widths observed after treating the MOF with sodium oleate further supports the incorporation of oleate anions on the external surface rather than within the internal structure of the material. This restriction on the entry of oleate into the pores of the MOF is presumably due to the significantly larger size of oleate anions (≈2 nm)^42^ (Figure S9) compared to the pore widths of **UiO-66**. The reduction in BET surface areas of **UiO-66** samples upon treatment with increased oleate concentration likely aligns with the formation of a dense oleate layer at the surface of the MOF, preventing gas molecules from diffusing into the pores.

**UiO-66-Oleate-5** and **UiO-66-Oleate-3** could additionally be isolated following the same experimental procedure by utilizing an ethanolic solution of sodium oleate. The solvent modification does not appear to significantly affect the porosity and the wetting properties of the materials (see Figures S6 and S10).

Considering that **UiO-66-Oleate-5** accomplishes both significant porosity and superhydrophobicity, it was extensively characterized via a variety of techniques. PXRD data and Le Bail refinement revealed that **UiO-66-Oleate-5** maintains the structural features of the pristine material (Figure 1e and Figure S11). To further investigate the composition, ^1^H NMR data were obtained after digesting **UiO-66-Oleate-5** in a highly alkaline (2 M NaOH) D_2_O solution. This process resulted in the decomposition of the framework into insoluble Zr(IV) species and soluble terephthalate/oleate anions. The results revealed the characteristic signals of oleate ions in the aliphatic region of the ^1^H NMR spectrum (Figure S12). According to the peak integrals, the oleate to MOF molar ratio was determined to be ≈0.08. Similar results were obtained for **UiO-66-Oleate-1** (Figure S13). FT-IR data in the spectrum of **UiO-66-Oleate-5** revealed the presence of characteristic bands of oleate at 2926 and 2854 cm^–1^, which correspond to the asymmetric and symmetric stretch of −CH_2_–, respectively (Figure S14). Field emission-scanning electron microscopy (FE-SEM) images illustrated that the morphology of both **UiO-66** and **UiO-66-Oleate-5** particles was similar (Figure S15).

In the next step, we decided to investigate the influence of pH on the superhydrophobic properties of **UiO-66-Oleate-5**, as well as its wettability in different types of aqueous media. The experiments were carried out by depositing liquid droplets of extremely alkaline/acidic aqueous solutions alongside samples of sea, lake, and tap water. The measured WCAs demonstrated that **UiO-66-Oleate-5** remains highly hydrophobic in a wide pH range from 0 to 13, and similar results were observed in various aqueous media (Figure S16). In addition, it is worth mentioning that the material displays a WCA of 150 ± 5° even after drying at 150 °C for 12 h (Figure S16). Additionally, PXRD studies revealed that the modified **UiO-66** MOF retains its structural characteristics after treatment with highly acidic/alkaline solutions and the various aqueous media (Figures S17 and S18).

The above results suggest a strong binding of oleate ions to the MOF. Thermogravimetric (TGA) analysis (see section Calculation of the linker deficiencies in **UiO-66 MOF**, SI) revealed linker deficiencies (Figure S19) in the as prepared **UiO-66** MOF, with the missing linkers replaced by water and hydroxyl terminal ligands. Considering the strong affinity of Zr^4+^ for carboxylate ligands, oleate anions could easily substitute the terminal OH^–^/H_2_O ligands on the Zr^4+^ centers. X-ray photoelectron spectroscopy (XPS) (Figure 1f and Figure S20) data were used to confirm the possible ligation of oleate ions to the Zr^4+^ anions. Specifically, the XPS spectrum of **UiO-66-Oleate-5** revealed a remarkable negative shift (about −0.3 eV) in the Zr 3d_5/2_ and 3d_3/2_ core-level signals (Figure 1f), in comparison to the corresponding signals in the pristine **UiO-66**. These results suggest an increase of the electron density around Zr^4+^ nodes after oleate modification, providing direct proof of the coordination of oleate anions to the Zr^4+^ centers.^55,56^ Additionally, zeta potential measurements showed that **UiO-66** has neutral surface charge, thus excluding probable electrostatic interactions of the MOF’s surface with the oleate anions (Figure S21). Energy dispersive spectroscopy (EDS) and XPS data indicated the absence of Na^+^ in the modified material (Figures S22 and S20), which implies that the insertion of oleate anions likely results in the release of surface, terminal hydroxyl ligands to achieve charge neutrality, as depicted in Figure 1g.

### Post Synthetic Modification of Other Hydrolytically Stable MOFs

The described synthetic method is also applicable to other Zr^4+^ MOFs, such as **MOR-1**.^49^ Treating **MOR-1** with an aqueous oleate solution of 0.8 mM resulted in a superhydrophobic material with a WCA of 155 ± 5°, denoted as **MOR-1-Oleate** (Figure S23). Oleate concentrations below that value generated hydrophilic materials. The activated **MOR-1-Oleate** shows a BET surface area (846 m^2^/g), approaching that (1114 m^2^/g) of original material (Figure S24). PXRD data and Le Bail refinement indicated that the modified ammonium analogue of **UiO-66** retained the structural characteristics of the pristine material (Figure S25). FT-IR studies also demonstrated the existence of the characteristic IR bands of oleate at 2926 and 2854 cm^–1^ (Figure S26). It was also important to investigate whether our approach could be successful in imparting superhydrophobicity to other types of MOFs. For these studies, we selected **ZIF-8**(50) and **MIL-53(Al)**(52) as representative water-stable MOFs. Using a similar synthetic protocol to that applied for Zr^4+^ MOFs, we isolated the superhydrophobic versions of **ZIF-8** and **MIL-53(Al)**. The minimum sodium oleate concentrations required to impart superhydrophobicity to these MOFs were found to be 4.5 and 9.8 mM, respectively. The new materials denoted as **ZIF-8-Oleate** and **MIL-53-Oleate** exhibited superhydrophobic behavior with WCAs of 154 ± 5 and 153 ± 5°, respectively (Figure S23). Despite the fact that **ZIF-8** and **MIL-53** contain metal ions with no terminal ligands, which could be promptly exchanged by oleate anions, they are easily transformed to superhydrophobic materials through their treatment with sodium oleate solutions. This can be explained by the presence of surface-terminated hydroxyl/water groups in these materials (and presumably most MOFs), which can be readily replaced with oleate ligands.^46,47^ The determined BET surface area for **ZIF-8-Oleate** (1156 m^2^/g) was found to be identical, within the error limit, to that (1144 m^2^/g) of pristine material (Figure S27). However, the BET surface area of **MIL-53-Oleate** (411 m^2^/g) was significantly decreased compared to that (986 m^2^/g) of the original MOF (Figure S28). PXRD measurements and Le Bail refinement indicated that the modified MOFs retained the structural features of the pristine materials (Figures S29 and S30). In addition, FT-IR studies confirmed the presence of oleate ions in each of the modified MOF materials (Figures S31 and S32).

### Post Synthetic Modification of Hydrolytically Unstable MOFs

**Figure 2:**
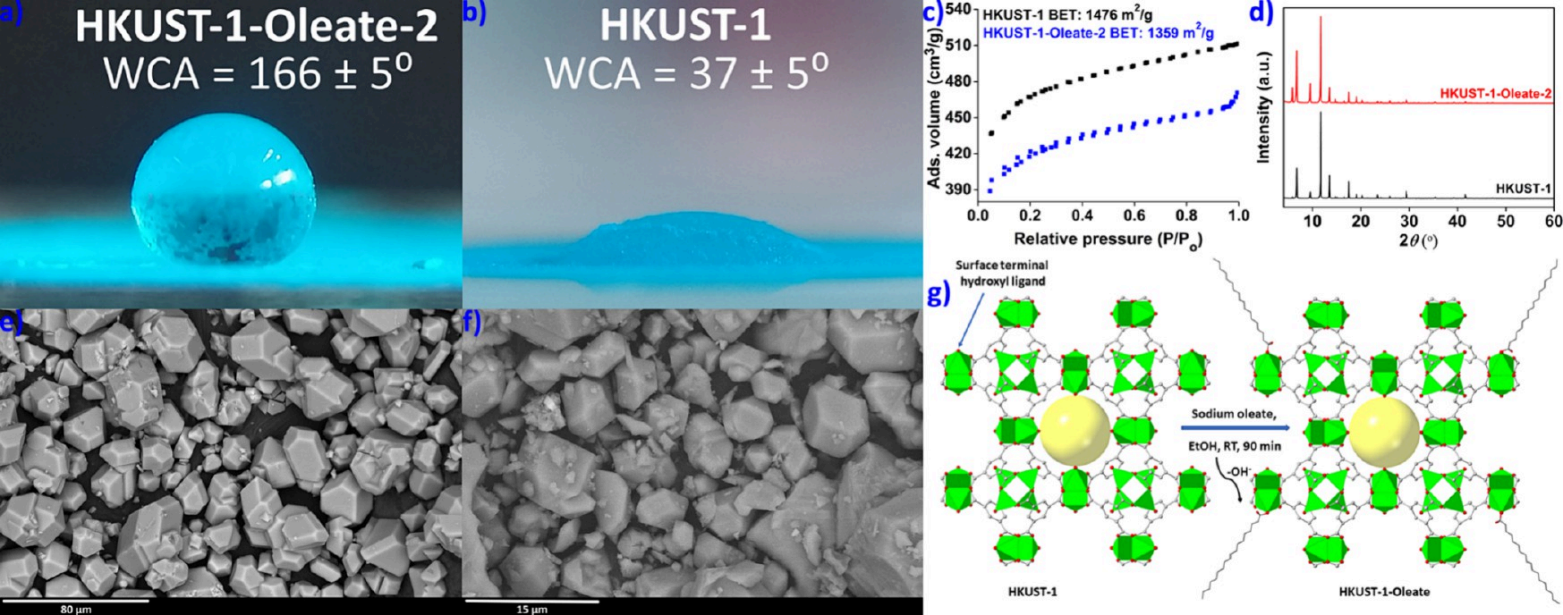
Digital images of water droplets on thin films of (a) **HKUST-1-Oleate-2** and (b) **HKUST-1**, along with the determined WCA values. (c) Nitrogen physisorption isotherms at 77 K for **HKUST-1** and **HKUST-1-Oleate-2**. (d) Comparative PXRD data for **HKUST-1** and **HKUST-1-Oleate-2**. (e) FE-SEM image of **HKUST-1**. (f) FE-SEM image of **HKUST-1-Oleate-2**. (g) Suggested mechanism for the interaction of oleate ions with the **HKUST-1** framework.

It was challenging to test our method for modification of MOFs that are hydrolytically unstable, especially with MOFs like **HKUST-1**, which undergoes phase transformation upon exposure to water.^57^ In this case, an aqueous solution of sodium oleate cannot be used because it leads to immediate degradation of the MOF’s structure. Therefore, we chose ethanol, which is considered a green solvent,^58^ instead of water to impart superhydrophobicity to **HKUST-1**. Initially, we proceeded as in the case of **UiO-66**, by experimenting with different oleate concentrations (i.e., 46, 31, and 15 mM), while investigating the wettability and surface area of the modified materials denoted as **HKUST-1-Oleate-1**, **HKUST-1-Oleate-2**, and **HKUST-1-Oleate-3**. WCA data showed that **HKUST-1-Oleate-1** and **HKUST-1-Oleate-2** are superhydrophobic with WCA values of 167 ± 5 and 166 ± 5°, respectively, demonstrating the effectiveness of our method even for hydrolytically unstable MOFs (Figure S33 and Figure 2a). However, the BET surface areas for these materials varied significantly. **HKUST-1-Oleate-2** displayed a BET surface area of 1359 m^2^/g, which is close to that of pristine **HKUST-1** being 1476 m^2^/g, confirming that the oleate molecules occupy the outer surface of the **HKUST-1** particles (Figure 2c). On the contrary, **HKUST-1-Oleate-1** exhibits significantly lower BET surface area (839 m^2^/g, Figure S34). Regarding **HKUST-1-Oleate-3**, although it fully retains the porosity of the original material (Figure S35), the observed water contact angle was determined at 47 ± 5° (Figure S33). Sodium oleate concentrations lower than 31 mM led to WCA values of <90°, probably due to the insufficient abundance of surface anchored oleate groups.

Pore size distribution studies indicated no change in the pore sizes of **HKUST-1**, which were found to be ≈5.5 and ≈9.1 Å (Figures S36 and S37), in accordance with literature values,^59,60^ upon treatment with oleate anions. Similar to **UiO-66**, the decrease of the BET surface areas of **HKUST-1** samples when treated with concentrated oleate solutions can be attributed to the formation of a dense oleate layer at the MOF’s surface, considering that oleate anions are too large to penetrate the pores of **HKUST-1**.

As **HKUST-Oleate-2** is both superhydrophobic and highly porous (largely retaining the porosity of the pristine material), it was characterized in more detail. PXRD measurements and Le Bail refinement for **HKUST-1-Oleate-2** revealed no structural alternations compared with the pristine material (Figure 2d and Figure S38). FT-IR data further confirmed the presence of the characteristic IR bands of oleate such as the alkyl −CH_2_– vibrational peaks at 2926 and 2854 cm^–1^ (Figure S39). In addition, ^1^H NMR spectroscopy measurements also confirmed the grafting of oleate ions (Figure S40). Based on the peak integrals, the Oleate to **HKUST-1** molar ratio was determined to be ≈0.07. FE-SEM images demonstrated that the morphology of **HKUST-1** and **HKUST-1-Oleate-2** particles was similar (Figure 2e,f).

We then investigated the wetting properties of the superhydrophobic **HKUST-1-Oleate-2** with various water samples (sea, lake, and tap water) and acidic/alkaline aqueous solutions. The calculated WCAs (Figure S41) showed that **HKUST-1-Oleate-2** remains superhydrophobic in a pH range of 3 to 10 and in different types of aqueous media. Furthermore, PXRD indicated that modified **HKUST-1** preserves its structural integrity after treatment with various aqueous solutions for 4 or even 24 h (Figures S42 and S43).

Additionally, we aimed to investigate whether the superhydrophobic **HKUST-1-Oleate-2** exhibits improved stability to humidity compared to the original framework. Both MOFs were placed in a closed, controlled humid system at room temperature for 3 days. The results revealed that the PXRD pattern of **HKUST-1** contained diffraction peaks not existing in the pattern of the pristine MOF, indicating structural alterations upon interaction with water vapors (Figure S44). On the contrary, **HKUST-1-Oleate-2** retained its structural features as it was revealed by Le Bail refinement (Figure S45). In conjunction with PXRD data, N_2_ physisorption studies further confirmed the structural differentiations observed in **HKUST-1** by showing a remarkable decrease in its BET surface area down to 178 m^2^/g (Figure S46), while the oleate modified material retained a substantial portion of its porosity with a BET surface area of 978 m^2^/g (Figure S47). The above clearly indicates that the post synthetic modification significantly enhances the stability of **HKUST-1** in a humid environment.

Finally, similarly to the investigation conducted for the **UiO-66** MOF, we explored the mechanism of oleate modification in the case of **HKUST-1**. Based on N_2_ physisorption and pore size distribution studies for **HKUST-1-Oleate-2**, the interaction between oleate ions and **HKUST-1** takes place predominantly in the external surface resulting in the preservation of large void space. XPS data for **HKUST-1-Oleate-2** (Figures S48 and S49) revealed a negative shift, up to −0.2 eV, in the Cu 2p_3/2_ and 2p_1/2_ core-level signals, in comparison to the corresponding signals for the pristine **HKUST-1** material. This is consistent with an increase in the electron density around the Cu^2+^ metal ions, implying coordination and thus charge transfer interactions between the oleate anions and Cu^2+^ centers.^55,56^ The EDS spectrum and XPS data of **HKUST-1-Oleate-2** (Figures S50 and S48) revealed no Na^+^ residues from sodium oleate. Taking into consideration the above results, we suggest that the ligation of oleate anions to Cu^2+^ centers coincides with the release of terminal OH^–^ groups from the external surface of **HKUST-1** particles (Figure 2g).

### Comparing Our Method with Other PSM Approaches

At this point, it is useful to compare the new method with known approaches and modifications to generate superhydrophobicity in MOFs. As shown in Table S1, our method represents (a) one of the fastest, being completed in less than 2 h, while other methods require reaction times of at least 12 h, and (b) one of the very few that does not utilize organic solvents, except for the transformation of hydrolytically unstable MOFs requiring the use of ethanol. In contrast to several known PSM methods that are effective only in MOFs bearing linkers with specific functional groups,^6,22,23,27^ this method is applicable to MOFs with a variety of metal ions and linkers. Moreover, in our approach, superhydrophobicity is conferred by ligation of oleate ions, an inexpensive, widely available, and low-toxicity organic substance.^38−45^ The estimated cost for the conversion of **UiO-66** into a superhydrophobic material, considering the retail price of crude sodium oleate (purity ≥82%, cost ≈58 €/kg) and the negligible solvent and energy costs (as the process takes place in aqueous solutions and ambient temperature for only 90 min), is calculated approximately 4 € per kg of MOF. In contrast, other methods involve the use of costly and/or toxic materials such as perfluorinated agents, phosphate-containing surfactants, polymer coatings etc., or require specialized equipment and synthetic conditions.^22,23,27,30−32,35^ Therefore, the above comparison demonstrates that the method described here is widely applicable and probably the “greenest” and least expensive method ever described for the conversion of MOFs into superhydrophobic materials. Importantly, this method also results in the isolation of superhydrophobic materials with surface areas comparable to those of the pristine MOFs. Notably, modified **HKUST-1** and **ZIF-8** retained up to 92 and 100% of the original materials’ porosity, respectively. Such remarkable porosity retention for superhydrophobic **HKUST-1** and **ZIF-8** has been reported in the literature.^31,35^ However, the synthetic approaches toward these modified MOFs entail perfluorinated or other hazardous reagents and toxic solvents.

### Formation of Magnetic Liquid Marbles

**Figure 3:**
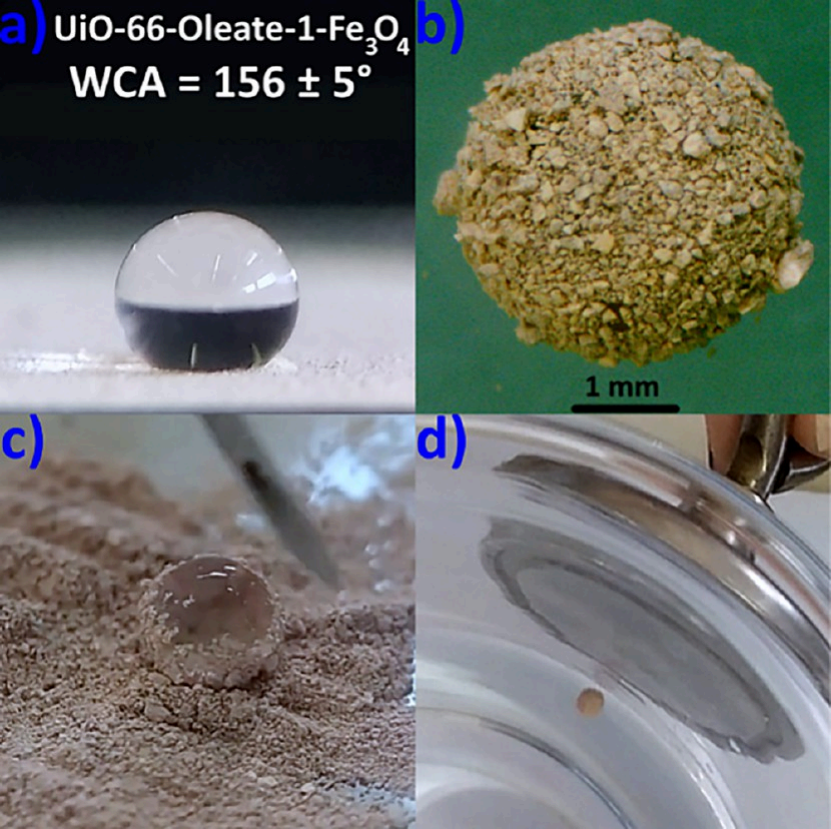
(a) Digital image of a water droplet on a thin film of **UiO-66-Oleate-1-Fe**_**3**_**O**_**4**_ with the indication of the determined WCA value. (b) Digital stereoscopic image of a liquid marble based on the **UiO-66-Oleate-1-Fe**_**3**_**O**_**4**_ material. (c) A water droplet rolling over **UiO-66-Oleate-1-Fe**_**3**_**O**_**4**_ particles. (d) Controlling the motion of the liquid marble using an external magnet.

Superhydrophobic/hydrophobic particles enclosing liquid droplets enable the isolation of nonstick droplets, known as liquid marbles. These hold potential applications in a variety of different fields, for example gas sensing, miniaturized synthesis and micro reactors, cultivation of microorganisms, etc.^61,62^ The development of liquid marbles with strong magnetic properties has significant interest, as the motion of magnetic liquid marbles can be easily controlled using an external magnetic field, allowing them to act as mobile phases to transfer miscellaneous types of liquids to specific locations on various surfaces.^63^ This feature of magnetic liquid marbles is attractive for microfluidic applications.^64^ To isolate a magnetic MOF-based liquid marble, we aimed to prepare superhydrophobic, magnetic MOF composites, utilizing the synthetic route employed for the isolation of the superhydrophobic MOF materials. Specifically, a mixture of **UiO-66** and Fe_3_O_4_ (mass ratio **UiO-66**:Fe_3_O_4_ = 100:10) was treated with an 18.5 mM aqueous oleate solution at room temperature. Remarkably, the resulting composite, i.e., **UiO-66-Oleate-1-Fe**_**3**_**O**_**4**_, displayed a WCA of 156 ± 5° (Figure 3a).

PXRD data once again confirmed that this new material retained the structural features of the pristine **UiO-66** MOF (Figures S51 and S52). N_2_ physisorption measurements for the activated **UiO-66-Oleate-1-Fe**_**3**_**O**_**4**_ showed a surface area of 338 m^2^/g (Figure S53). FT-IR and ^1^H NMR spectroscopy also confirmed the presence of oleate ions (Figures S54 and S55). FE-SEM images revealed that the particles of the magnetic composite had morphology resembling that of the **UiO-66** MOF (Figure S56). EDS data confirmed the presence of Fe in the magnetic composites (Figure S57). Notably, **UiO-66-Oleate-1-Fe**_**3**_**O**_**4**_ effectively stabilized water droplets by forming concrete liquid marbles (Figure 3b). The droplets could easily roll over a pile of magnetic composite while picking up MOF particles until the droplets were entirely covered (Figure 3c and Video S1). The resulting liquid marbles displayed excellent mechanical robustness and their motion can readily be controlled with an external magnetitic field (Figure 3d and Video S2). We should note that additional superhydrophobic MOF-Fe_3_O_4_ composites could be isolated, such as **UiO-66-Oleate-2-Fe**_**3**_**O**_**4**_ and **HKUST-1-Oleate-1-Fe**_**3**_**O**_**4**_ (their detailed characterization is provided in SI, Figures S58–S70). However, these composites could not form liquid marbles as stable as those made by **UiO-66-Oleate-1-Fe**_**3**_**O**_**4**_.

The fact that **UiO-66-Oleate-2-Fe**_**3**_**O**_**4**_ is not superhydrophobic, showing WCA= 141 ± 5° (Figure S58), in contrast to **UiO-66-Oleate-1-Fe**_**3**_**O**_**4**_ (Figures 3a), likely justifies the limited capability of the former material to create stable liquid marbles. Regarding **HKUST-1-Oleate-1-Fe**_**3**_**O**_**4**_**,** despite exhibiting superhydrophobicity with WCA = 164 ± 5° (Figure S58), it forms liquid marbles with lower mechanical robustness compared to those of **UiO-66-Oleate-1-Fe**_**3**_**O**_**4**_. The likely explanation for this aligns with the significantly larger sizes (up to 37 μm) of **HKUST-1-Oleate-1-Fe**_**3**_**O**_**4**_ particles (Figure S68) compared to those (up to 0.2 μm) of **UiO-66-Oleate-1-Fe**_**3**_**O**_**4**_ (Figure S56), which may affect the enclosing properties of the materials.

### Immobilization of the Superhydrophobic MOFs onto Substrates

**Figure 4:**
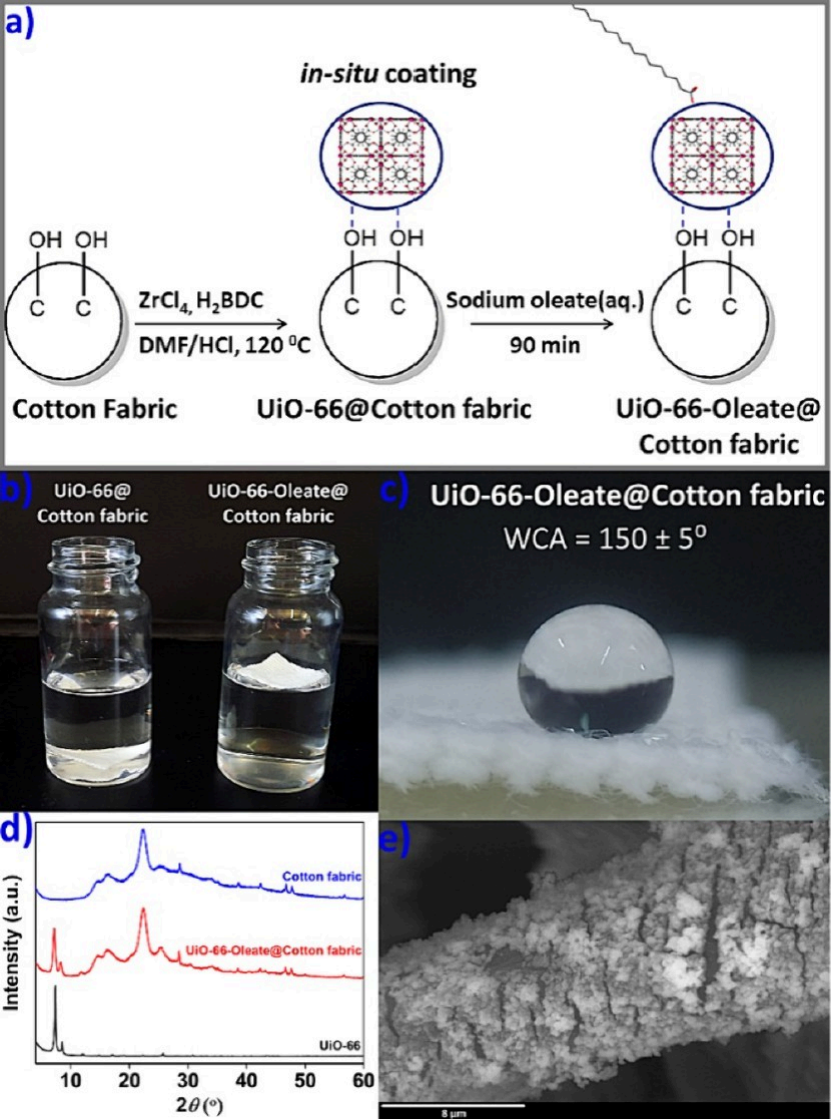
(a) Two-step *in situ* coating route for the isolation of **UiO-66-Oleate@Cotton fabric**, (b) Digital image of **UiO-66-Oleate@Cotton fabric** floating on the water surface in comparison to **UiO-66@Cotton fabric**. (c) Digital image of a water droplet on **UiO-66-Oleate@Cotton fabric,** along with the determined WCA value. (d) Comparative PXRD data of **UiO-66**, **UiO-66-Oleate@Cotton fabric**, and cotton fabric. (e) FE-SEM image of **UiO-66-Oleate@Cotton fabric**.

We also desired to investigate the possibility of incorporating the superhydrophobic MOFs into stable substrates. Initially, we successfully immobilized **UiO-66-Oleate** into a cotton fabric and impart superhydrophobicity through a two-step procedure involving *in situ* growth of **UiO-66** on the cotton fabric and then treatment of **UiO-66@Cotton fabric** with an aqueous sodium oleate solution at ambient temperature (Figure 4a). The resulting **UiO-66-Oleate@Cotton fabric** floated on the water surface, in contrast to unmodified **UiO-66@Cotton fabric**, which easily submerged (Figure 4b). The WCA for the composite was determined to be 150 ± 5° (Figure 4c). PXRD and FTIR data confirmed the presence of **UiO-66-Oleate** onto the cotton fabric (Figure 4d and Figure S71). Moreover, FE-SEM and EDS studies revealed the presence of MOF particles on the textile fibers (Figure 4e and Figure S72).

**Figure 5:**
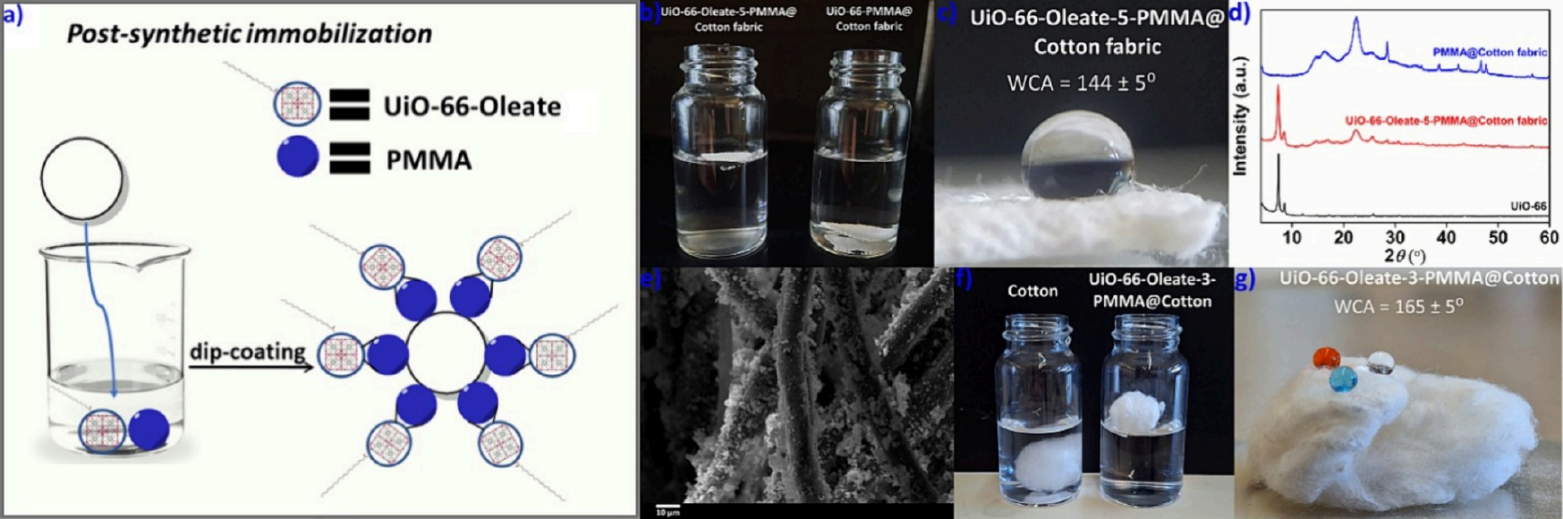
(a) Postsynthetic immobilization of **UiO-66-Oleate-PMMA** mixture onto the cotton fabric/fiber. (b) Digital image of **UiO-66-Oleate-5-PMMA@Cotton fabric** floating on the water surface in comparison to **UiO-66-PMMA@Cotton fabric**. (c) Digital image of a water droplet on **UiO-66-Oleate-5-PMMA@Cotton fabric**, along with the determined WCA value. (d) Comparative PXRD data of **UiO-66**, **UiO-66-Oleate-5-PMMA@Cotton fabric**, and **PMMA@Cotton fabric**. (e) FE-SEM image of **UiO-66-Oleate-5-PMMA@Cotton fabric**. (f) Digital image of **UiO-66-Oleate-3-PMMA@Cotton** floating on the water surface in comparison to cotton substrate. (g) Digital image of liquid droplets (dye solutions and water) on **UiO-66-Oleate-3-PMMA@Cotton**, along with the determined WCA value.

As an alternative method, we employed a postsynthetic approach for the immobilization of the superhydrophobic MOF onto the cotton fabric. Thus, we effectively incorporated the superhydrophobic **UiO-66-Oleate-5** onto the cotton fabric using the inexpensive and nontoxic poly(methyl methacrylate) (PMMA) as an adhesive agent. **UiO-66-Oleate-5** was suspended in a PMMA solution in acetone (mass ratio MOF:PMMA = 10:1) via ultrasonication. A piece of cotton fabric was submerged into the stirred coating solution for a few seconds. After a dipping and drying procedure at room temperature, a dense **UiO-66-Oleate-5-PMMA** coating was obtained on the cotton fabric (Figure 5a). The MOF displayed strong adhesion, as the quantity of MOF immobilized onto cotton fabrics remained identical after water treatment of the fabric composites for 24 h (Experimental Section). **UiO-66-Oleate-5-PMMA@Cotton fabric** floats on the water surface unlike **UiO-66-PMMA@Cotton fabric** (Figure 5b). The water contact angle for **UiO-66-Oleate-5-PMMA@Cotton fabric** was estimated to be 144 ± 5° (Figure 5c). PXRD and FTIR studies also revealed the effective incorporation of **UiO-66-Oleate-5** onto the cotton substrate (Figure 5d and Figure S73). FE-SEM images and EDS data revealed the coating of the fabric fibers by MOF particles (Figure 5e and Figure S74).

By employing the same method, we were able to prepare a cotton fiber-based composite denoted as **UiO-66-Oleate-3-PMMA@Cotton**. The modified cotton fiber displayed superhydrophobicity in contrast to the original substrate and the WCA was calculated to be 165 ± 5° (Figure 5f,g). FE-SEM, EDS, and PXRD data confirmed the successful immobilization of **UiO-66-Oleate-3** onto the cotton fiber (Figures S75–S77).

Subsequently, we decided to apply the same protocols to **HKUST-1**. Unfortunately, the two-step procedure was not efficient, as the MOF particles were released from the fabric during the hydrophobic modification. We also explored different surface immobilization methods, such as enriching the substrate with pendant carboxyl or catechol groups, but we encountered the same difficulty.^65,66^

**Figure 6:**
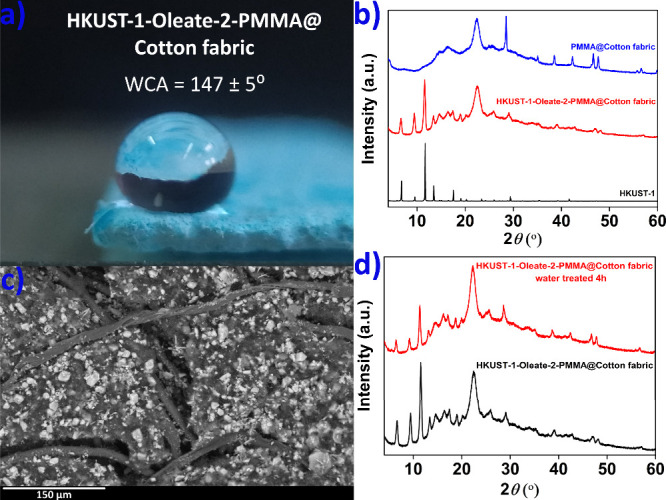
(a) Digital image of a water droplet on **HKUST-1-Oleate-2-PMMA@Cotton fabric**, along with the determined WCA value. (b) Comparative PXRD data of **HKUST-1**, **HKUST-1-Oleate-2-PMMA@Cotton fabric**, and PMMA@Cotton fabric. (c) FE-SEM image of **HKUST-1-Oleate-2-PMMA@Cotton fabric**. (d) Comparative PXRD data of **HKUST-1-Oleate-2-PMMA@ Cotton fabric** before and after water treatment.

Hence, we focused on the MOF-PMMA approach by applying the same experimental procedure described above for **UiO-66-Oleate-5**. However, efficient adhesion of **HKUST-1-Oleate-2** onto the cotton fabrics was achieved using a larger amount of PMMA (MOF to PMMA mass ratio was 1:1). The new composite (denoted as **HKUST-1-Oleate-2-PMMA@ Cotton fabric**) displayed high hydrophobicity with a WCA of 147 ± 5° (Figure 6a). The efficient immobilization of **HKUST-1-Oleate-2** was confirmed by PXRD, FTIR and FE-SEM/EDS measurements (Figure 6b,c and Figures S78 and S79). Importantly, the immobilized **HKUST-1-Oleate-2** material retained its structural characteristics upon interaction with water (Figure 6d).

### Oil/Water Separation

In the past decades, an enormous volume of oily wastewater has been regularly discharged into the ecosystem from various oil-based activities such as petroleum refining, metalworking and machining, food processing, chemicals production, textile manufacturing, etc. Furthermore, frequent oil leakage accidents during transportation and refilling incidents pose considerable environmental issues. Oily wastewater may contain various toxic substances, which severely affect the marine habitat and the seafood resulting to the accumulation of hazardous chemicals into the human body.^2,36,37,67,68^ Therefore, there is a crucial demand to develop novel, cost-effective and environmentally friendly oil/water separation materials.^69^

**Figure 7:**
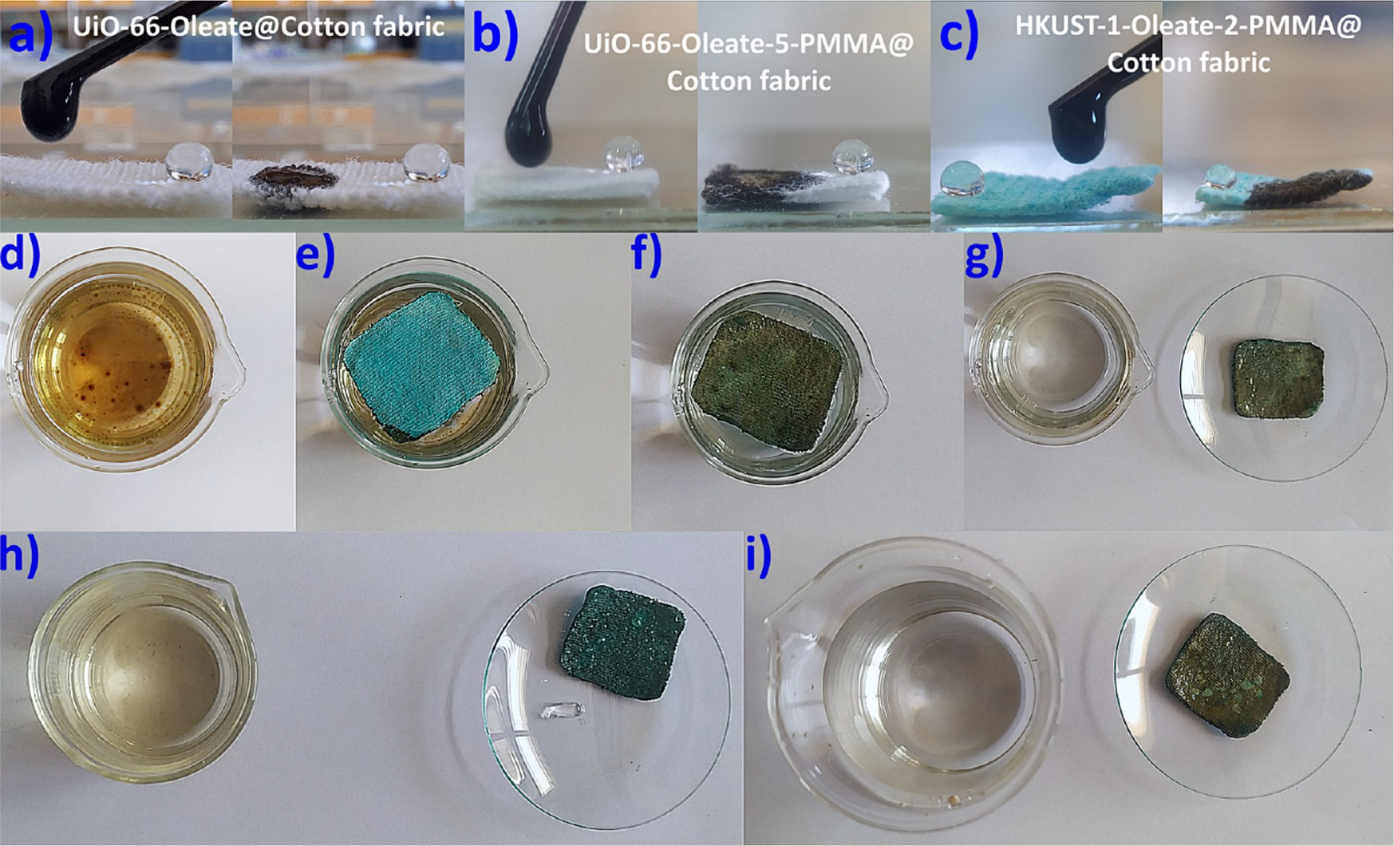
Digital images demonstrating the oleophilicity and hydrophobicity of (a) **UiO-66-Oleate@Cotton fabric**, (b) **UiO-66-Oleate-5-PMMA@Cotton fabric**, and (c) **HKUST-1-Oleate-2-PMMA@Cotton fabric**. (d–g) Crude oil sorption from the water surface by **HKUST-1-Oleate-2-PMMA@Cotton fabric**. (h) Desorbed oil from the oil-laden fabric after its treatment with *n*-hexane and the regenerated **HKUST-1-Oleate-2-PMMA@Cotton fabric**. (i) Oil-free water solution and the **HKUST-1-Oleate-2-PMMA@Cotton fabric** after the 10th cycle of sorption/regeneration.

Aiming toward practical oil–water separation applications, the lipophilicity of the superhydrophobic cotton fabric composites (**UiO-66-Oleate@Cotton fabric**, **UiO-66-Oleate-5-PMMA@Cotton fabric**, and **HKUST-1-Oleate-2-PMMA@Cotton fabric**) was also tested. As shown in Figure 7a–c, the MOF@fabric composites can easily sorb crude oil droplets. Hence, they were investigated for the removal of crude oil floating in water. It was found that all three different fabrics successfully sorbed the oil from the water surface (Figures S80 and S81, Figure 7d–g, and Video S3). On the contrary, unmodified cotton fabric is highly hydrophilic. Thus, it is easily submerged upon contact with water (Figure 4b) and its sorption capacity for crude oil under static conditions is negligible. The oil-laden fabrics could be regenerated with *n*-hexane, and the sorption/regeneration procedure was repeated up to 10 times (Figures S80 and S81 , Figure 7h,i, and Video S3). PXRD data revealed no structural differentiations of the MOF-oleate materials after 10 cycles of sorption/regeneration (Figures S82–S84). Similar crude oil removal efficiency and reusability was observed for **UiO-66-Oleate-3-PMMA@Cotton** as depicted in Figure S85.

**Figure 8:**
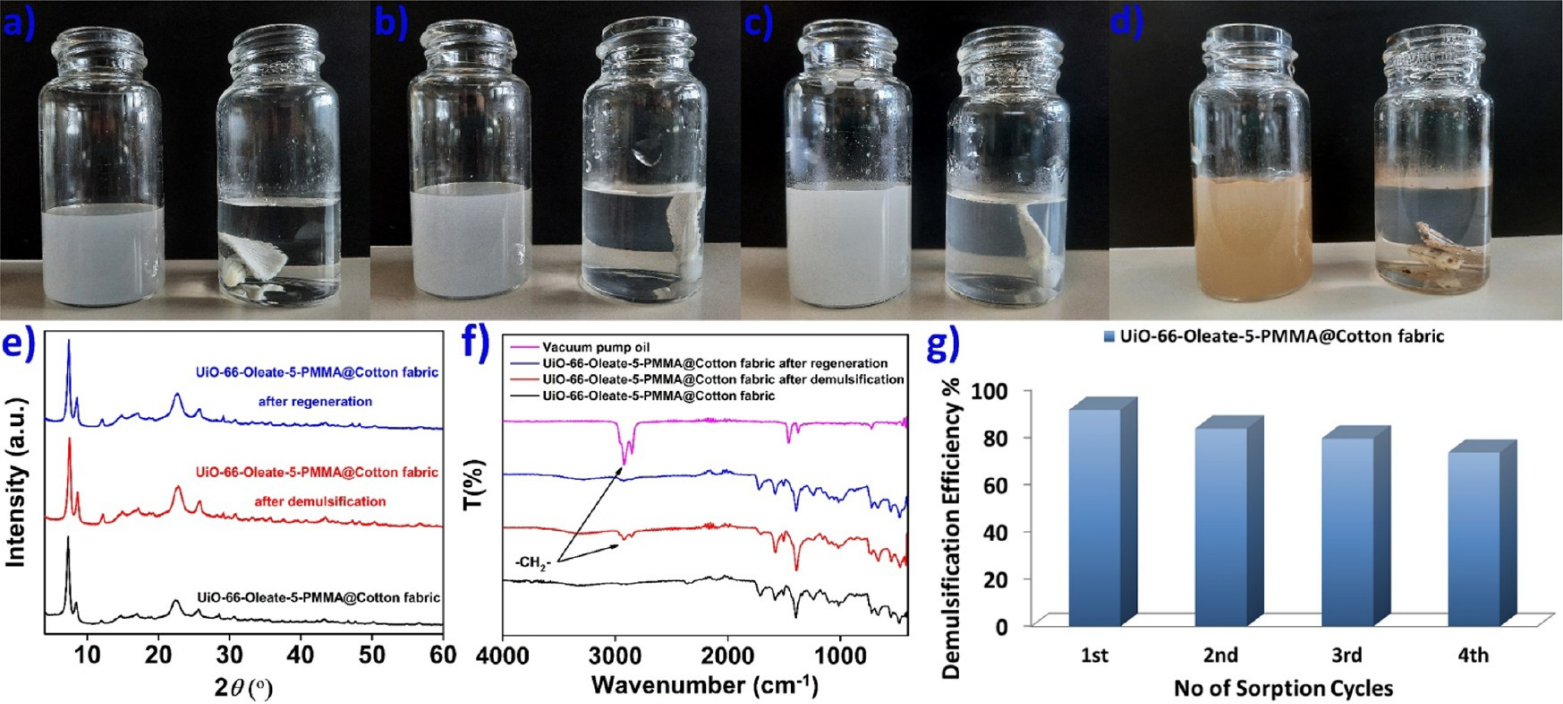
Vacuum pump oil-in-water emulsions with initial oil concentrations (a) *C* ≈ 540 ppm, (b) *C* ≈ 780 ppm, and (c) *C* ≈ 1040 ppm before and after a 20 h treatment with **UiO-66-Oleate-5-PMMA@Cotton fabric**. (d) Crude oil-in-water emulsion with initial oil concentration *C* ≈ 1005 ppm before and after a 20 h treatment with **UiO-66-Oleate-5-PMMA@Cotton fabric**. (e) Comparative PXRD data of **UiO-66-Oleate-5-PMMA@Cotton fabric** before and after demulsification of vacuum pump oil-in-water emulsion (with initial oil concentration of ≈1040 ppm) as well as after regeneration with *n*-hexane. (f) Comparative FTIR spectra of a vacuum pump oil sample, **UiO-66-Oleate-5-PMMA@Cotton fabric** before and after demulsification of vacuum pump oil-in-water emulsion (with initial oil concentration of ≈1040 ppm) as well as after regeneration with *n*-hexane. (g) Demulsification performance, evaluated on vacuum pump oil-in-water emulsion with initial oil concentration of ≈1040 ppm, of **UiO-66-Oleate-5-PMMA@Cotton fabric** during four cycles of sorption/regeneration.

Furthermore, we tested the capability of the MOF@cotton composites for treatment of oil-in-water emulsions. Specifically, we have investigated the efficiency of the **UiO-66-Oleate-5-PMMA@Cotton fabric** for purification of vacuum pump oil-in-water emulsions (initial oil concentration ≈540 ppm). The fabric was fixed onto a magnetic stirring bar, by applying ordinary adhesive, to remain immersed during the demulsification process. The experiment was conducted under mild stirring, at ambient conditions, for 20 h and the purification efficiency was determined via UV–vis spectroscopy (Experimental Section). As shown in Figure 8a, **UiO-66-Oleate-5-PMMA@Cotton fabric** sorbed most of the oil, resulting in a transparent mixture (removal efficiency ≈89.6%). As a further step, we decided to investigate more thoroughly the separation kinetics as well as the performance of the composite in more concentrated oil-in-water mixtures. The sorption data revealed that the equilibrium is reached at 5 h with a separation efficiency of 87.2%, while only a minor increase of 2.4% was achieved after 20 h of contact time (Figure S86). On top of that, **UiO-66-Oleate-5-PMMA@Cotton fabric** maintained its great separation performance even in emulsions of increased concentrations (Figure 8b,c and Figure S87) with the highest sorption capacity determined at 1.53 g of vacuum pump oil/g of **UiO-66-Oleate-5** (Figure S88). **UiO-66-Oleate-5-PMMA@Cotton fabric** also demonstrated high efficiency for the purification of crude oil-in-water emulsion (Figure 8d and Figure S87). Additionally, we tested the unmodified cotton for demulsification of a vacuum pump oil emulsion. Based on the variance in absorbance intensities at 238 nm (Figure S89), the unmodified fabric demonstrated demulsification efficiency of only 14.3%. The fabric composites after demulsification were studied via PXRD and FT-IR spectroscopy. PXRD data indicated no structural alterations, while FT-IR measurements confirmed the sorption of vacuum pump oil, presenting a strong band at ≈2900 cm^–1^ that is assigned to the −CH_2_– vibrations of the hydrocarbons (Figure 8e,f). We also investigated the potential reusability of the demulsifier. FT-IR data confirmed the desorption of most sorbed pump oil (Figure 8e,f) by treating the oil-loaded fabric with *n*-hexane. Importantly, the fabric sorbent could be reused up to four cycles of sorption/regeneration with no significant loss of separation performance (Figure 8g). The reused fabric was further characterized via FTIR and PXRD data (Figures S90 and S91), indicating no structural modifications. In addition, no leaching of MOF particles from the fabric was observed, as the quantity of the MOF immobilized remained unaltered after the emulsion purification processes.

## Conclusions

A new synthetic strategy for the conversion of a series of hydrophilic, water-stable MOFs into superhydrophobic materials is reported, in which oleate anions are strongly bound to the outer surface of MOF particles via the formation of metal ion-oleate coordination bonds. This method is particularly cost-effective and environmentally friendly as it does not require organic solvents or perfluorinated agents, proceeds at room temperature, and is completed within 1 to 2 h. Interestingly, even a treatment of MOFs with an aqueous solution of green soap, whose main component is sodium oleate, led to the isolation of superhydrophobic MOF materials. This method can also be effective in converting hydrolytically unstable MOFs into superhydrophobic materials, but an ethanolic solution of the fatty acid anion must be used. Remarkably, the superhydrophobic MOFs produced through the new method preserve, in most cases, a considerable percentage of the internal porosity of the pristine materials. The same approach was extended to the isolation of superhydrophobic MOF-Fe_3_O_4_ magnetic composites. One of the isolated composites could form robust magnetic liquid marbles with their motion easily manipulated by an external magnetic field. Such property is of interest for applications in microfluidics. Furthermore, we have succeeded in preparing superhydrophobic MOFs immobilized in cotton fabric or fiber. These composite fabric-based materials can remove crude oil from the water surface and effectively decontaminate oil-in-water emulsions. Simultaneously, they can be easily retrieved after use and are also easily regenerated and reused multiple times.

Overall, the present work provides a facile and quite simple synthetic approach that could potentially be applied for converting any known MOF or newly synthesized MOF compounds to superhydrophobic materials. Therefore, the family of superhydrophobic MOFs can be significantly expanded to include compounds with a variety of structural features, pore sizes, and functional groups. The resulting materials are anticipated to exhibit unprecedented properties arising from the combination of the intrinsic MOF characteristics with superhydrophobicity.

## Experimental Section

### Materials

Zirconium chloride (ZrCl_4_), zinc nitrate hexahydrate (Zn(NO_3_)_2_·6H_2_O), aluminum nitrate nonahydrate (Al(NO_3_)_3_·9H_2_O), 2-aminoterephthalic acid (NH_2_–H_2_BDC), terephthalic acid (H_2_BDC), 2-methylimidazole (C_4_H_6_N_2_), and poly(methyl methacrylate) ((C_5_H_8_O_2_)_*n*_) were purchased from Alfa Aesar. Iron Chloride Hexahydrate (FeCl_3_·6H_2_O), copper nitrate trihydrate (Cu(NO_3_)_2_·3H_2_O), sodium sulfite (Na_2_SO_3_), trimesic acid (H_3_BTC), methylene blue hydrate (C_16_H_18_ClN_3_S·*x*H_2_O), methyl orange 85% (C_14_H_14_N_3_NaO_3_S), and sodium oleate (>82%) were purchased from Aldrich. The solvents were used as received. Green soap, cotton fabric, and cotton fiber were obtained from commercial sources.

### MOF Syntheses

The pristine MOFs, i.e., **UiO-66**, **MOR-1**, **MIL-53(Al)**, **ZIF-8**, and **HKUST-1** were synthesized following experimental procedures similar to those reported previously in the literature with some minor modifications.^70−74^

### Post Synthetic Modification of Water Stable MOFs with Sodium Oleate

#### Aqueous-Based Preparation of **UiO-66-Oleate-1**

A total of 100 mg of **UiO-66** was added to an 8 mL aqueous solution of sodium oleate with a concentration of 18.5 mM. The resulting suspension was stirred at ambient temperature for 90 min. The modified **UiO-66** was isolated via centrifugation, washed with water and acetone, and dried at 80 °C. The rest of the modified **UiO-66** samples (i.e., **UiO-66-Oleate-2** up to **UiO-66-Oleate-6**) were prepared following the same experimental procedure by adjusting the concentration of the sodium oleate solution (i.e., 12.3, 6.2, 4.3, 2.5,and 1.8 mM). Yield: 75–102 mg (depending on the sodium oleate concentration).

#### Preparation of **UiO-66-Oleate-5** and **UiO-66-Oleate-3** in Ethanol

These materials were isolated through the procedure described above via the utilization of an ethanolic instead of an aqueous sodium oleate solution. Yield: 80 mg.

#### Preparation of **MOR-1-Oleate**

A total of 100 mg of **MOR-1** was added to an 8 mL aqueous solution of sodium oleate with a concentration of 0.8 mM. The resulting suspension was stirred at ambient temperature for 90 min. The modified MOF was isolated via centrifugation, washed with water and acetone, and dried at 80 °C. Yield: 80 mg.

#### Preparation of **MIL-53(Al)-Oleate**

A total of 100 mg of **MIL-53(Al)** was added to an 8 mL aqueous solution of sodium oleate with a concentration of 9.8 mM. The resulting suspension was stirred at ambient temperature for 90 min. The modified MOF was isolated via centrifugation, washed with water and acetone, and dried at 80 °C. Yield: 75 mg.

#### Preparation of **ZIF-8-Oleate**

A total of 100 mg of **ZIF-8** was added to an 8 mL aqueous solution of sodium oleate with a concentration of 4.5 mM. The resulting suspension was stirred at ambient temperature for 90 min. The modified MOF was isolated via centrifugation, washed with water and acetone, and dried at 80 °C. Yield: 85 mg.

### Post Synthetic Modification of **HKUST-1** with Sodium Oleate

#### Preparation of **HKUST-1-Oleate-1**

A total of 100 mg of **HKUST-1** was added to an 8 mL ethanolic solution of sodium oleate with a concentration of 46 mM. The resulting suspension was stirred at ambient temperature for 90 min. The modified MOF was isolated via centrifugation, washed several times with EtOH, and dried at 80 °C. The rest of the modified **HKUST-1** samples (i.e., **HKUST-1-Oleate-2** and **HKUST-1-Oleate-3**) were prepared following the same experimental procedure by adjusting the concentration of the sodium oleate solution (i.e., 31 and 15 mM). Yield: 80–90 mg (depending on the sodium oleate concentration).

### Post Synthetic Modification of **UiO-66** with Green Soap

#### Preparation of **UiO-66-green Soap**

A total of 100 mg of **UiO-66** was added to an aqueous solution of green soap (55 mg in 8 mL). The resulting suspension was stirred at room temperature for 90 min. The modified **UiO-66** was isolated via centrifugation, washed with water and acetone, and dried at 80 °C. Yield: 99 mg.

### Synthesis of Fe_3_O_4_

A total of 61.5 mg (0.49 mmol) of Na_2_SO_3_ was dissolved in 5 mL of distilled water. The Na_2_SO_3_ solution were gradually added to a 30 mL aqueous solution of FeCl_3_·6H_2_O (810.9 mg, 3 mmol) under vigorous stirring. After the addition, the color of the solution changed from light yellow to red. Thereafter, a NH_3_ aqueous solution (1.5 M) of 15 mL is gradually added to the red solution leading to the precipitation of a black solid while the color of the solution changed back to yellow. The resulting oxide (its PXRD is shown in Figure S92) was isolated via filtration, washed several times with water, and dried in air. Yield: 240 mg.

### Isolation of the Magnetic Superhydrophobic/Hydrophobic Composites

#### Preparation of **UiO-66-Oleate-1-Fe_3_O_4_**

A total of 100 mg of **UiO-66** and 10 mg of Fe_3_O_4_ were added to an 8 mL aqueous solution of sodium oleate with a concentration of 18.5 mM. The resulting mixture was stirred at room temperature for 90 min. The magnetic composite was isolated via centrifugation, washed with water and acetone, and dried at 80 °C. **UiO-66-Oleate-2-Fe**_**3**_**O**_**4**_ was isolated following the same experimental procedure by adjusting the sodium oleate solution concentration to 2.5 mM. Yield: 80–90 mg (depending on the sodium oleate concentration).

#### Preparation of **HKUST-1-Oleate-1-Fe_3_O_4_**

A total of 100 mg of **HKUST-1** and 10 mg of Fe_3_O_4_ were added to an 8 mL ethanolic solution of sodium oleate with a concentration of 31 mM. The resulting mixture was stirred at room temperature for 90 min. The magnetic composite was isolated via centrifugation, washed several times with EtOH and dried at 80 °C. Yield: 75 mg.

### Immobilization of the Superhydrophobic MOFs onto Substrates

#### Preparation of **UiO-66-Oleate@Cotton** Fabric

**UiO-66-Oleate@Cotton fabric** was prepared in two separate steps. The first step consists of the *in situ* immobilization of **UiO-66** onto the fabric substrate via a solvothermal reaction. The second step relates to the hydrophobic modification of **UiO-66@Cotton fabric** using a sodium oleate solution at room temperature.

A typical procedure is the following:

##### First Step

One piece of circular shaped cotton fabric (diameter 1.5 cm) weighing a total of 35 mg was washed three consecutive times with 5 mL of MeOH and then dried at 80 °C for 2 h. Afterward, the cotton fabric, ZrCl_4_ (62.5 mg, 0.268 mmol), and H_2_BDC (62.3 mg, 0.375 mmol) was placed in 20 mL glass vial containing a mixture of DMF/HCl (7.5 mL/0.5 mL). The reaction mixture was ultrasonicated for 10 min and the container was then sealed and allowed to react in an oven operated at 120 °C for 24 h. The next day the mixture was cooled at room temperature and the modified fabric was primarily washed multiple times with deionized H_2_O to remove the **UiO-66** that has not been incorporated into the substrate and additionally with acetone. The final product was dried at 80 °C. The immobilization process was repeated twice to increase the amount of the MOF particles onto the substrate. The **UiO-66** powder not immobilized on the substrate was isolated and further used in the preparation of **UiO-66-Oleate-5**.

##### 2nd step

**UiO-66@Cotton fabric** was added to a 10 mL aqueous solution of sodium oleate (2.7 mM) in a 20 mL glass vial. The mixture was kept under magnetic stirring for 90 min at room temperature. Thereafter the modified fabric was primarily washed with deionized H_2_O and acetone. The final product was dried at 80 °C.

#### Preparation of UiO-66-Oleate-5-PMMA@Cotton fabric

5 mg of PMMA were dissolved in 25 mL acetone under ultrasonication for 15 min. Then 10 mg of **UiO-66-Oleate-5** were added to 5 mL of the PMMA solution, and the resulting mixture was stirred for 12 h at ambient temperature in order to get a uniform suspension. A piece of circular shaped cotton fabric (diameter 1.5 cm) weighing a total of 35 mg was immersed into the suspension for a few seconds while stirring, and then dried in air. The dipping and drying procedure was repeated numerous times until all the suspension was used up. The MOF-PMMA composite was left to dry at 80 °C overnight to achieve better adhesion of the MOF into the fabric.

#### Preparation of UiO-66-Oleate-3-PMMA@Cotton

5 mg of PMMA were dissolved in 25 mL acetone under ultrasonication for 15 min. Then 10 mg of **UiO-66-Oleate-3** were added to 5 mL of the PMMA solution, and the resulting mixture was stirred for 12 h at ambient temperature in order to get a uniform suspension. A piece of cotton fiber (≈100 mg) was immersed into the suspension for a few seconds while stirring, and then dried in air. The dipping and drying procedure was repeated numerous times until all the suspension was used up. The MOF-PMMA composite was left to dry at 80 °C overnight to achieve better adhesion of the MOF into the cotton fiber.

#### Preparation of HKUST-1-Oleate-2-PMMA@Cotton fabric

10 mg of PMMA were dissolved in 5 mL acetone under ultrasonication for 15 min. Then 10 mg of **HKUST-1-Oleate-2** were added to the PMMA solution, and the resulting mixture was stirred for 12 h at ambient temperature to get a uniform suspension. A piece of circular shaped cotton fabric (diameter 1.5 cm) weighing a total of 35 mg was immersed into the suspension for a few seconds while stirring, and then dried in air. The dipping and drying procedure was repeated numerous times until all the suspension was used up. The MOF-PMMA composite was left to dry at 80 °C overnight to achieve better adhesion of the MOF into the fabric.

#### Determination of the Zr content (mg/cm^2^) of the UiO-66-Oleate@Cotton fabric

As the MOF was chemically bound to the fabric, releasing it without causing its decomposition was not feasible. Thus, direct determination of the MOF content was not possible in this case. Only the Zr content of the MOF immobilized into the fabric could be thus determined, after decomposing the MOF to ZrO_2_. Initially we attempted to determine the Zr content of the superhydrophobic fabric via its thermal treatment at 800 °C. However, we observed that a portion of the fabric did not burn after the heating process and thus, it was not possible to determine the Zr content (based on the ZrO_2_ mass) accurately. Consequently, we decided to detach the MOF-oleate from the fabric via treatment with an alkaline solution. The superhydrophobic composite was stirred for 60 min in a 20 mL NaOH solution (2 M). Extremely alkaline conditions led to the decomposition of the MOF and formation of an insoluble Zr(IV) phase. The oleate and terephthalate ions were soluble under these conditions, while the cotton fabric remained intact. The white solid was isolated via centrifugation, washed with water and dried at 80 °C. PXRD for the solid revealed a mixture of several phases. Thus, the solid underwent an 1 h thermal treatment at 800 °C to transform it to pure ZrO_2_. Based on the final mass of the oxide, the Zr content of the fabric was determined to be 0.17 mg/cm^2^ of the fabric.

#### Determination of the MOF content into the MOF-PMMA composites

By taking advantage of the high solubility of PMMA in acetone, the release of the MOF particles from MOF-PMMA@Cotton fabric or fiber composites could be easily achieved. As a result, the MOF content of the composites could be readily determined. Thus, the composites were immersed in acetone and sonicated for several minutes. The substrate was then removed, and the mixture was centrifuged to isolate the MOF particles that were released. Acetone dissolves PMMA and as a result, MOF particles cannot remain fixed into the fabric. The released MOF solid was dried in air. In the case of **HKUST-1-Oleate-2-PMMA@Cotton fabric,** the treatment with acetone was conducted multiple times to ensure the dissolution of PMMA, which was present in a much higher (10-fold) content compared to those in **UiO-66-Oleate-5-PMMA@Cotton fabric** and **UiO-66-Oleate-3@Cotton**. The MOF content for the three different composites was determined at 6.2 mg of **UiO-66-Oleate-5** per 1.77 cm^2^ of cotton fabric, 6 mg of **UiO-66-Oleate-3** per 100 mg of cotton fiber and 6.5 mg of **HKUST-1-Oleate-2** per 1.77 cm^2^ of cotton fabric.

#### Evaluation of the Adhesion of the **UiO-66-Oleate-5-PMMA** Coating on Cotton Fabric After Water Treatment

A piece of **UiO-66-Oleate-5-PMMA@Cotton fabric** was placed in a glass vial containing 10 mL of distilled water and was kept under mild stirring for 24 h at room temperature. The weight of the fabric composite remained identical following the water treatment proving the strong adhesion of the MOF-PMMA coating.

### Preparation of Thin MOF Films for Water Contact Angle Studies

Thirty mg of the oleate modified MOF was dispersed in 1.5 mL of CH_2_Cl_2_ in a glass vial. The mixture was subjected to ultrasonication for 10 min and kept under stirring. A small portion of the resulting suspension was spread on a microscope coverslip using a Pasteur pipet and dried in air. This step was repeated several times until the slip was covered with a thin film of the MOF. In advance of water contact angle determination, the MOF films remained at room temperature for a minimum of 1 h.

### Activation of **UiO-66**, **MOR-1**, **MIL-53**, and **ZIF-8** Samples Prior Gas Physisorption Studies

A total of 100 mg of the MOF was placed in a glass vial containing 4 mL of EtOH. The mixture was then stirred at ambient temperature for 24 h. The solid was isolated via centrifugation, washed with acetone, and dried at 80 °C overnight. The solvent exchange procedure was repeated two additional times.

### Evaluation of the Structural Robustness of **UiO-66-Oleate-5** and **HKUST-1-Oleate-2** in Various Aqueous Media and pH Values

Prior to the stability studies, the materials were dispersed in CH_2_Cl_2_, centrifuged, and dried in air for 1 h. Thereafter, 15 mg of the MOF were placed in a glass vial containing 8 mL of the corresponding aqueous solution and the sample was left undisturbed for 4 to 24 h at room temperature. Upon completion of the treatment, the solid was isolated via centrifugation, washed with water and acetone, and dried in air. The structural stability of the treated samples was investigated by PXRD studies.

### Oil/Water Separation

#### Static Crude Oil Removal

Two drops of crude oil (≈25 mg) were added to 100 mL of distilled water. Thereafter a piece of superhydrophobic **UiO-66-Oleate@Cotton fabric** (dimensions 4 × 4 cm^2^) was placed on the surface on each side, for a few minutes, until the floating oil was sorbed. The oil laden fabric composite was then treated twice with a 40 mL solution of *n*-hexane and dried in air for 30 min. The regenerated material was then reused for the following sorption cycle. The same process was conducted for a total of 10 cycles. The same experimental procedure was applied to the rest of hydrophobic/superhydrophobic PMMA-cotton fabric and PMMA-cotton fiber composites. The crude oil removal efficiency of **UiO-66-Oleate-3-PMMA@Cotton** was evaluated for two sorption/regeneration cycles.

#### Preparation of oil in water emulsions

The o/w emulsions of different concentrations were prepared by mixing the oil and water samples and ultrasonicating the resulting mixture for at least 30 min. The ultrasonication process was repeated, if necessary, until the oil particles were completely suspended.

#### Evaluation of the o/w emulsion separation efficiency of UiO-66-Oleate-5-PMMA@ Cotton fabric

A typical experiment is the following: A circular shaped **UiO-66-Oleate-5-PMMA@Cotton fabric** (1.5 cm diameter) was fixed onto the magnetic stirring using ordinary adhesive. Subsequently the fabric was placed in a glass vial containing 10 mL of oil emulsion and stirred at 300 rpm, at room temperature, for a certain time interval. When the demulsification process was completed, the fabric was retrieved and the demulsification efficiency was determined. The regeneration of the composite was accomplished by treatment with *n*-hexane and the fabric was dried in air for 30 min. Finally, the regenerated **UiO-66-Oleate-5-PMMA@Cotton fabric** was fixed again onto the magnetic stirring bar prior to the next sorption/regeneration cycle. The weight of the fabric composite remained identical following the demulsification process owning to the strong adhesion of the MOF-PMMA coating.

#### Estimation of the o/w emulsion separation efficiency

The % demulsification efficiency was estimated via UV–vis spectroscopy. A typical o/w emulsion spectrum displays an absorbance band peaking at 238 nm (Figure S93). Hence, the absorbance intensity at this specific wavelength was selected and the % separation efficiency was estimated by the difference of the absorbance intensities at 238 nm for the initial emulsion and the treated sample. Similar method has been previously reported in literature.^75^

### Characterization Techniques

Powder X-ray diffraction measurements were performed on a Bruker D2-Phaser X-ray diffractometer (CuKa radiation source, wavelength = 1.54184 Å). High quality diffraction data, suitable to be used for Le Bail refinement, were obtained using a step of 0.01° and a scan rate of 1.8 s per 0.01° (overall measurement time was approximately 172 min). The Le Bail refinement was performed using TOPAS.^76^^1^H NMR spectra were measured with a Bruker 500 MHz spectrometer. ATR-IR spectra were recorded in the range of 4000–400 cm^–1^ range using an Agilent Cary 630 ATR. Thermogravimetric analyses (TGA) were performed on a STA 449C Jupiter in air atmosphere with a heating rate of 10 °C min^–1^. N_2_ physisorption isotherms were measured at 77 K on a Quantachrome Novatouch LX2 sorption analyzer. Before analysis, **UiO-66**, **MOR-1**, **MIL-53** and **ZIF-8** samples were EtOH exchanged and degassed at 150 °C under vacuum (<10^–5^ Torr) for 12 h. Regarding the **HKUST-1** materials, no solvent exchange was applied, and the degassing was performed at 120 °C under vacuum (<10^–5^ Torr) for 12 h. The specific surface areas were calculated by applying the Brumauer-Emmett-Teller (BET) method to the adsorption branch of isotherms in the 0.05–0.25 relative pressure (P/P_o_) range. CO_2_ physisorption measurements were performed on a Quantachrome NOVA 3200e sorption analyzer at 0 °C. Prior measurement, all samples were degassed (<10^–5^ Torr) at 120–150 °C for 12 h. The corresponding pore-size distribution plots were obtained by fitting the CO_2_ adsorption data of the isotherms to nonlocal density functional theory (NLDFT) model.^77^ Field Emission-Scanning Electron Microscopy (FE-SEM)/Energy Dispersive Spectroscopy (EDS) measurements were performed with a Phenom Pharos G2 Desktop FEG-SEM (Thermo Fisher Scientific) on Cr sputtered specimens (Q150T ES Plus automatic sputter coater, Quorum Technologies Ltd.), as well as with a JEOL JSM-6390LV scanning electron microscope (SEM) equipped with an Oxford INCA PentaFET-x3 energy dispersive X-ray spectroscopy (EDS) detector. XPS data were collected on a SPECS spectrometer using a Phoibos 100 1D-DLD electron analyzer and an A1 Kα radiation as the energy source (1486.6 eV). Binding energy values were corrected for charging by assigning a binding energy of 284.8 eV to the C 1s signal of adventitious carbon. Water contact angles (WCA) were initially determined from digital images obtained with the use of smartphone equipped with macro-lens, by using the drop shape analysis utility of the ImageJ software, particularly the Low-Bond Axisymmetric Drop Shape Analysis (LBADSA) method.^78,79^ The WCA values were further confirmed utilizing an Attension Theta Flex (Biolin Scientific) contact angle meter. Digital images of the magnetic liquid marbles were obtained using a KERN stereoscope equipped with an ODC 87/88 microscope camera. Zeta potential was measured on a suspension of the MOF in water (pH ≈ 7) using a Malvern Zetasizer Nano ZS (Malvern Panalytical, Worcestershire, UK) in a two-electrode capillary cell.

## Supporting Information Available

Miscellaneous Characterization data; Contact angle data in various aqueous media and pH solutions; Pore size distribution results; Characterization data for modified **MOR-1**, **ZIF-8**, **MIL-53** and the hydrophobic magnetic composites; Calculation of the linker deficiencies in the **UiO-66** MOF; Details of oil in water separation properties for the modified cotton fabrics and cotton fiber (PDF)
Video S1 showing the formation of magnetic liquid marbles (MP4)
Video S2 showing the movement of magnetic liquid marbles using an external magnet (MP4)
Video S3 showing the crude oil-in-water separation process (MP4)
