# Correction to “Just Soaping Them: The Simplest Method for Converting Metal Organic Frameworks into Superhydrophobic Materials”

**DOI:** 10.1021/acsami.4c10276

**Published:** 2024-06-27

**Authors:** Dimitrios A. Evangelou, Anastasia D. Pournara, Vasiliki I. Karagianni, Christos Dimitriou, Evangelos K. Andreou, Yiannis Deligiannakis, Gerasimos S. Armatas, Manolis J. Manos

**Affiliations:** † Department of Chemistry, University of Ioannina, Ioannina GR-45110, Greece; ‡ Department of Physics, University of Ioannina, Ioannina GR-45110, Greece; § Department of Materials Science and Technology, University of Crete, Heraklion GR-70013, Greece

The APC Funding Statement is added to the paper.

