# Supplementary material for: Just Soaping Them: The Simplest Method for Converting Metal Organic Frameworks into Superhydrophobic Materials
Source: ACS Appl Mater Interfaces. 2024 Feb 29;16(10):12672–85. doi: 10.1021/acsami.3c19536 (PMC11191008; doi:10.1021/acsami.3c19536)
Supplement: Supplementary file 1 — am3c19536_si_001.pdf [file am3c19536_si_001.pdf]

Supporting Information

Just Soaping Them: The Simplest Method for Converting Metal Organic Frameworks into Superhydrophobic Materials

Dimitrios A. Evangelou,[a] Anastasia D. Pournara,[a,b] Vasiliki I. Karagianni,[a] Christos Dimitriou,[c] Evangelos K. Andreou,[d] Yiannis Deligiannakis,[c] Gerasimos S. Armatas [d] and Manolis J. Manos*,[a]

Corresponding author e-mail: emanos@uoi.gr

[a] Department of Chemistry, University of Ioannina, Ioannina GR-45110, Greece

[b] Present address: Department of Chemistry, Northwestern University, Evanston, 60208, IL, United States (A.D.P.)

[c] Department of Physics, University of Ioannina, Ioannina GR-45110, Greece

[d] Department of Materials Science and Technology, University of Crete, Heraklion GR-70013, Greece

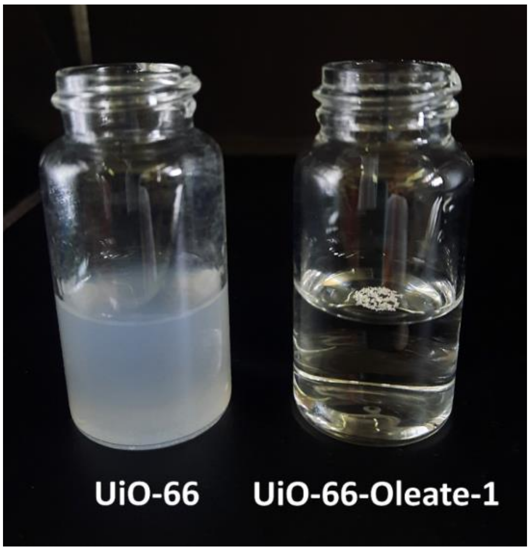

Figure S1. Digital image of **UiO-66** dispersing in water and **UiO-66-Oleate-1** floating on the water surface.

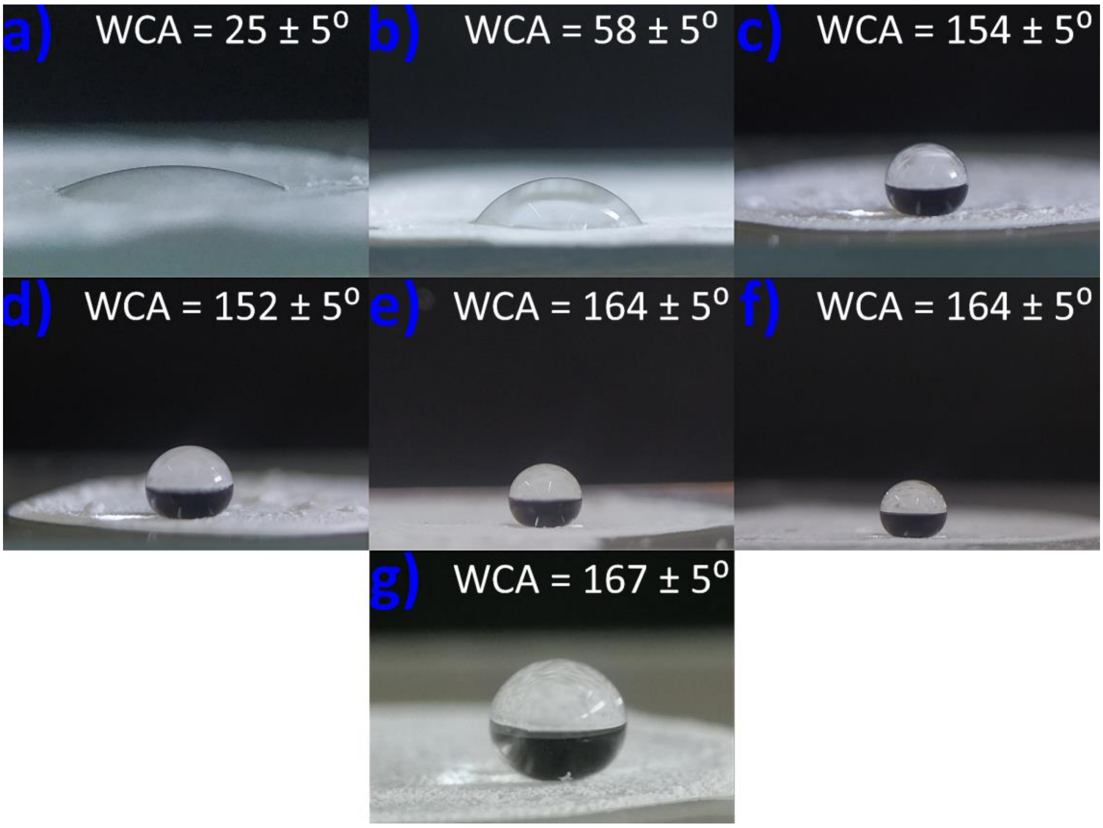

Figure S2. Digital images of water droplets on thin films of a) **UiO-66**, b) **UiO-66-Oleate-6**, c) **UiO-66-Oleate-5**, d) **UiO-66-Oleate-4**, e) **UiO-66-Oleate-3**, f) **UiO-66-Oleate-2** and g) **UiO-66-Oleate-1**, along with the determined WCA values.

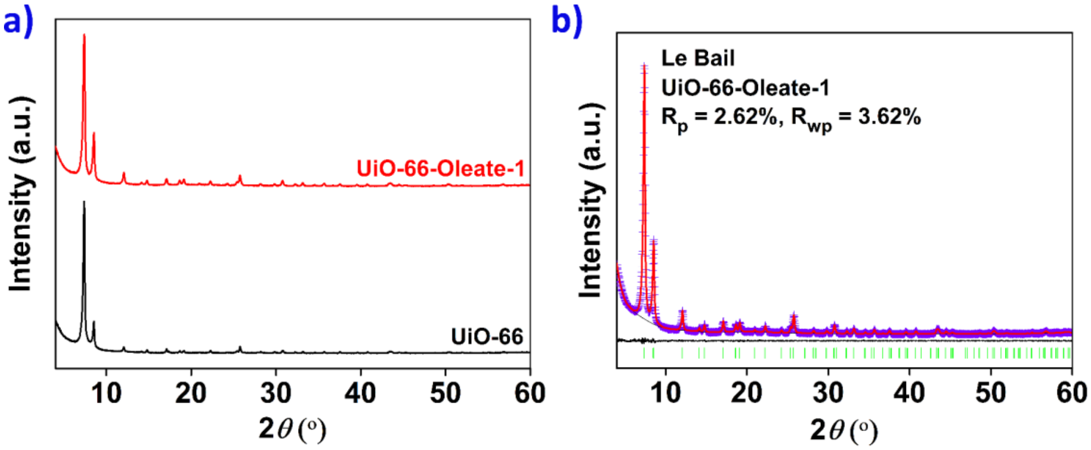

Figure S3. a) Comparative PXRD data for **UiO-66** and **UiO-66-Oleate-1**. b) Le Bail plot of **UiO-66-Oleate-1**. Violet crosses: experimental points; Red line: calculated pattern; Black line: difference pattern (exp. – calc.); Green bars: Bragg positions. Space group: *Fm-3m*. Cell parameters: a = 20.777(6) Å and V = 8969 (8) $Å^3$.

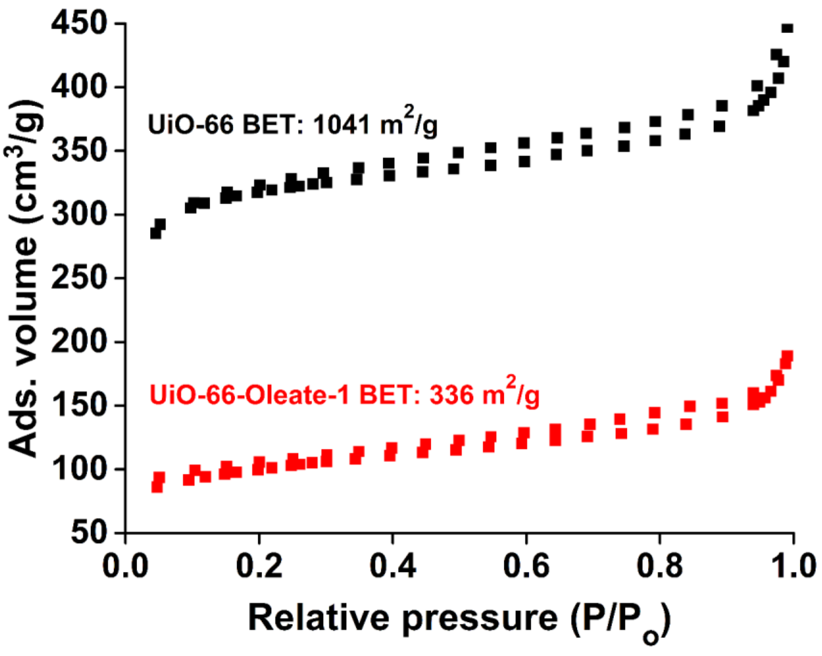

Figure S4. Nitrogen physisorption isotherms at 77 K for the activated **UiO-66** and **UiO-66-Oleate-1**.

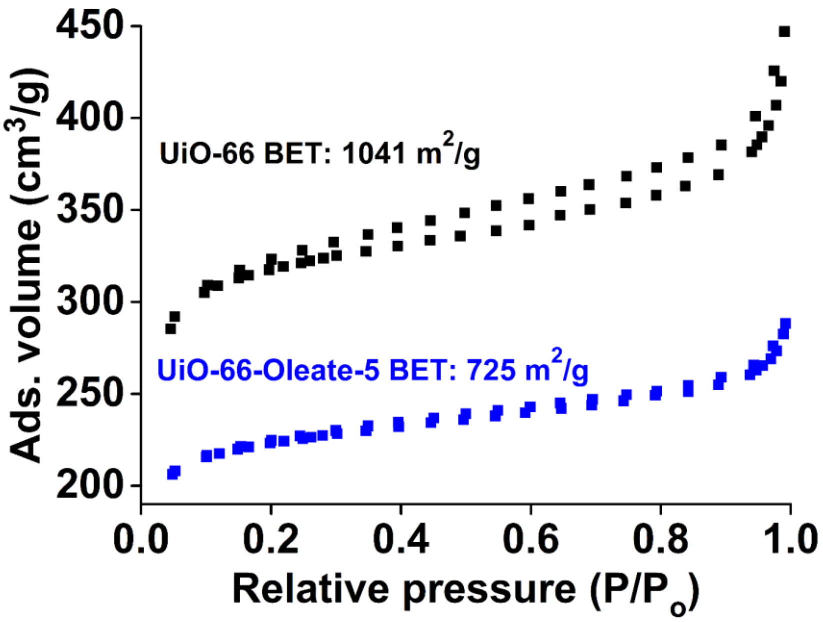

Figure S5. Nitrogen physisorption isotherms at 77 K for the activated **UiO-66** and **UiO-66-Oleate-5**.

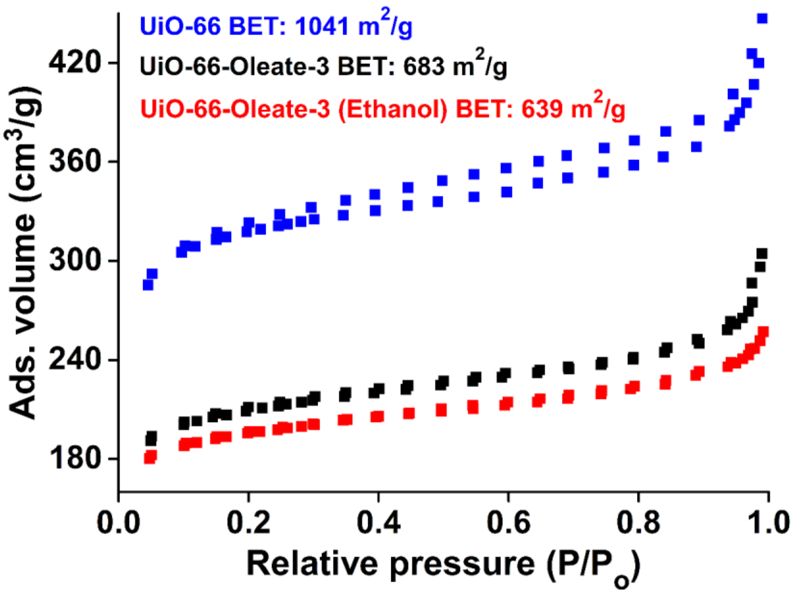

Figure S6. Nitrogen physisorption isotherms at 77 K for the activated **UiO-66**, **UiO-66-Oleate-3** and **UiO-66-Oleate-3 (Ethanol)**.

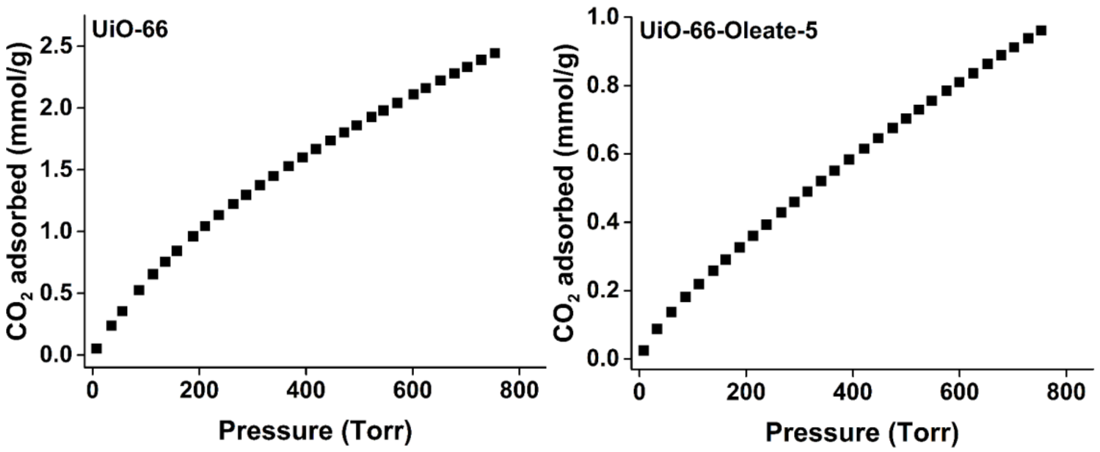

Figure S7. CO_2 adsorption isotherms at 273 K for the activated **UiO-66** and **UiO-66-Oleate-5**.

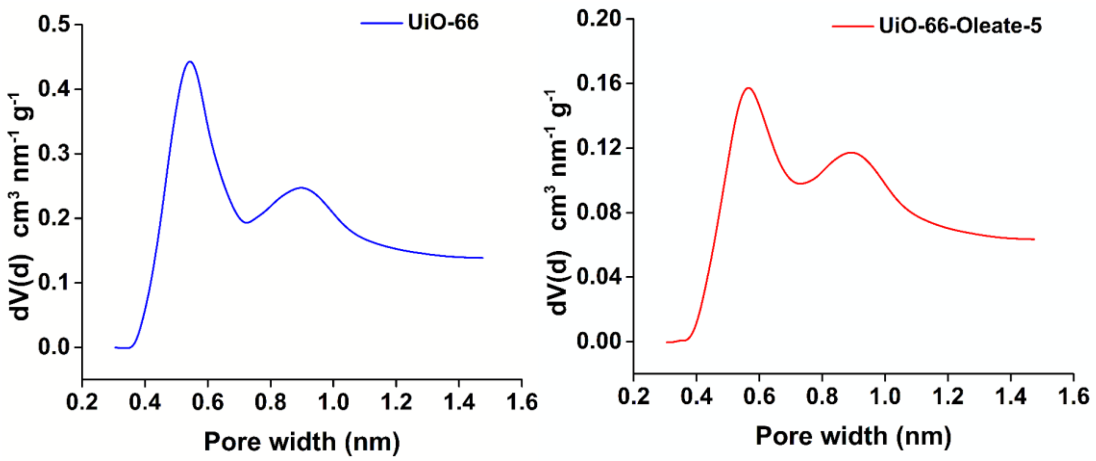

Figure S8. Nonlocal density functional theory (NLDFT) micropore size distribution for **UiO-66** and **UiO-66-Oleate-5**. The NLDFT analysis of the CO_2 adsorption data indicates pore sizes of ~5.6 and ~9 Å for both materials.

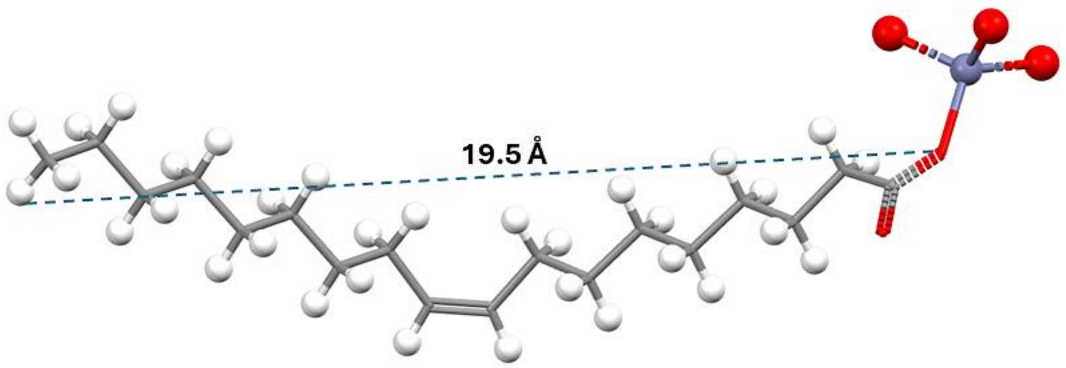

Figure S9. Part of the structure of Zn^{2+}- oleate with the indication of the oleate anion size. C, gray; H, white; O, red; Zn, blue.[1]

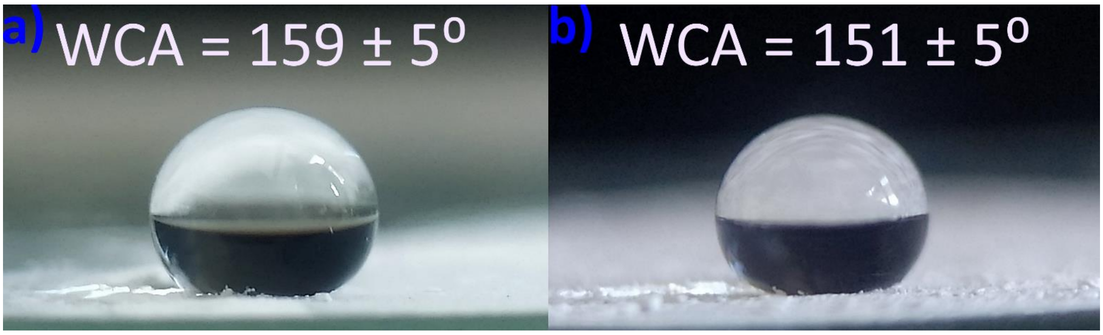

Figure S10. Digital images of water droplets on thin films of a) **UiO-66-Oleate-3 (Ethanol)** and b) **UiO-66-Oleate-5 (Ethanol)**, along with the determined WCA values.

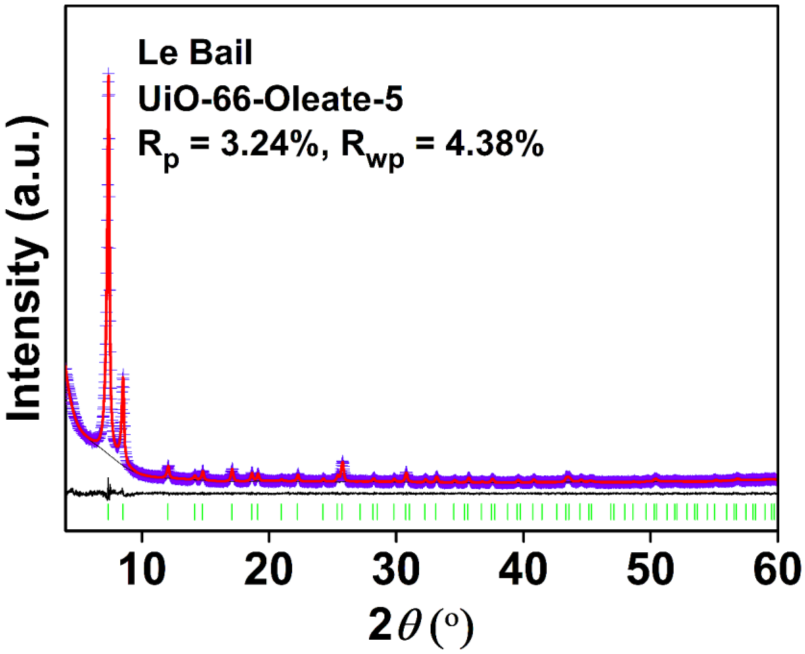

Figure S11. Le Bail plot of **UiO-66-Oleate-5**. Violet crosses: experimental points; Red line: calculated pattern; Black line: difference pattern (exp. – calc.); Green bars: Bragg positions. Space group: *Fm-3m*. Cell parameters: a = 20.759(8) Å and V = 8946(10) $Å^3$.

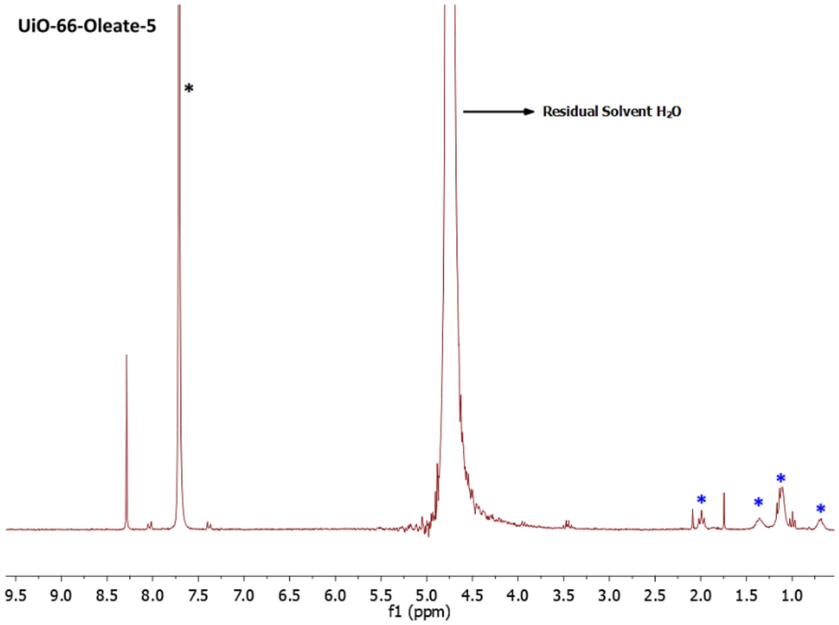

Figure S12. ^{1}H NMR spectrum of **UiO-66-Oleate-5** after digestion in D_2O/NaOH solution. The peaks which are indicated with blue and black asterisks are assigned to oleate and terephthalate ions respectively. Based on the peak integrals the Oleate to MOF molar ratio was determined to be ~ 0.08. The sharp peak at 8.3 ppm is assigned to formic acid which is a base hydrolysis product of the residual DMF solvent.

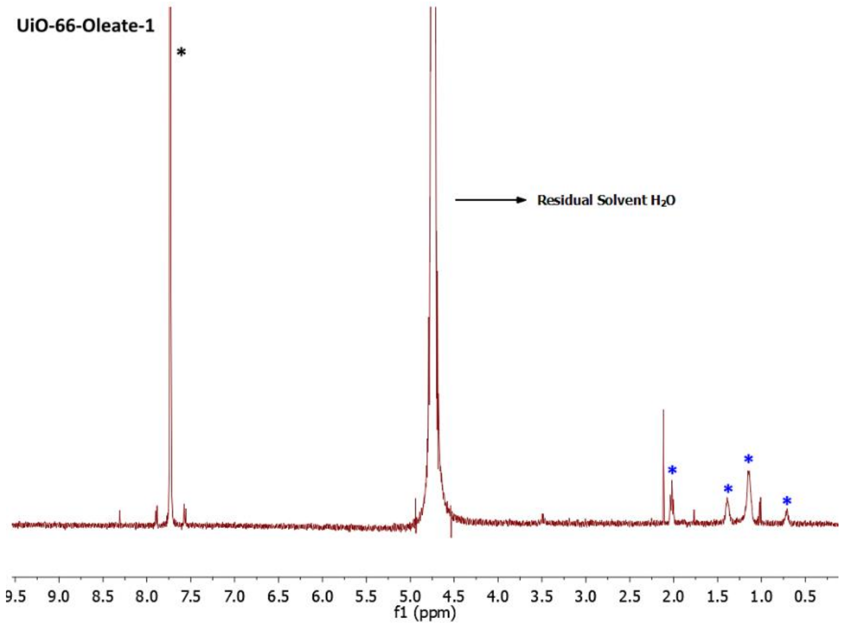

Figure S13. ^{1}H NMR spectrum of **UiO-66-Oleate-1** after digestion in D_2O/NaOH solution. The peaks which are indicated with blue and black asterisks are assigned to oleate and terephthalate ions respectively. Based on the peak integrals the Oleate to MOF molar ratio was determined to be ~ 0.2.

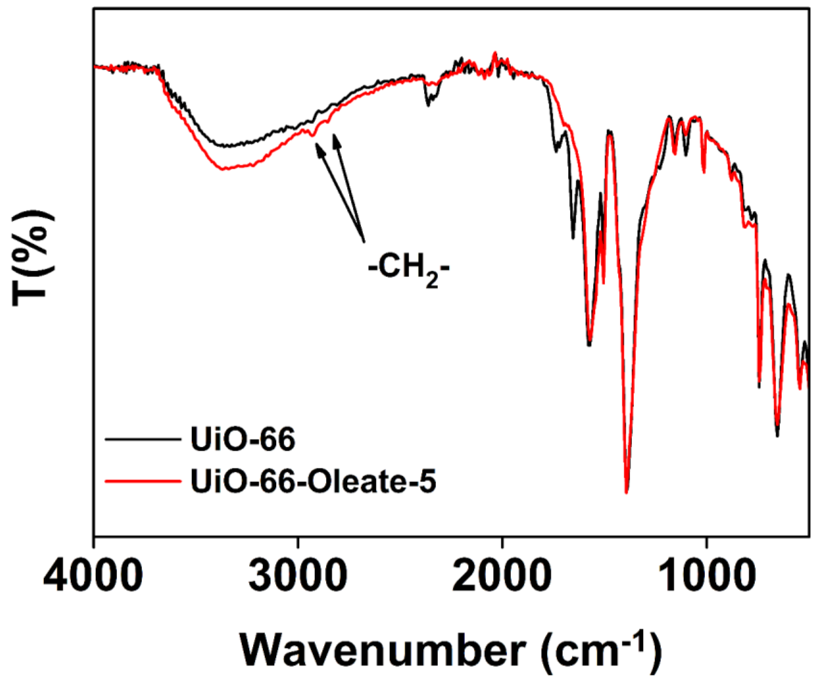

Figure S14. FTIR spectra of **UiO-66** and **UiO-66-Oleate-5**.

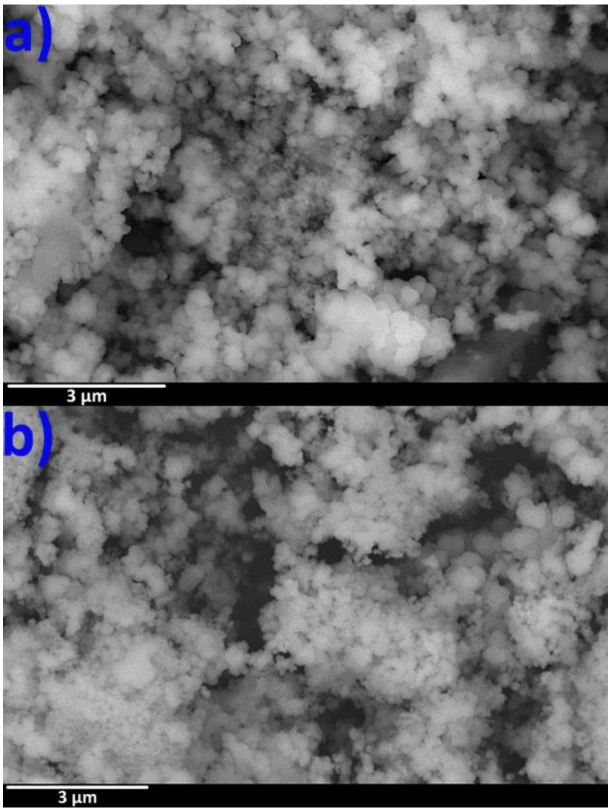

Figure S15. FE-SEM images of a) **UiO-66** and b) **UiO-66-Oleate-5**.

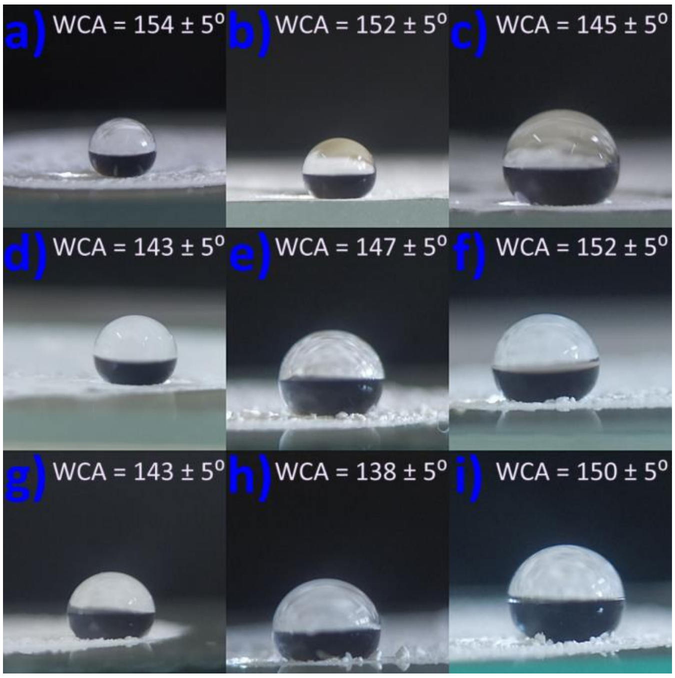

Figure S16. Digital images and obtained WCA values for **UiO-66-Oleate-5** (thin film form) after depositing liquid droplets of a) distilled water, b) tap water, c) lake water, d) sea water, e) aqueous solution pH=0, f) aqueous solution pH=1, g) aqueous solution pH=12 and h) aqueous solution pH=13. i) Digital image and obtained WCA value for **UiO-66-Oleate-5** (thin film form) after degassing at 150 °C.

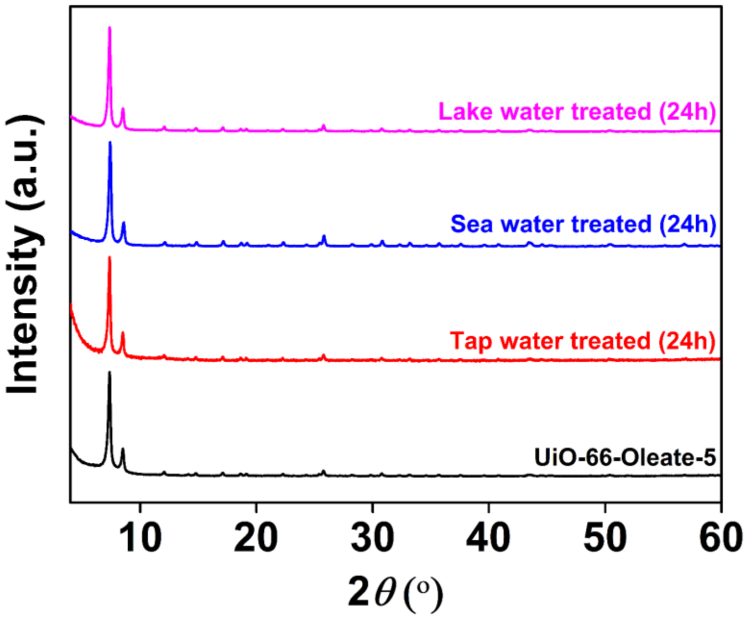

Figure S17. Comparative PXRD data for **UiO-66-Oleate-5** before and after treatment with different aqueous media samples. The results indicate no change in the structural characteristics of **UiO-66-Oleate-5**.

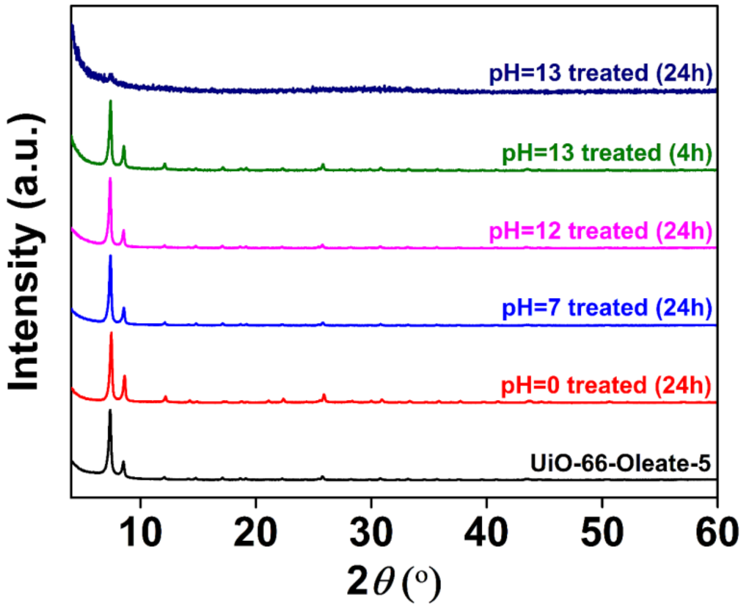

Figure S18. Comparative PXRD data for **UiO-66-Oleate-5** before and after treatment with aqueous solutions of various pH values. The results indicate no change in the structural characteristics of **UiO-66-Oleate-5**. The material preserves its structure even after treatment with an aqueous solution of pH 13; however, extended treatment (24 h) with such solution resulted in loss of the crystallinity of the material.

Calculation of the linker deficiencies in the UiO-66 MOF

The calculation of defects in the **UiO-66** MOF was achieved using TGA data in accordance with the method described to reference.[2] Prior the TGA analysis, the MOF was solvent exchanged with EtOH (as described in section: Activation of **UiO-66 MOFs** prior to N_2 physisorption studies) and then thermally treated at 150 ºC under vacuum for 12 h.

As shown in Figure S19a, it is evident that the **UiO-66** MOF is linker deficient as the percentage of the decomposition weight loss is substantially less than the ideal solvent free, dehydroxylated material (green dashed line marked $\boldsymbol{W_{Ideal.Plat.}}$). $\boldsymbol{W_{End}}$ has been normalized to 100 %, and $\boldsymbol{Wt.PL_{Theo.}}$ is known to be 20.03 %. Thus, only the experimental weight of the TGA plateau ($\boldsymbol{W_{Exp.Plat.}}$) is needed to determine the number of linker deficiencies per $\mathbf{Zr_6}$ formula unit, $\boldsymbol{x}$ according to equation (2). The value of $\boldsymbol{W_{Exp.Plat.}}$ corresponds to the point (plateau) at which everything, except for the terephthalate linkers, has been released from the framework. Based on the TGA curve the plateau is reached at 365 ºC ($\boldsymbol{T_{Plat.}}$) (Figure S19a). Hence, the experimental plateau weight ($\boldsymbol{W_{Exp.Plat.}}$) , which represents the normalized weight of the material at 365 °C, is 169 % (Figure S19a).

According to equation (1) the value of $\boldsymbol{NL_{Exp.}}$ is calculated as:

$$\boldsymbol{NL_{Exp.} = 6 - x = \left(\frac{W_{Exp.Plat.} - W_{End}}{Wt.PL_{Theo.}}\right)}$$

$$\boldsymbol{= 6 - x = \left(\frac{169 - 100\%}{20.03\%}\right)}$$

$$\boldsymbol{= 3.45}$$

According to equation (2) the linker deficiencies ($\boldsymbol{x}$) is calculated as:

$$\boldsymbol{x = 6 - 3.45 = 2.55}$$

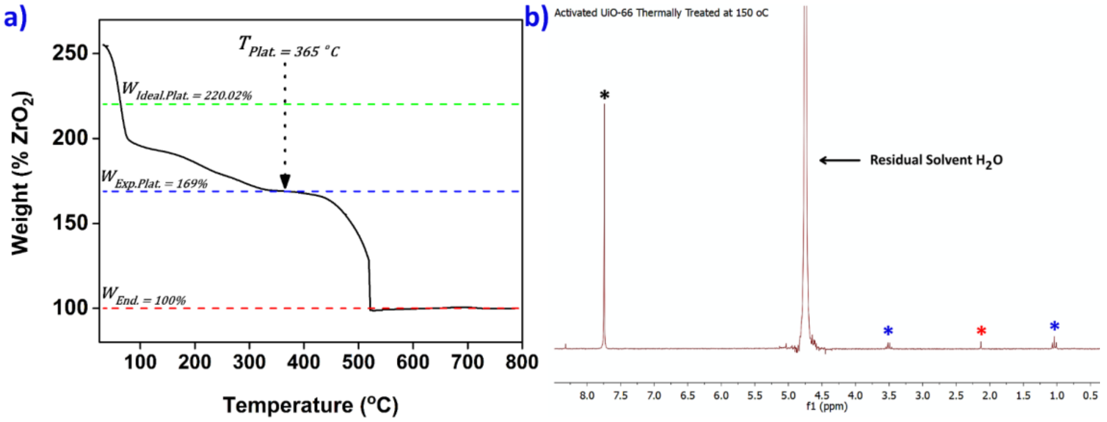

Figure S19. a) TGA data for the **UiO-66** MOF measured in air (normalized such that end weight = 100%). The vertical dashed line represents $\boldsymbol{T_{Plat.}}$, the temperature at

which the plateau ($\boldsymbol{W_{Exp.Plat.}}$) is reached. The horizontal dashed lines correspond to the relevant TGA plateaus. b) ^{1}H NMR spectrum of the **UiO-66** MOF after digestion in D_2O/NaOH solution. The peaks which are indicated with black asterisks are assigned to terephthalate anions. The blue and red asterisks refer to EtOH and Dimethylamine traces respectively.

In addition, ^{1}H NMR data (Figure S19b) for the **UiO-66** MOF, after solvent exchange and thermal treatment at 150 °C for 12 h, revealed no modulator residues, such as formic acid that could be resulted from DMF decomposition. The general formula for a defective Zr^{4+} MOF of the UiO type, with no modulators as co-ligands, can be expressed as $Zr_6O_4(OH)_{4+2x}(H_2O)_{2x}(L)_{6-x}$, where each missing linker is replaced by two H_2O and two OH^- terminal ligands. Thus, the chemical formula of the **UiO-66** MOF, considering the results from TGA, is **$[Zr_6O_4(OH)_{9.1}(H_2O)_{5.1}(L)_{3.45}]$•solvents**

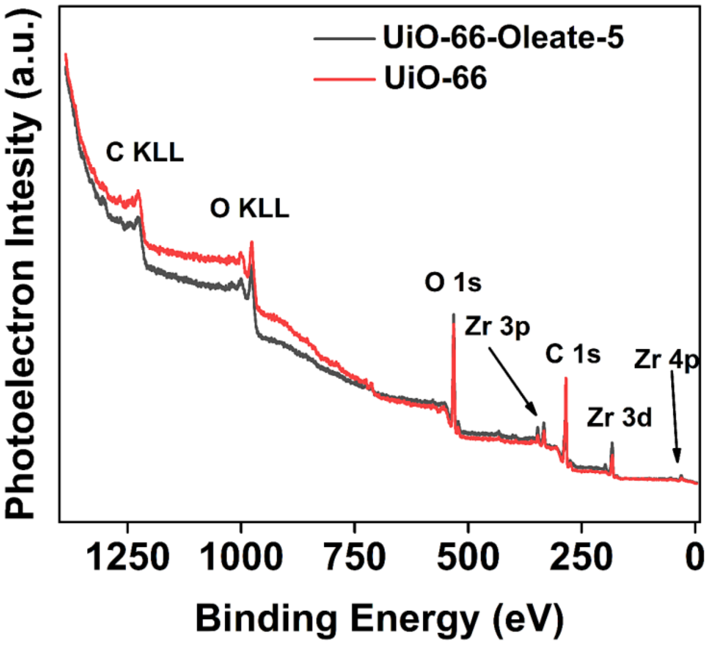

Figure S20. XPS survey spectra of **UiO-66** and **UiO-66-Oleate-5**.

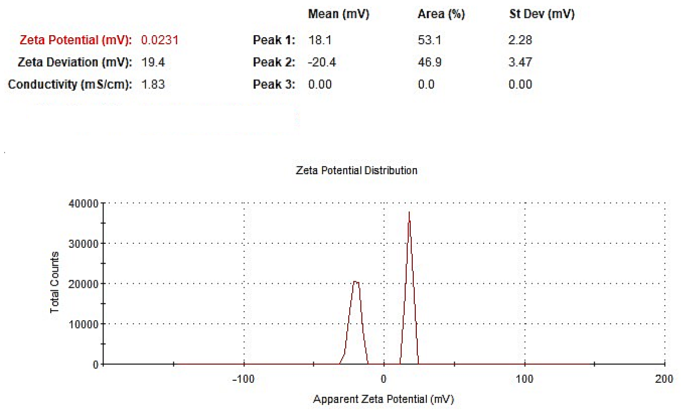

Figure S21. Zeta potential distribution of **UiO-66** in double distilled water (pH ~ 7).

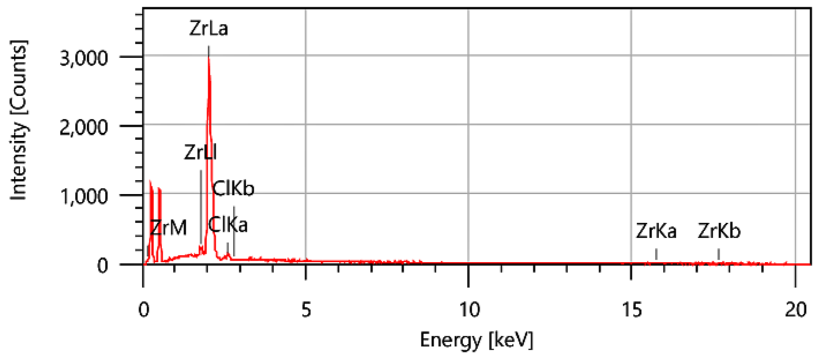

Figure S22. EDS spectrum of **UiO-66-Oleate-5**.

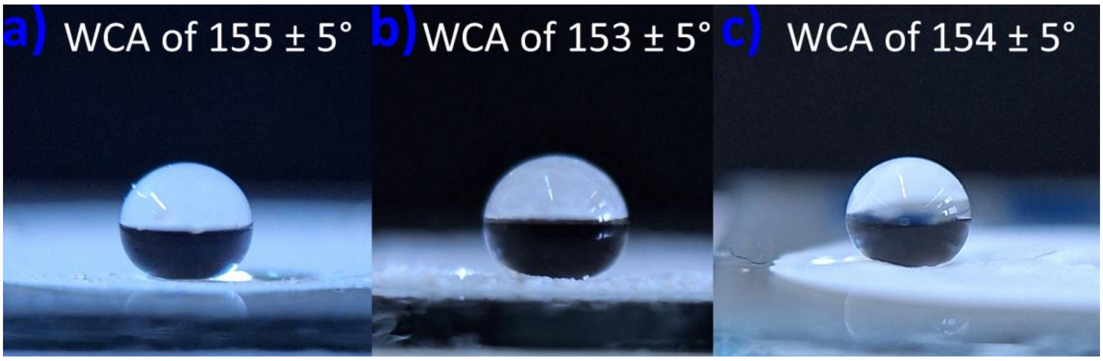

Figure S23. a) Digital images of water droplets on thin films of a) **MOR-1-Oleate**, b) **MIL-53(Al)-Oleate** and c) **ZIF-8-Oleate**, along with the determined WCA values.

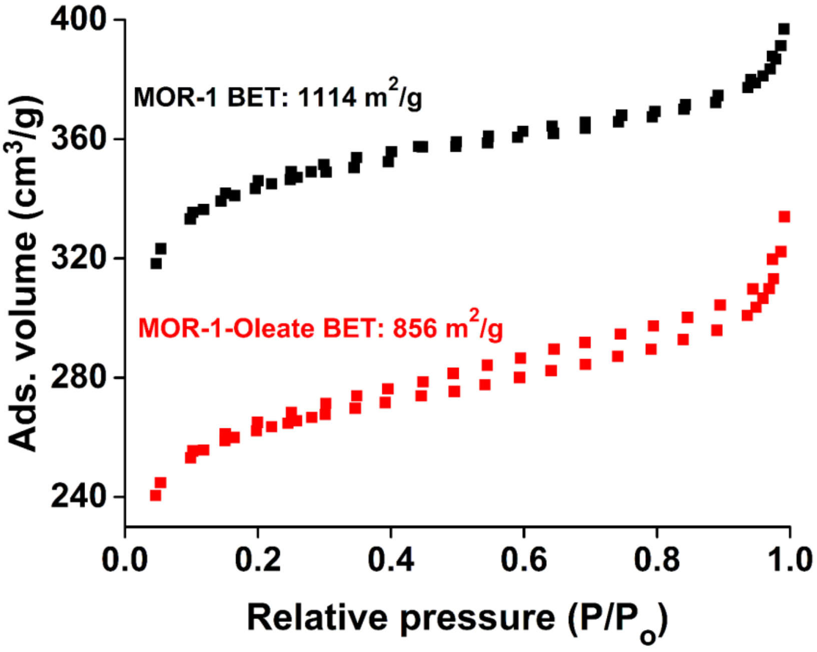

Figure S24. Nitrogen physisorption isotherms at 77 K for the activated **MOR-1** and **MOR-1-Oleate**.

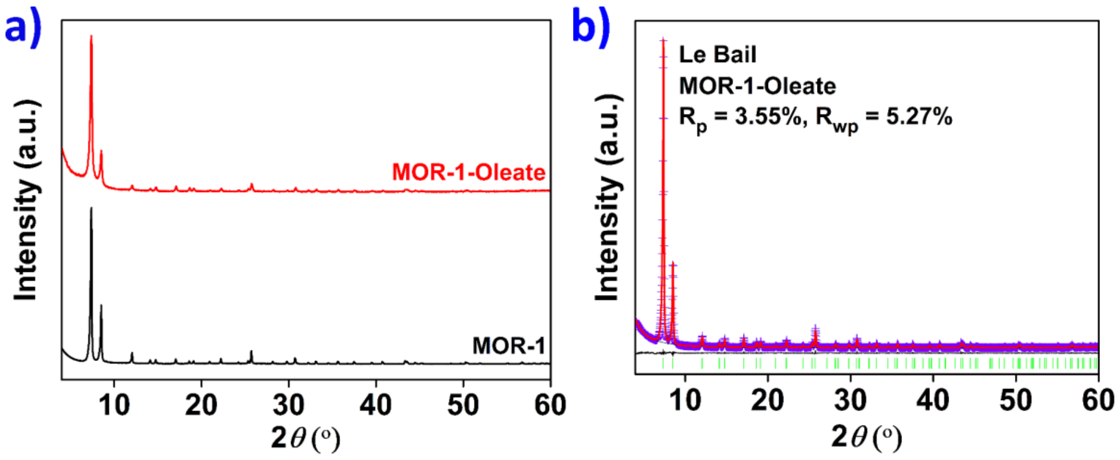

Figure S25. a) Comparative PXRD data for **MOR-1** and **MOR-1-Oleate**. b) Le Bail plot of **MOR-1-Oleate**. Violet crosses: experimental points; Red line: calculated pattern; Black line: difference pattern (exp. – calc.); Green bars: Bragg positions. Space group: *Fm-3m*. Cell parameters: a = 20.758(2) Å and V = 8944(2) Å^3.

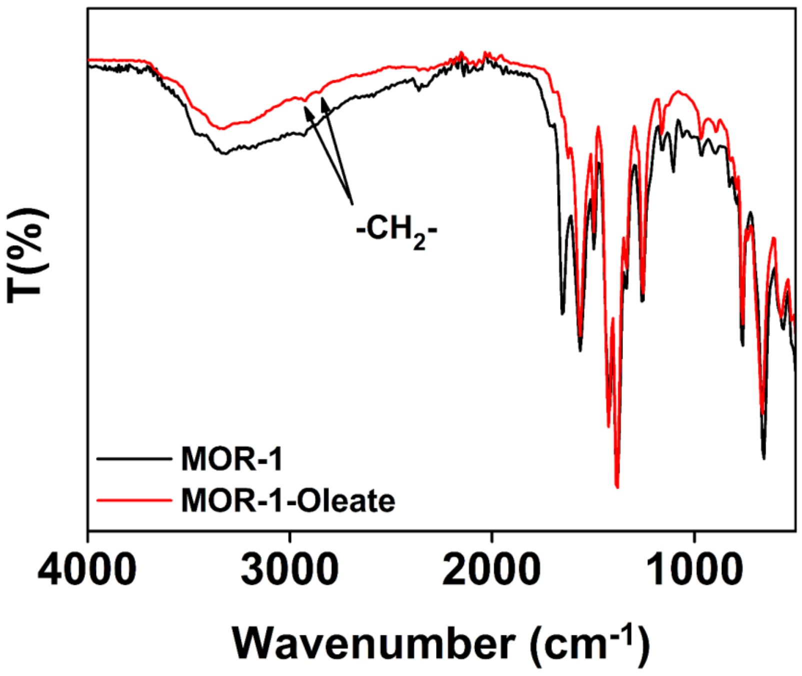

Figure S26. FTIR spectra of **MOR-1** and **MOR-1-Oleate**.

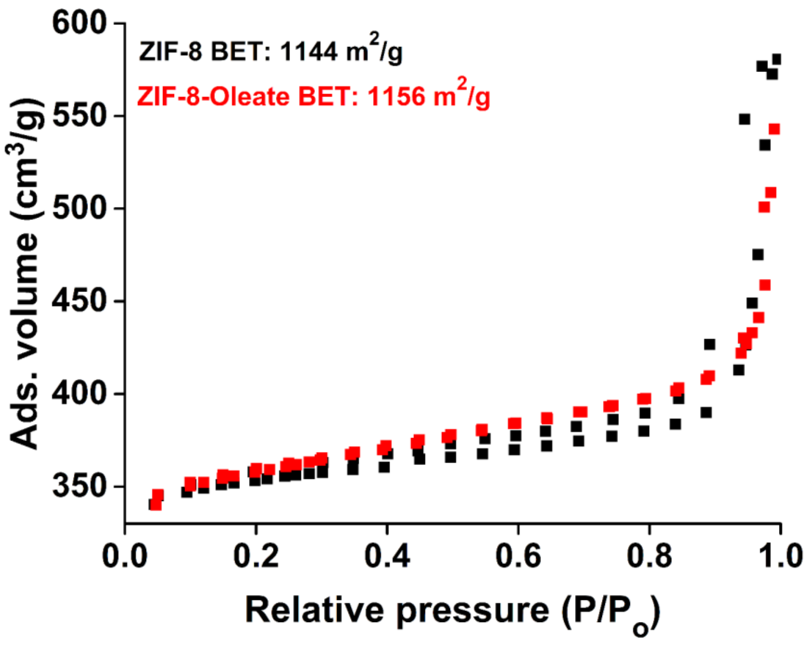

Figure S27. Nitrogen physisorption isotherms at 77 K for the activated **ZIF-8** and **ZIF-8-Oleate**.

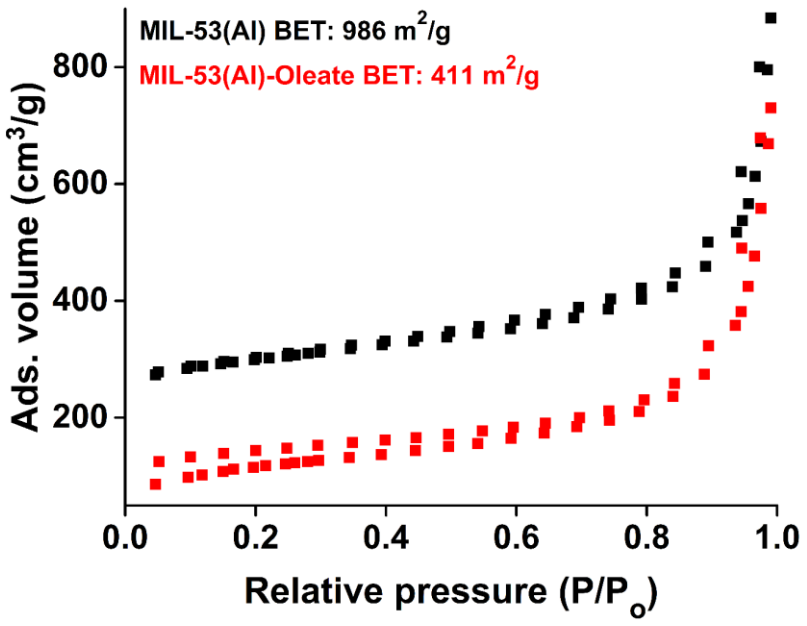

Figure S28. Nitrogen physisorption isotherms at 77 K for the activated **MIL-53** and **MIL-53-Oleate**.

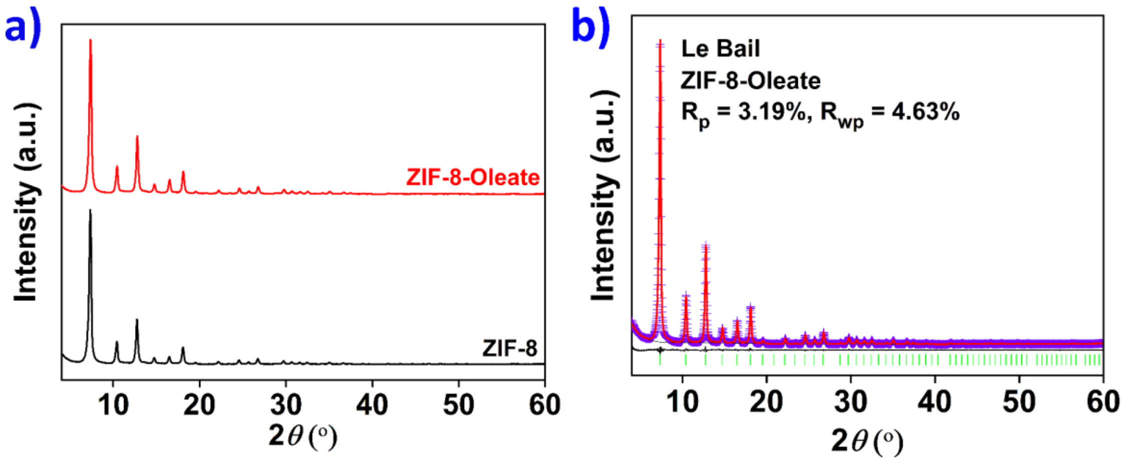

Figure S29. a) Comparative PXRD data for **ZIF-8** and **ZIF-8-Oleate**. b) Le Bail plot of **ZIF-8-Oleate**. Violet crosses: experimental points; Red line: calculated pattern; Black line: difference pattern (exp. – calc.); Green bars: Bragg positions. Space group: *I-43m*. Cell parameters: a = 17.012(4) Å and V = 4924(4) $Å^3$.

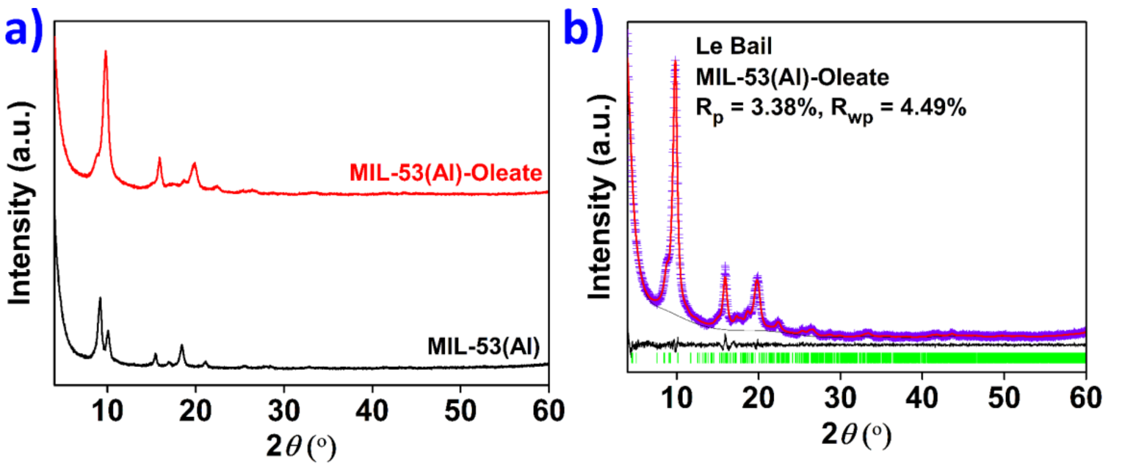

Figure S30. a) Comparative PXRD data for **MIL-53(Al)** and **MIL-53(Al)-Oleate**. b) Le Bail plot of **MIL-53(Al)-Oleate**. Violet crosses: experimental points; Red line: calculated pattern; Black line: difference pattern (exp. – calc.); Green bars: Bragg positions. Space group: *P1*. Cell parameters: a = 22.4(1), b = 23.8(1), c = 6.52(4) Å, α = 91.99(7), β = 108.2(1), γ = 57.1(1) ° and V = 2733(27) $Å^3$.

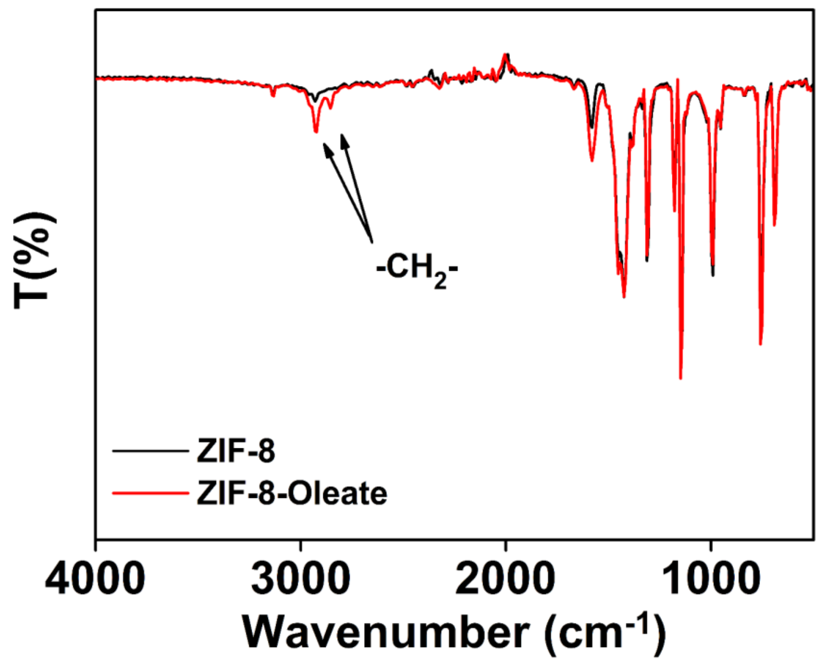

Figure S31. FTIR spectra of **ZIF-8** and **ZIF-8-Oleate**.

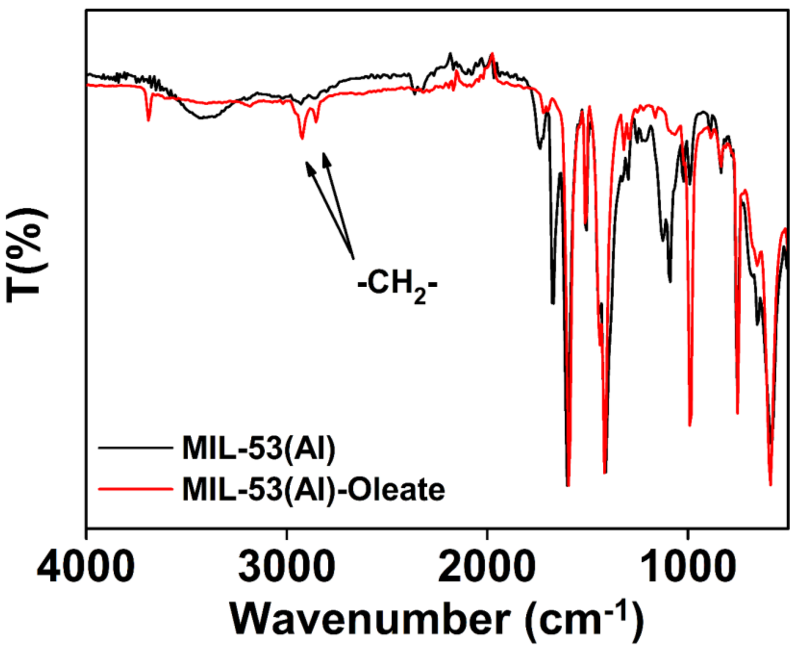

Figure S32. FTIR spectra of **MIL-53(Al)** and **MIL-53(Al)-Oleate**.

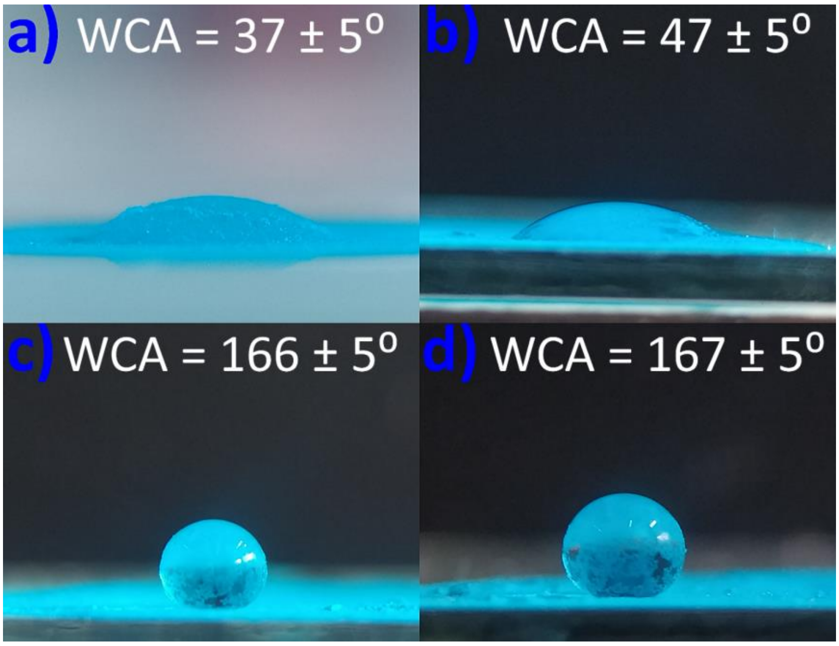

Figure S33. Digital images of water droplets on thin films of a) **HKUST-1**, b**) HKUST-1-Oleate-3**, c) **HKUST-1-Oleate-2** and d) **HKUST-1-Oleate-1**, along with the determined WCA values.

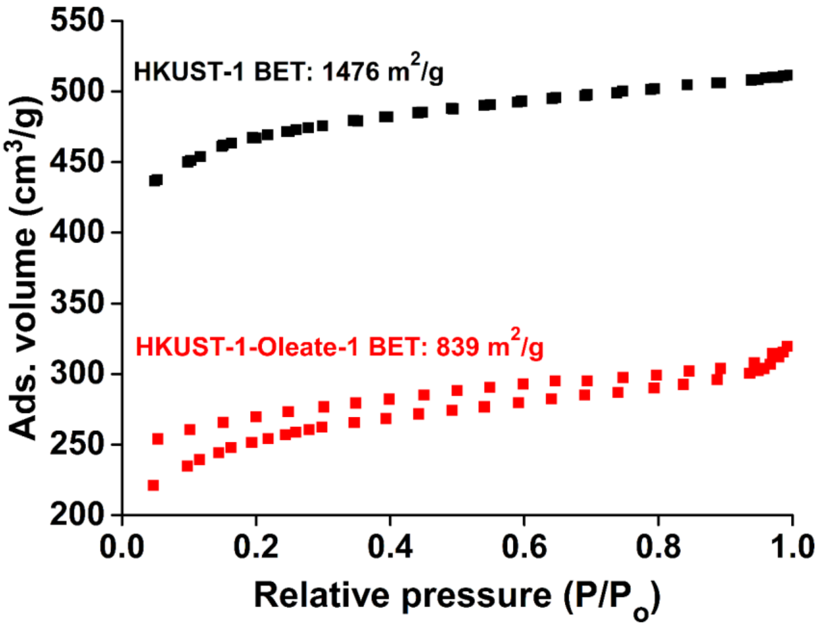

Figure S34. Nitrogen physisorption isotherms at 77 K for **HKUST-1** and **HKUST-1-Oleate-1**.

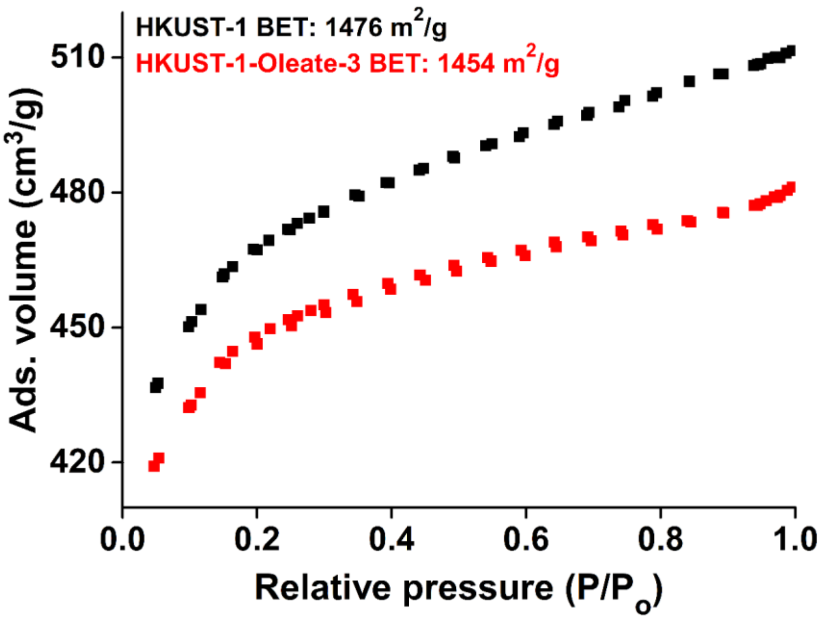

Figure S35. Nitrogen physisorption isotherms at 77 K for **HKUST-1** and **HKUST-1-Oleate-3**.

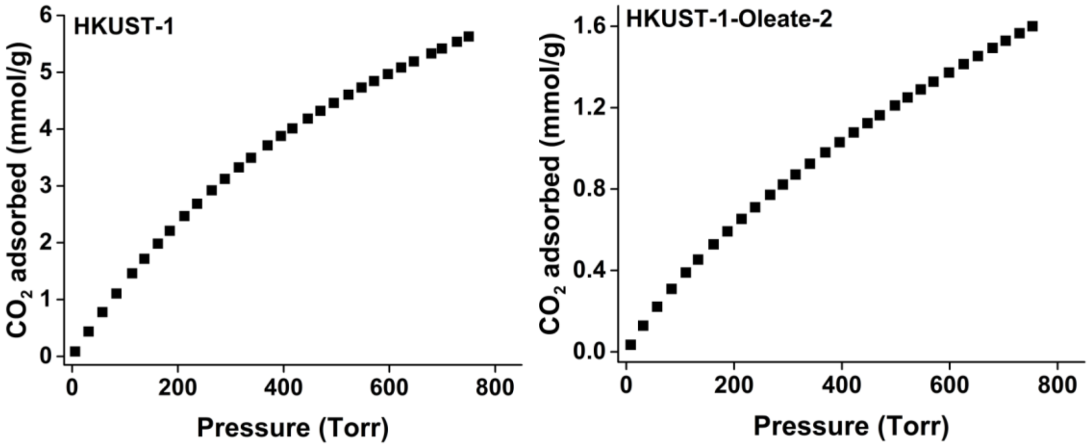

Figure S36. CO_2 adsorption isotherms at 273 K for the activated **HKUST-1** and **HKUST-1-Oleate-2**.

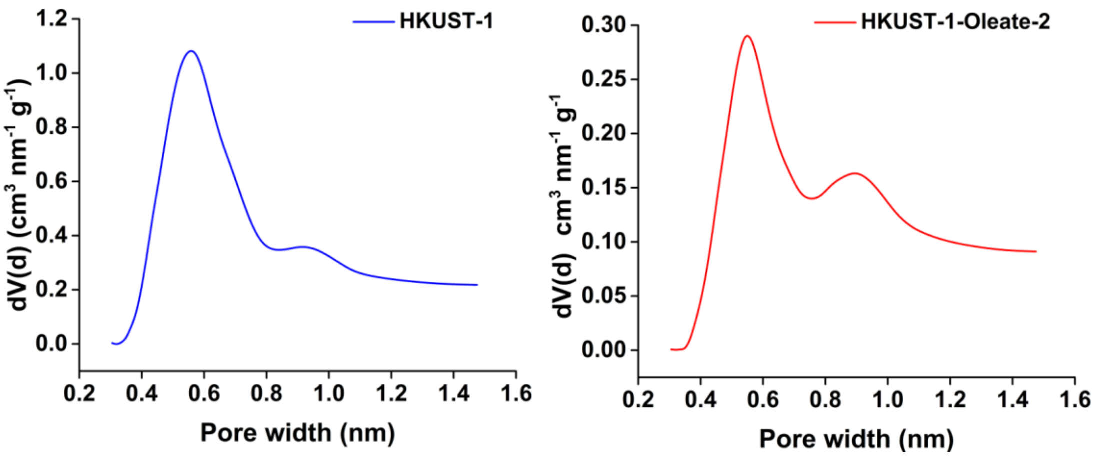

Figure S37.

Figure S37. Nonlocal density functional theory (NLDFT) micropore size distribution for **HKUST-1** and **HKUST-1-Oleate-2**. The NLDFT analysis of the CO_2 adsorption data indicates pore sizes of ~5.5 and ~9.1 Å for both materials.

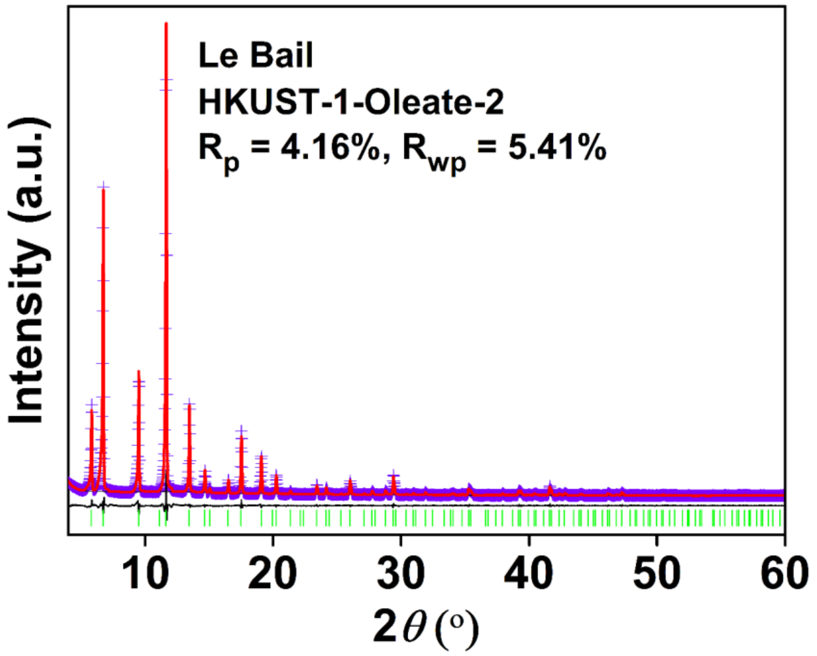

Figure S38. Le Bail plot of **HKUST-1-Oleate-2.** Violet crosses: experimental points; Red line: calculated pattern; Black line: difference pattern (exp. – calc.); Green bars: Bragg positions. Space group: *Fm-3m*. Cell parameters: a = 26.230(1) Å and V = 18191(2) $Å^3$.

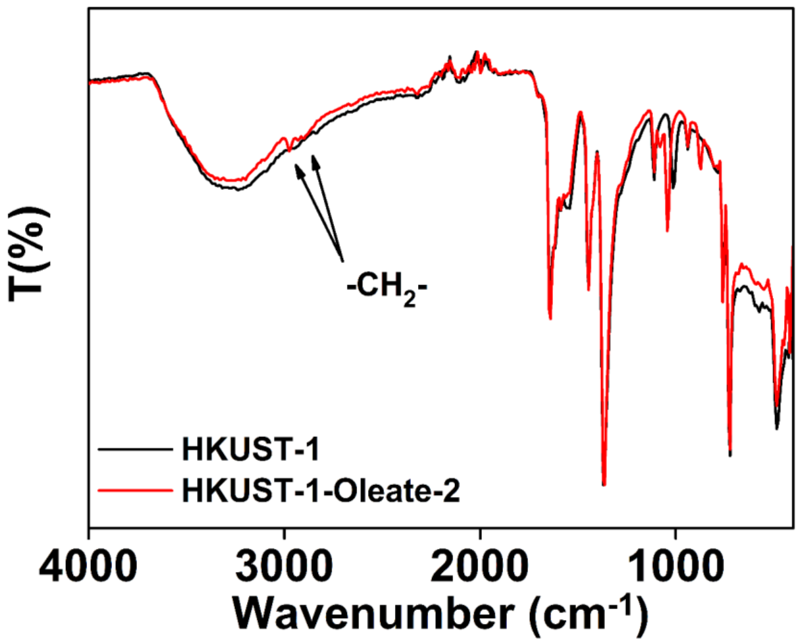

Figure S39. FTIR spectra of **HKUST-1** and **HKUST-1-Oleate-2**.

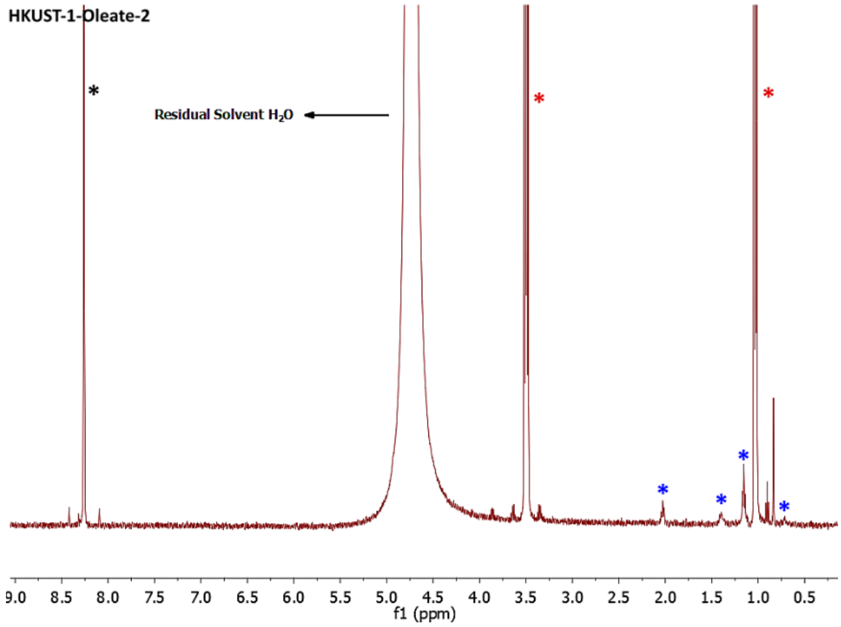

Figure S40. ^{1}H NMR spectrum of **HKUST-1-Oleate-2** after digestion in D_2O/NaOH solution. The peaks which are indicated with blue and black asterisks are assigned to oleate and trimesate ions respectively. The read asterisks refer to ethanol signals. Based on the peak integrals the Oleate to MOF molar ratio was determined to be ~ 0.07.

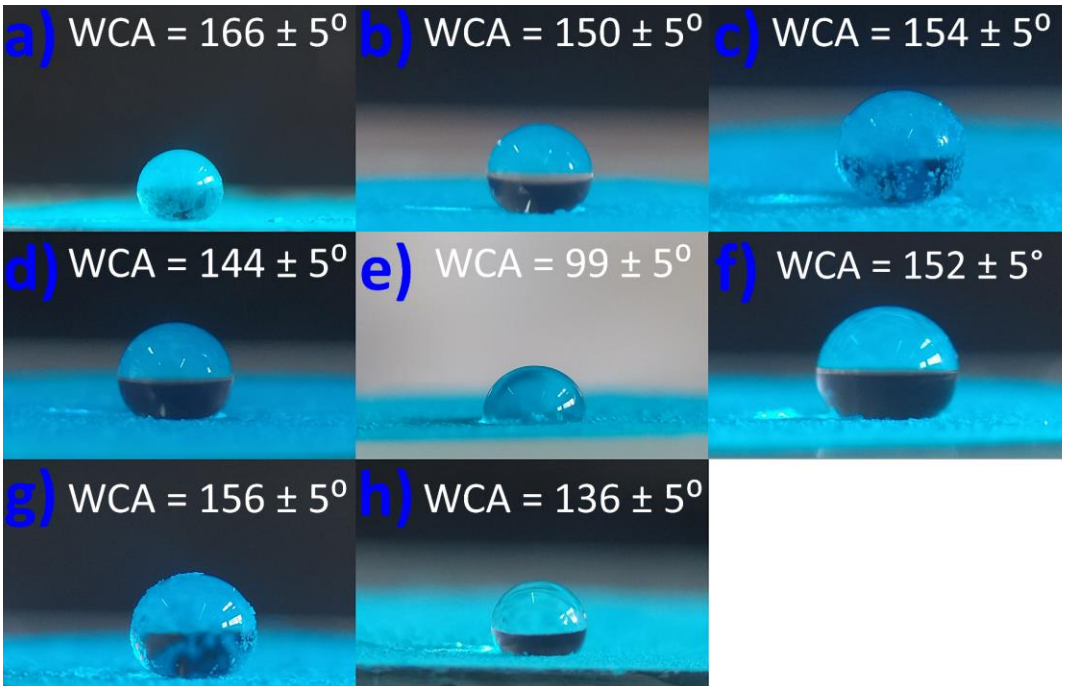

Figure S41. Digital images and obtained WCA values for **HKUST-1-Oleate-2** (thin film form) after depositing liquid droplets of a) distilled water, b) tap water, c) lake water, d) sea water, e) aqueous solution pH=2, f) aqueous solution pH=3 and g) aqueous solution pH=10. h) Digital image and obtained WCA value for **HKUST-1-Oleate-2** (thin film form) after degassing at 120 °C.

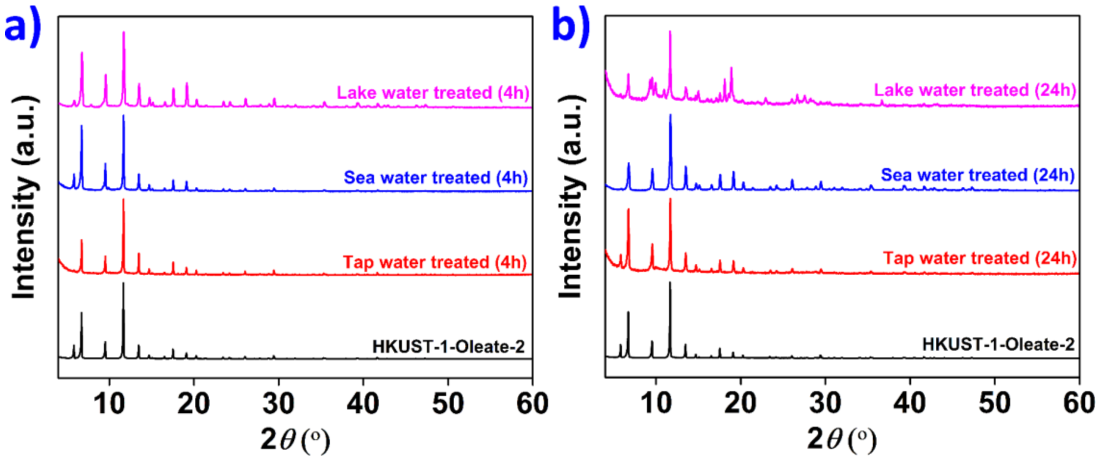

Figure S42. Comparative PXRD data for **HKUST-1-Oleate-2** before and after treatment with different aqueous media samples for 4 h (a) and 24 h (b). The results indicate that the structural characteristics of **HKUST-Oleate-2** are largely retained after treatment with various aqueous media for 4 hours. The structure of **HKUST-Oleate-2** remains stable even after a 24-hour treatment with tap water and sea water. However, the 24-hour treatment of the material with lake water appears to lead to partial decomposition of the MOF.

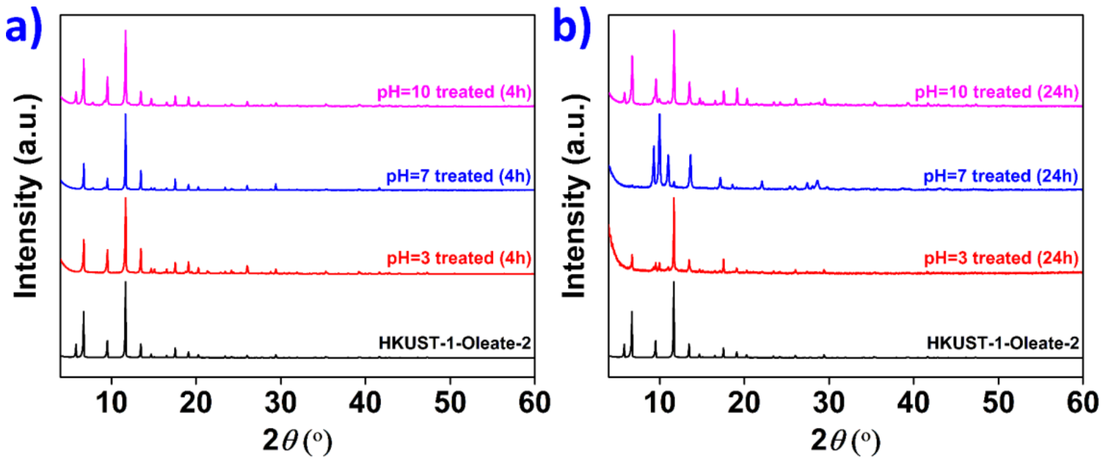

Figure S43. Comparative PXRD data for **HKUST-1-Oleate-2** before and after treatment with aqueous solutions of various pH values for 4 h (a) and 24 h (b). The results indicate that the structural characteristics of **HKUST-Oleate-2** are largely retained after treatment with various pH aqueous solutions for 4 hours. The structure of **HKUST-Oleate-2** remains stable even after a 24-hour treatment with the alkaline solution. However, the 24-hour treatment of the material with solutions of pH 7 and pH 3 appears to lead to partial decomposition of the MOF.

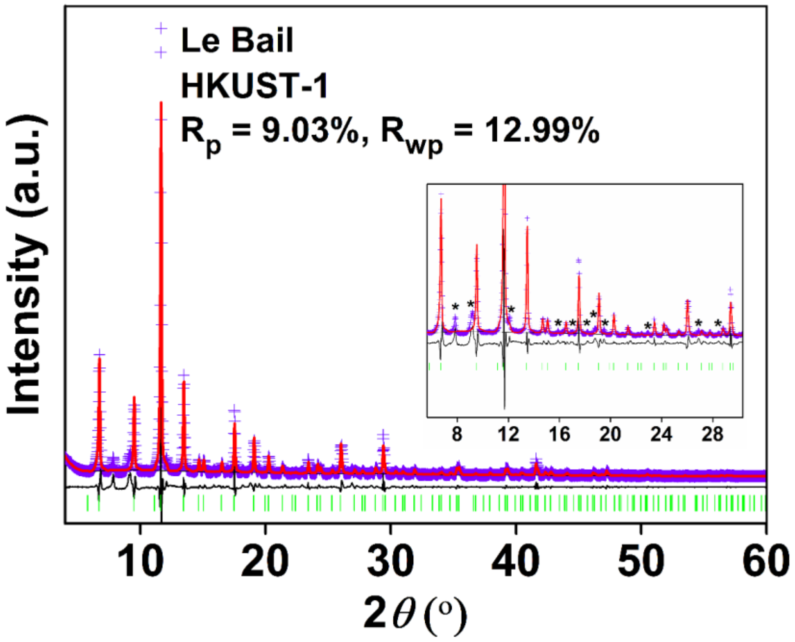

Figure S44. Le Bail plot of pristine **HKUST-1** (microcrystalline powder) exposed in water vapors for 3 days. Violet crosses: experimental points; Red line: calculated pattern; Black line: difference pattern (exp. – calc.); Green bars: Bragg positions. Space group: *Fm-3m*. Cell parameters: a = 26.314(5) Å and V = 18220(11) Å^3. Inset: magnification of the 2θ region 4 - 30°. The diffraction peaks, which are indicated with asterisk in the PXRD pattern, are not assigned to the original **HKUST-1**.

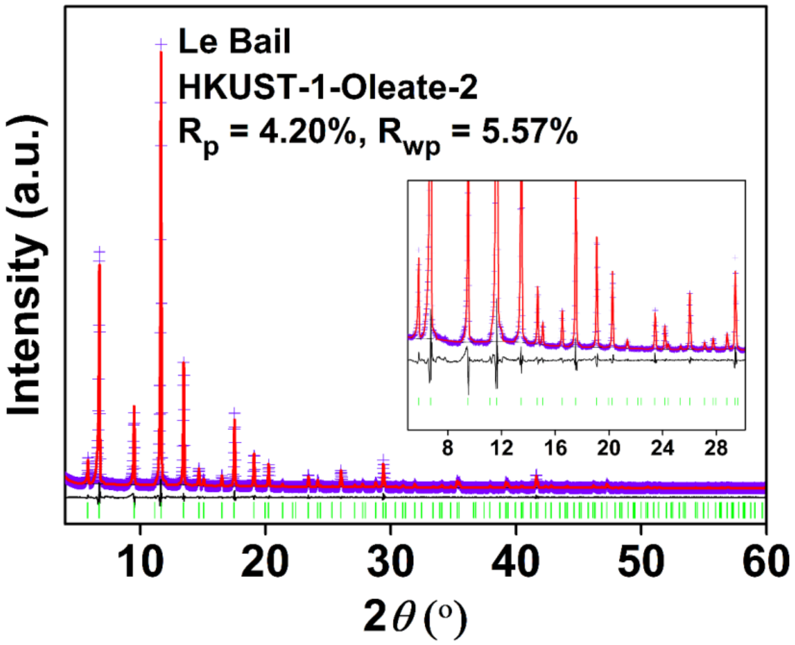

Figure S45. Le Bail plot of **HKUST-1-Oleate-2** exposed in water vapors for 3 days. Violet crosses: experimental points; Red line: calculated pattern; Black line: difference pattern (exp. – calc.); Green bars: Bragg positions. Space group: *Fm-3m*. Cell parameters: a = 26.2893(5) Å and V = 18169(1) $Å^3$. Inset: magnification of the 2θ region 4 - 30°.

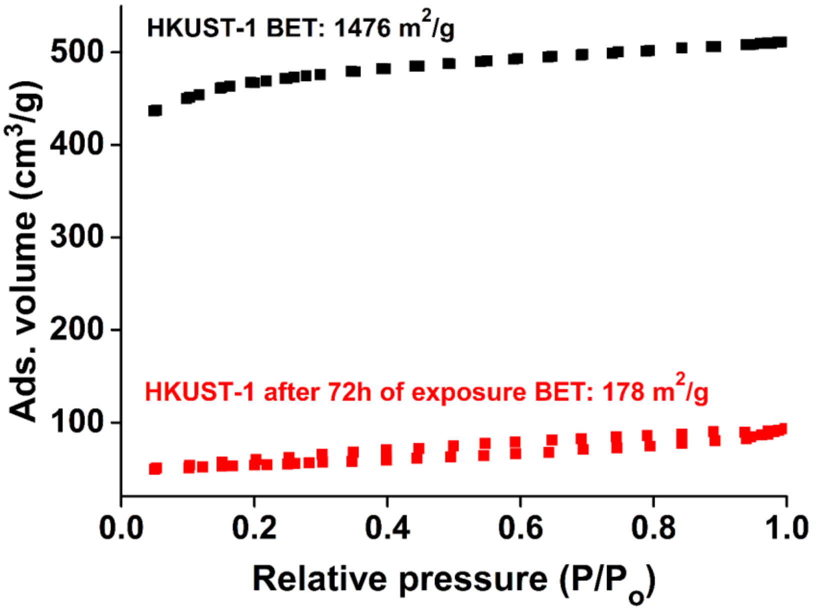

Figure S46. Nitrogen physisorption isotherms at 77 K for **HKUST-1** before and after the exposure in humid conditions for 72h.

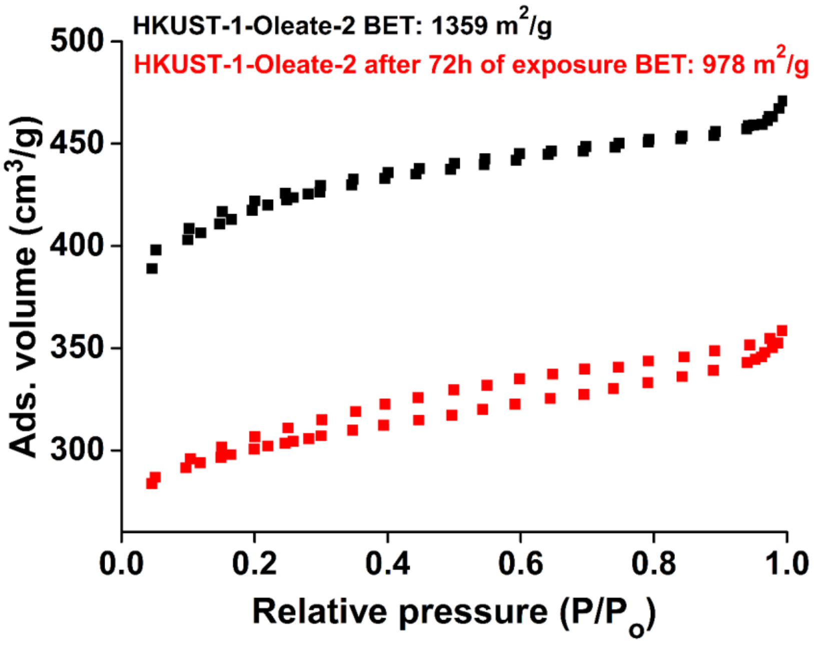

Figure S47. Nitrogen physisorption isotherms at 77 K for **HKUST-1-Oleate-2** before and after the exposure in humid conditions for 72h.

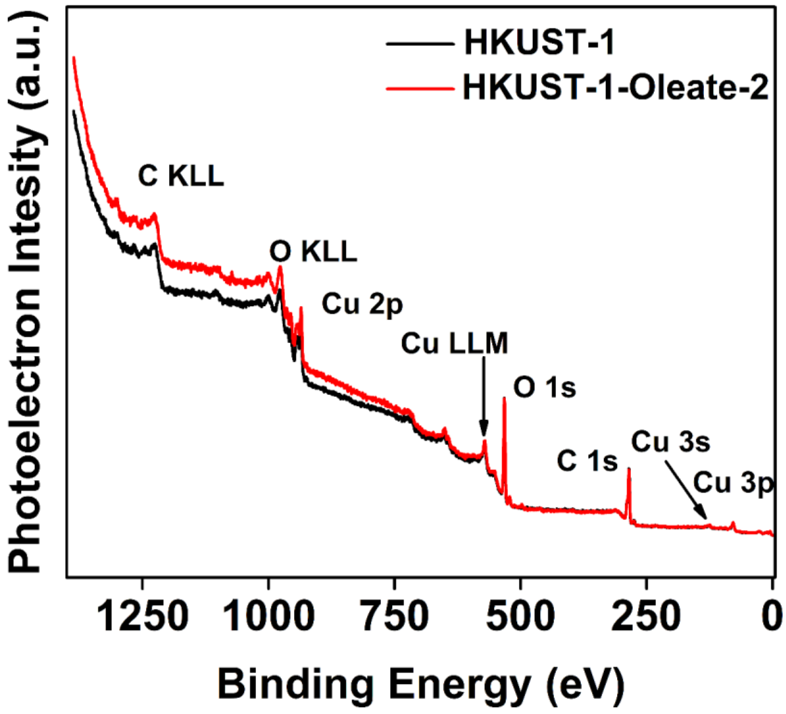

Figure S48. XPS survey spectra of **HKUST-1** and **HKUST-1-Oleate-2**.

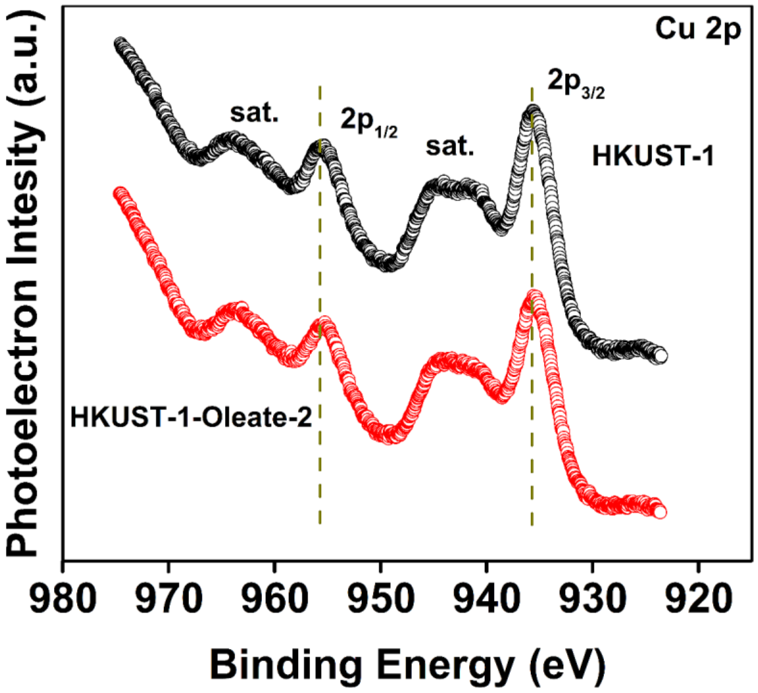

Figure S49. High resolution Cu 2p core-level photoelectron spectra of **HKUST-1** and **HKUST-1-Oleate-2**. The peaks assigned to Cu $2p_{3/2}$ and $2p_{1/2}$ appear at 935.5/955.3 and 935.3/955.1 eV respectively.

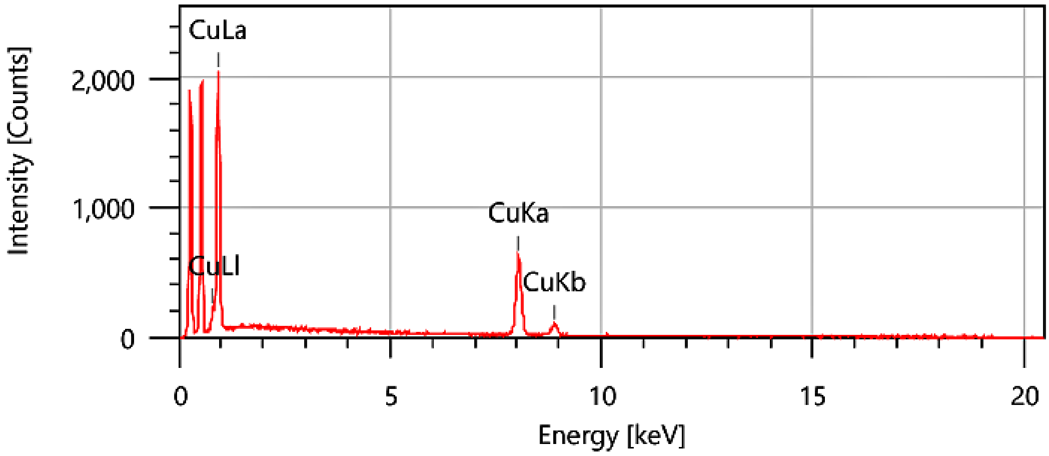

Figure S50. EDS spectrum of **HKUST-1-Oleate-2**.

Table S1. Comparison of selected features of our method with known methods for the conversion of MOFs into superhydrophobic materials.

MOF based material/composite	Modification method	WCA (°)	Cost of reagents /solvents used	No of synthetic steps and Duration/Temperature	Solvents used	Use of fluorinated reagents	% Porosity Retained	Special conditions	Application to stable supports	Ref.
HBCF	Modification with FAS-17	154.5 ± 2	High	1. 15 min/room temp.<br>2. 10 min/room temp.<br>3. 30 min/room temp.	1. NaOH<br>2. H_2O/EtOH<br>3. CH_3Cl_3	Yes	-	Electrochemical apparatus	Copper foam	3
MF-Cu/ZIF-67@PDMS Sponge	PDMS Coating	158.5 (acidic pH)	Moderate	1. 15 s/room temp.<br>2. a) 15 s/room temp.<br>b) 3h/160 °C	1. n-Hexane<br>2. n-Hexane	No	-	No	Melamine sponge	4
AlTz-68-C18-1h	Modification with 1-octadecene	173.6	Moderate	1. 1 h/30 °C<br>2. 1 h/40 °C	1. Diethyl ether<br>2. Diethyl ether	No	91	No	Melamine sponge	5
ZIF-8*/FTN/Kapok aerogel	Modification with 1-dodecane	162.3	Moderate	1. 1 h/80 °C<br>2. 1 min/room temp.<br>3. 6 h/50 °C	1. H_2O<br>2. H_2O<br>3. MeOH	No	2.7	No	Raw kapok	6
PDMS-MZIF15-PA	PDMS Coating	158.6	Moderate	1. 60 min/room temp.<br>2. 15 min/room temp.<br>3. a) 12 min/room temp.<br>b) 30 min/70 °C	1. H_2O<br>2. H_2O<br>3. n-Hexane	No	-	CO_2 laser printer and N_2 atmosphere	PA membrane	7
UiO-66-NH-C18	Modification with Octadecanoyl chloride	151.7	High	24 h/45 °C	Et_3N/DMF/THF	No	48.1	No	Melamine sponge	8
S-MIL-101	Modification with OA	156 ± 1	Moderate	24 h/120 °C	Toluene	No	95.8	N_2 atmosphere	-	9
S-UiO-66	Modification with OA	154 ± 1	Moderate	24 h/120 °C	Toluene	No	91.8	N_2 atmosphere	-	9
S-ZIF-67	Modification with OA	151 ± 1	Moderate	24 h/120 °C	Toluene	No	97.3	N_2 atmosphere	-	9

S-HKUST	Modification with OA	155 ± 1	Moderate	24 h/120 ºC	Toluene	No	98.7	N_2 atmosphere	-	9
OPA-UiO-66	Modification with OPA	160	High	24 h/room temp.	EtOH	No	~ 100	No	-	10
OPA-UiO-66-SO_3H	Modification with OPA	162	High	24 h/room temp.	EtOH	No	~ 100	No	-	10
OPA-PCN-222	Modification with OPA	157	High	24 h/room temp.	EtOH	No	95.3	No	-	10
Zr-UiO-67@F_9	Modification with 1H,1H,2H-Perfluoro-1-hexene	152	High	1. a) 1h/60 ºC b) 1 min/room temp. 2. a) 30 min/room temp. b) 3 h/Xe Lamp	1. DMF 2. 2-hydroxy-2-methyl propiophenone	Yes	14.6	Xe lamp	-	11
Zr-UiO-67@F_{17}	Modification with 1H,1H,2H-perfluoro-1-decene	155	High	1. a) 1h/60 ºC b) 1 min/room temp. 2. a) 30 min/room temp. b) 3 h/Xe Lamp	1. DMF 2. 2-hydroxy-2-methyl propiophenone	Yes	26.7	Xe lamp	-	11
SH ZIF-90	Modification with pentalfluorobenzylamine	152.4	High	24 h/100 ºC	MeOH	Yes	~ 0	No	-	12
MIL-53(Al)-AM_4	Modification with Valeric Anhydride	> 150	Moderate	24 h/80 ºC	CH_3CN	No	-	No	-	13
MIL-53(Al)-AM_6	Modification with Heptanoic Anhydride	> 150	Moderate	24 h/80 ºC	CH_3CN	No	-	No	-	13
NH_2-UiO-66(Zr)-shp	Modification with $PhSiH_3$	161	High	1. degas (Cs_2CO_3) 2. 24 h/80 ºC	2. CH_3CN Anhydrous	No	67.4	1. N_2 atmosphere 2. CO_2 atmosphere	Stainless mesh	14

UiO-66-NHCOR-3	Modification with isostearin chloride	158	Moderate	12 h/0 ºC	CH_2Cl_2	No	67.3	No	-	15
Cr-MIL-101-NHCOR	Modification with isostearin chloride	152	Moderate	12 h/0 ºC	CH_2Cl_2	No	-	No	-	15
NH_2-MIL-53(Al) microneedles on AAO	Modification with perfluorooctanoyl chloride	> 160	High	1. 48 h/120 ºC<br>2. 3 h/room temp.	1.H_2O/CH_3COOH<br>2. THF/Et_3N	Yes	-	Glove box	AAO membrane	16
MOF-199@FMON-3	Modification with tetrakis(4-ethynylphenyl)methane and 1,4-dibromotetrafluorobenzene	163	High	1. 30 min/room temp.<br>2. 24 h/90 ºC	1. Toluene/Et_3N<br>2. Toluene/Et_3N	Yes	38.8	No	-	17
Cu^{I}M@PS-2.5	Coating with Polystyrene	~ 160	Moderate	1. 5.5 h/room temp.<br>2. 6 h/200 ºC<br>3. a) 2.5 h/65 ºC<br>b) 10 min/65 ºC	1. n-hexane (anhydrous)<br>2. MeOH (anhydrous)<br>3. Styrene	No	46.5	No	-	18
ZIF-8@PDA-SF	Modification with 1H,1H,2H,2H-perfluorodecanethiol	149	High	1. 12 h/room temp.<br>2. 12 h/room temp.	1. MeOH<br>2. MeOH/Et_3N	Yes	77.7	O_2 atmosphere	-	19
ZIF-8@rGO	Modification with wrinkled rGO nanosheets	158	High	1. 8 h/room temp.<br>2. 165 °C	1. H_2O	No	-	Ultrasonic Spray	Polyurethane sponge	20
HFGO@ZIF-8	Modification with HFGO	162	High	1. 1 h/room temp.<br>2. 60 °C	1. $CHCl_3$<br>2. MeOH/$CHCl_3$	Yes	51.3	No	Polyurethane sponge	21
SIM-2(C_{12})/Al_2O_3	Modification with dodecylamine	> 150	Moderate	24 h/room temp.	MeOH (anhydrous)	No	23.8	No	AAO membrane	22,23

ZIF-7 array coatings	Hierarchical architecture/improved surface roughness	151.3	Moderate	1. 12 h/60 °C<br>2. 12 h/150 °C	1. MeOH<br>2. DMF	No	-	No	Copper mesh	[24]
ZIF-8-VF	Modification with 1H,1H,2H,2H-perfluorodecanethiol	173	High	10 h/60 °C	(trifluoromethyl)benzene	Yes	~ 100	N_2 atmosphere	-	[25]
MOF-5-VF	Modification with 1H,1H,2H,2H-perfluorodecanethiol	169	High	10 h/60 °C	(trifluoromethyl)benzene	Yes	~ 100	N_2 atmosphere	-	[25]
UiO-66-AM_4	Modification with Valeric Anhydride	150.7	Moderate	1. 30 min/room temp.<br>2. 30 min/60 °C	$CHCl_3$	No	77.2	No	-	[26]
ZrTz-68-C_{18}	Modification with 1-octadecene	152.1	Moderate	12 h/40 °C	$CHCl_3$	No	0.95	No	-	[26]
HKUST-1-HDA	Modification with hexadecylamine	152.8	Low	5 min/room temp.	-	No	1.8	No	-	[26]
CYCU-3-HDA	Modification with hexadecylamine	154.1	Low	5 min/room temp.	-	No	31.9	No	-	[26]
SH-NH_2-ZIF-7	Modification with stearic acid	156	Low	5 h/room temp.	EtOH	No	14	No	-	[27]
FG-ZIF-7	Modification with FG nanosheets	160 ± 1	High	3h/30-35 °C	EtOH/Toluene	Yes	-	No	Stainless Steel Mesh	[28]
FG-ZIF-11	Modification with FG nanosheets	160 ± 1	High	3h/30-35 °C	MeOH/Toluene	Yes	-	No	Cotton Fabric	[28]
UiO-66-Oleate-3/UiO-66-Oleate-5	***Oleate anion grafting***	***164/154***	***Very low***	***90 min/room. Temp.***	***H_2O***	***No***	***65.6/69.6***	***No***	***Cotton Fiber/Cotton Fabric***	***This work***
MOR-1-Oleate	***Oleate anion grafting***	***155***	***Very low***	***90 min/room. Temp.***	***H_2O***	***No***	***75.9***	***No***	-	***This work***

ZIF-8-Oleate	***Oleate anion grafting***	***154***	***Very low***	***90 min/room. Temp.***	H_2O	***No***	***~ 100***	***No***	***-***	***This work***
MIL-53(Al)-Oleate	***Oleate anion grafting***	***153***	***Very low***	***90 min/room. Temp.***	H_2O	***No***	***41.7***	***No***	***-***	***This work***
HKUST-1-Oleate-2	***Oleate anion grafting***	***166***	***low***	***90 min/room. Temp.***	***EtOH***	***No***	***92.1***	***No***	***Cotton Fabric***	***This work***

Special conditions: special synthetic conditions, use of specialized equipment etc.

Abbreviations

FAS-17: 1H,1H,1H,2H-Perfluorodecyltrimethoxysilane, **PDMS**: Polydimethylsiloxane, **PA**: Polyamide, **OA**: Octadecylamine, **OPA**: n-octadecylphosphonic acid, **$PhSiH_3$**: Phenylsilane , **AAO**: anodic aluminum oxide, **rGO**: reduced Graphene Oxide, **HFGO**: Highly Fluorinated Graphite Oxide, **FG**: Fluorinated Graphene

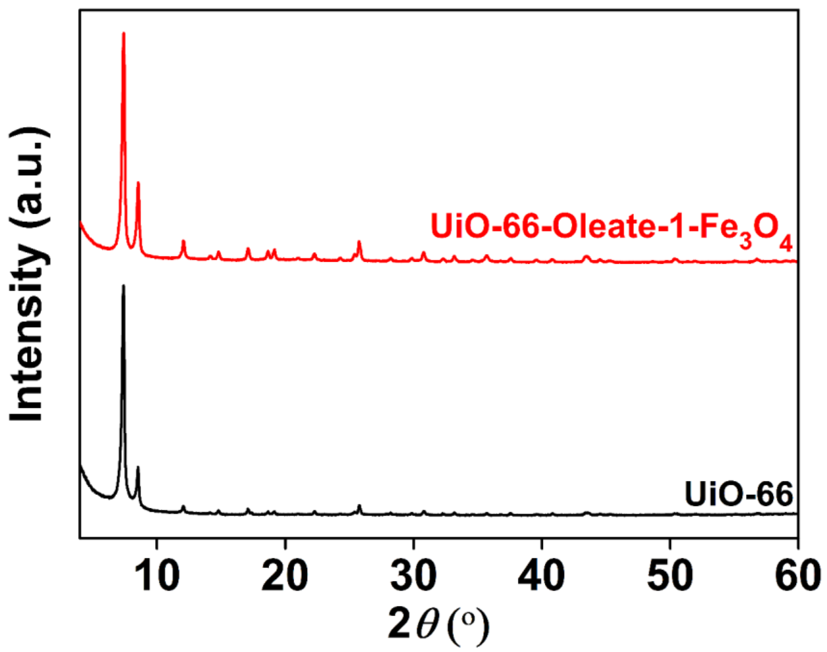

Figure S51. Comparative PXRD data for **UiO-66** and **UiO-66-Oleate-1-Fe_3O_4**.

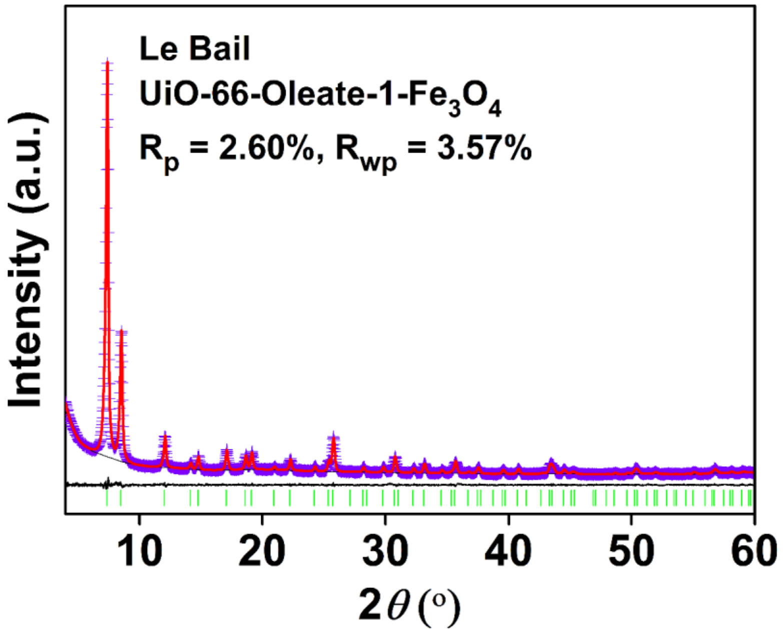

Figure S52. Le Bail plot of **UiO-66-Oleate-1-Fe_3O_4**. Violet crosses: experimental points; Red line: calculated pattern; Black line: difference pattern (exp. – calc.); Green bars: Bragg positions. Space group: *Fm-3m*. Cell parameters: a = 20.774(5) Å and V = 8966(7) $Å^3$.

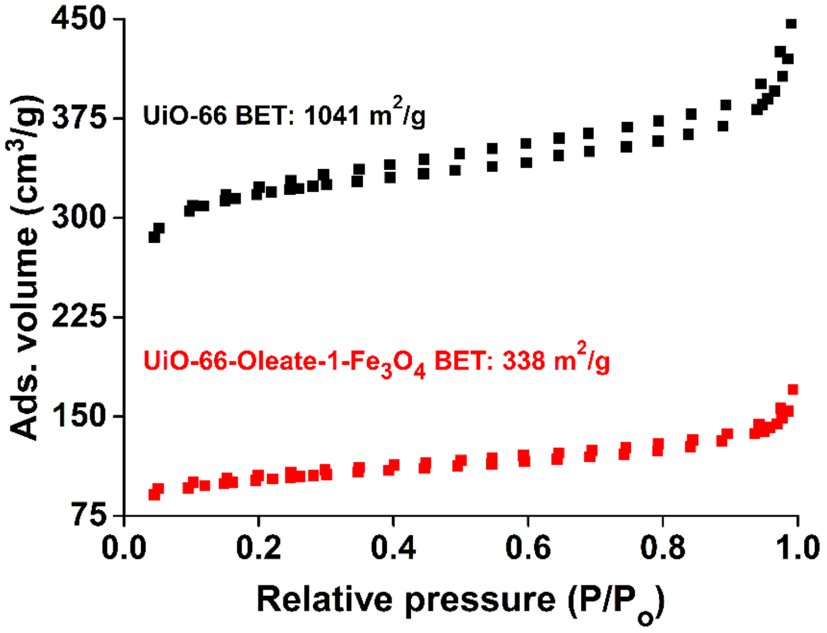

Figure S53. Nitrogen physisorption isotherms at 77 K for the activated **UiO-66** and **UiO-66-Oleate-1-Fe$_3$O$_4$**.

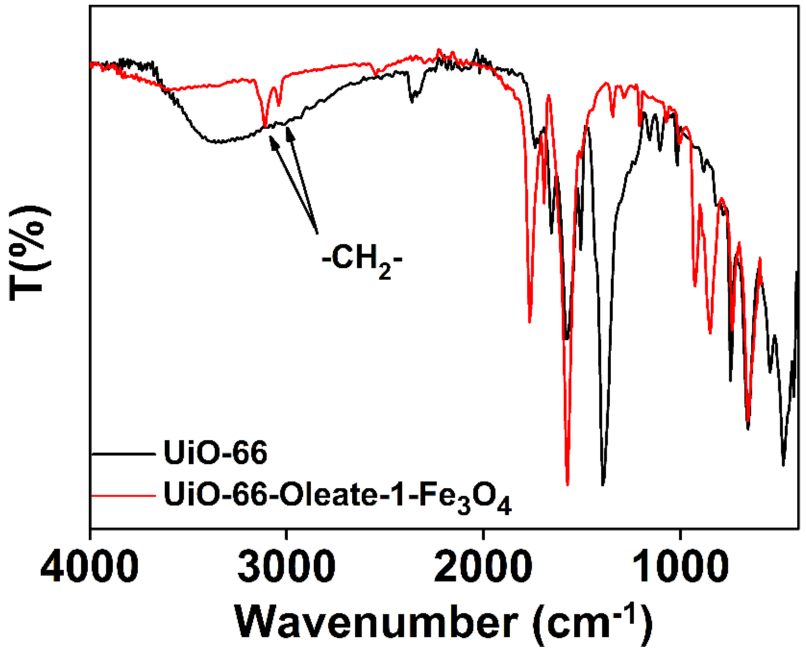

Figure S54. FTIR spectra of **UiO-66** and **UiO-66-Oleate-1-Fe$_3$O$_4$**.

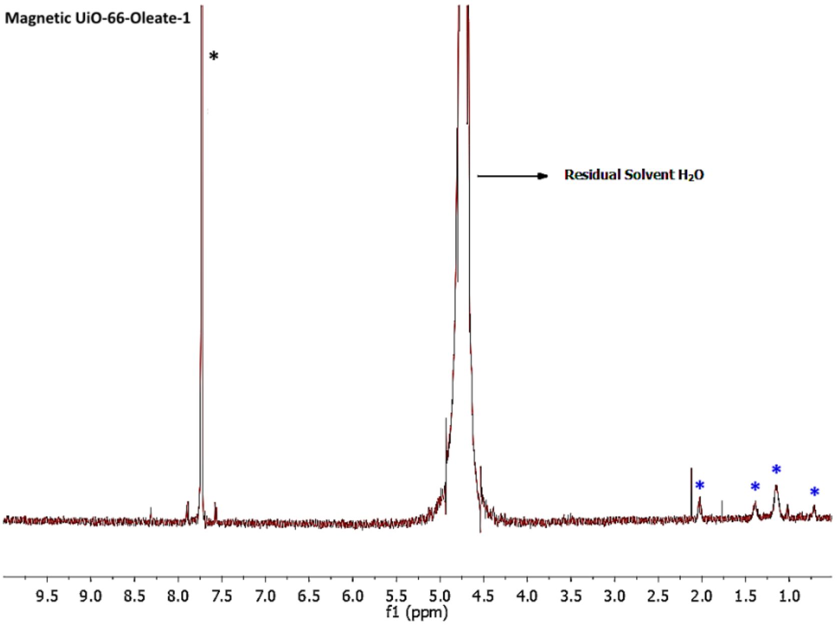

Figure S55. ^{1}H NMR spectrum of **UiO-66-Oleate-1-Fe_3O_4** after digestion in D_2O/NaOH solution. The peaks which are indicated with blue and black asterisks are assigned to oleate and terephthalate ions respectively. Based on the peak integrals the Oleate to MOF molar ratio was determined to be ~ 0.23.

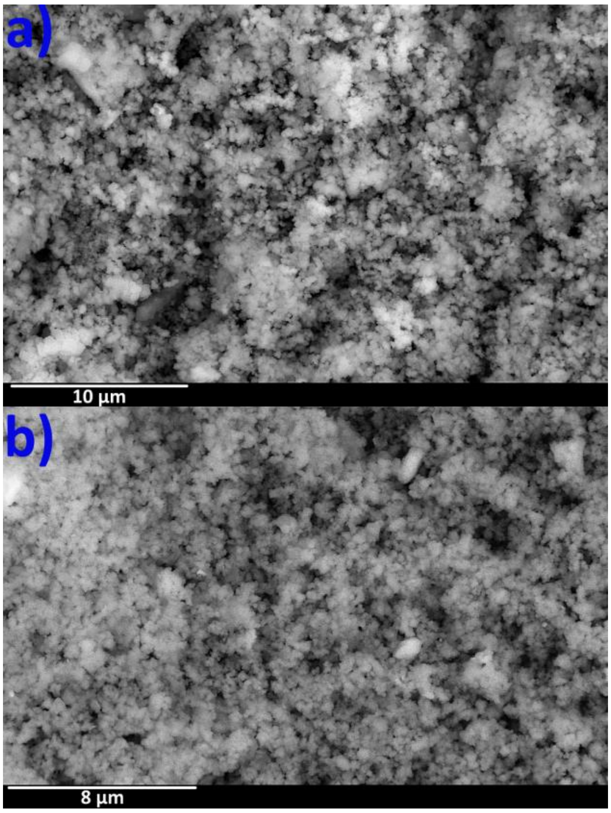

Figure S56. FE-SEM images of a) **UiO-66** and b) **UiO-66-Oleate-1-Fe_3O_4**.

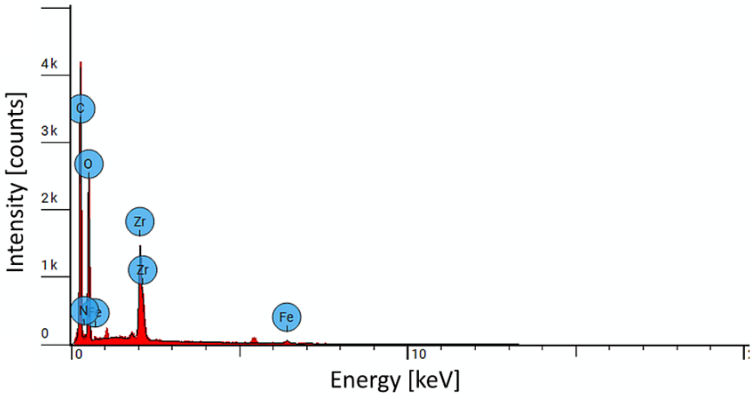

Figure S57. EDS spectra of **UiO-66-Oleate-1-Fe_3O_4**.

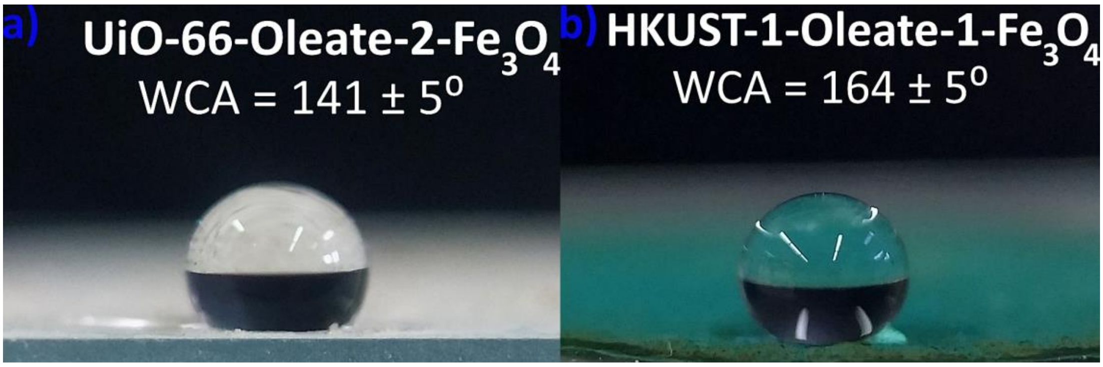

Figure S58. a) Digital images of a water droplets on thin films of a) **UiO-66-Oleate - 2-Fe_3O_4** and b) **HKUST-1-Oleate-1-Fe_3O_4**, along with the determined WCA values.

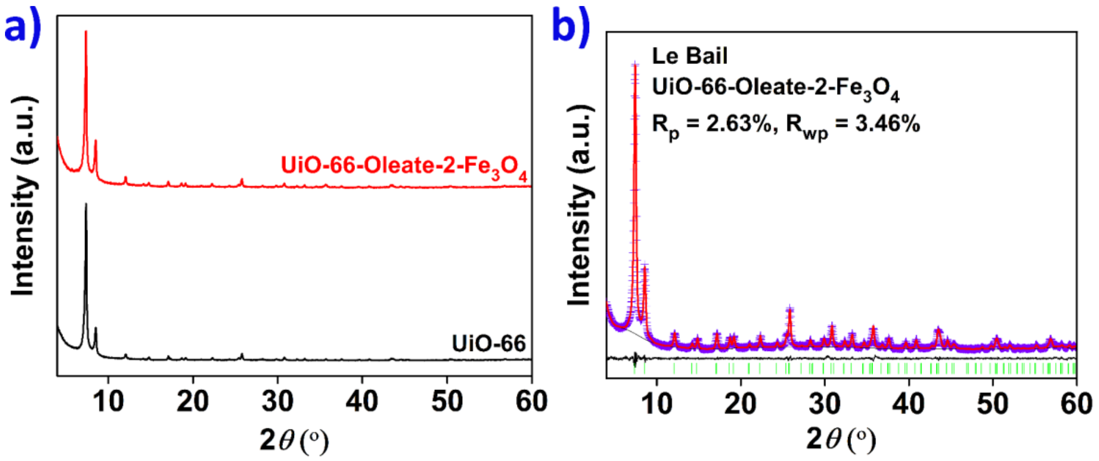

Figure S59. a) Comparative PXRD data for **UiO-66** and **UiO-66-Oleate-2-Fe_3O_4**. b) Le Bail plot of **UiO-66-Oleate-2-Fe_3O_4**. Violet crosses: experimental points; Red line: calculated pattern; Black line: difference pattern (exp. – calc.); Green bars: Bragg positions. Space group: *Fm-3m*. Cell parameters: a = 20.758(3) Å and V = 8945(6) Å^3.

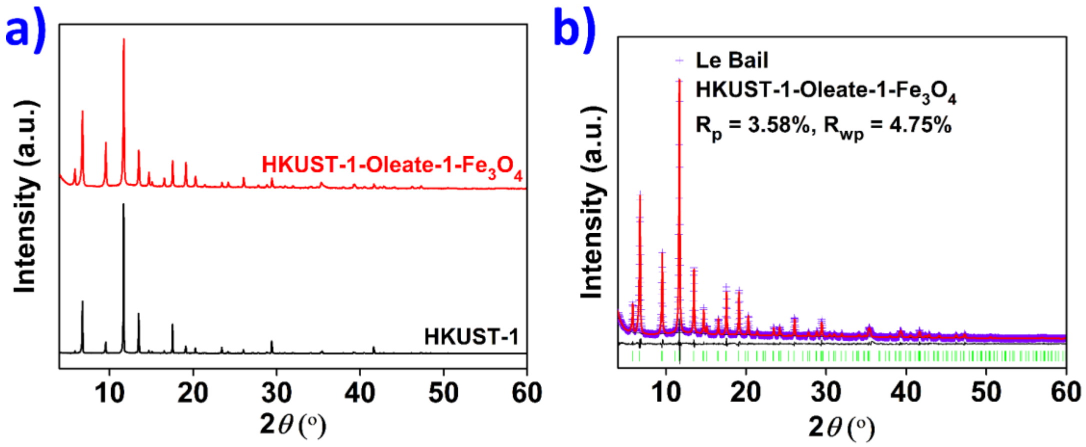

Figure S60. a) Comparative PXRD data for **HKUST-1** and **HKUST-1-Oleate-1-**$\mathbf{Fe_3O_4}$. b) Le Bail plot of **HKUST-1-Oleate-1-**$\mathbf{Fe_3O_4}$. Violet crosses: experimental points; Red line: calculated pattern; Black line: difference pattern (exp. – calc.); Green bars: Bragg positions. Space group: *Fm-3m*. Cell parameters: a = 26.315(2) Å and V = 18223(4) Å^3.

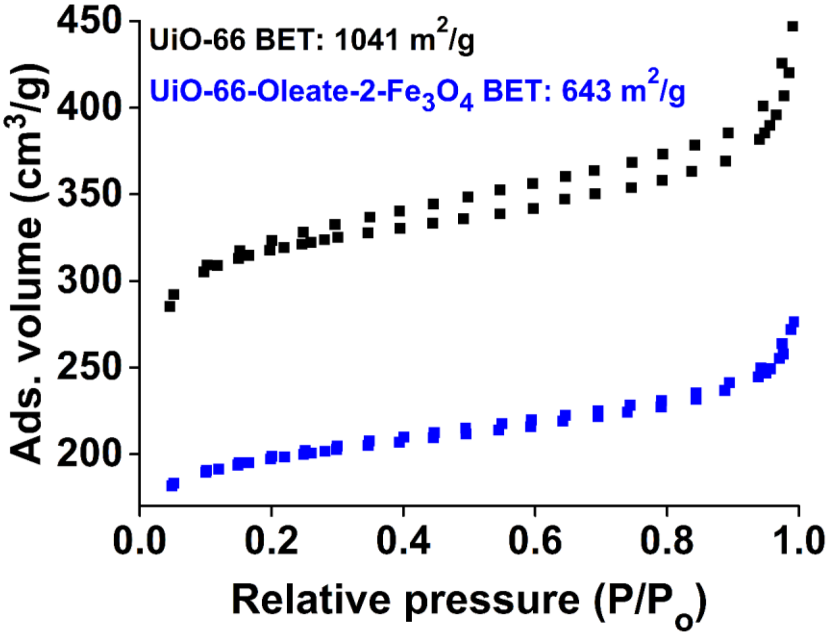

Figure S61. Nitrogen physisorption isotherms at 77 K for the activated **UiO-66** and **UiO-66-Oleate-2-**$\mathbf{Fe_3O_4}$.

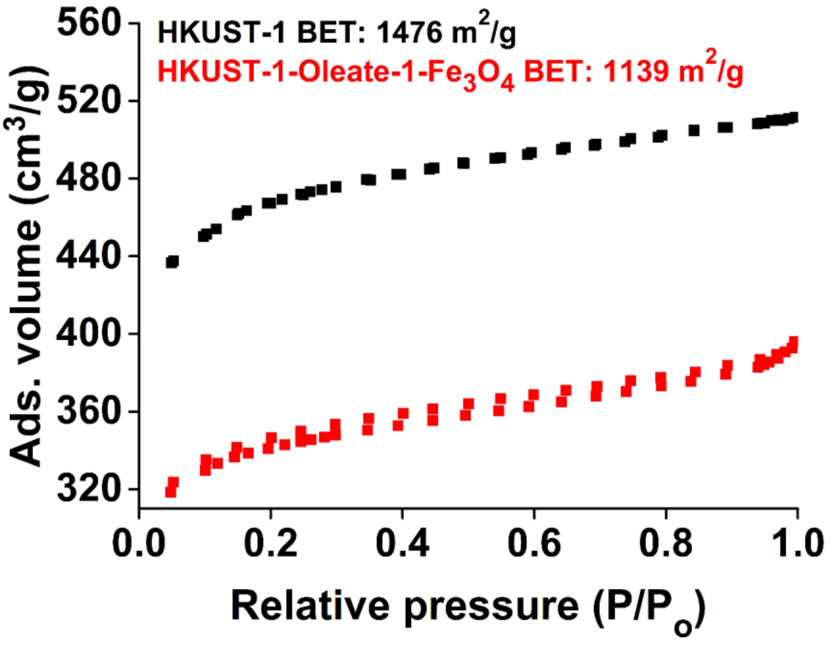

Figure S62. Nitrogen physisorption isotherms at 77 K for the activated **HKUST-1** and **HKUST-1-Oleate-1-Fe_3O_4**.

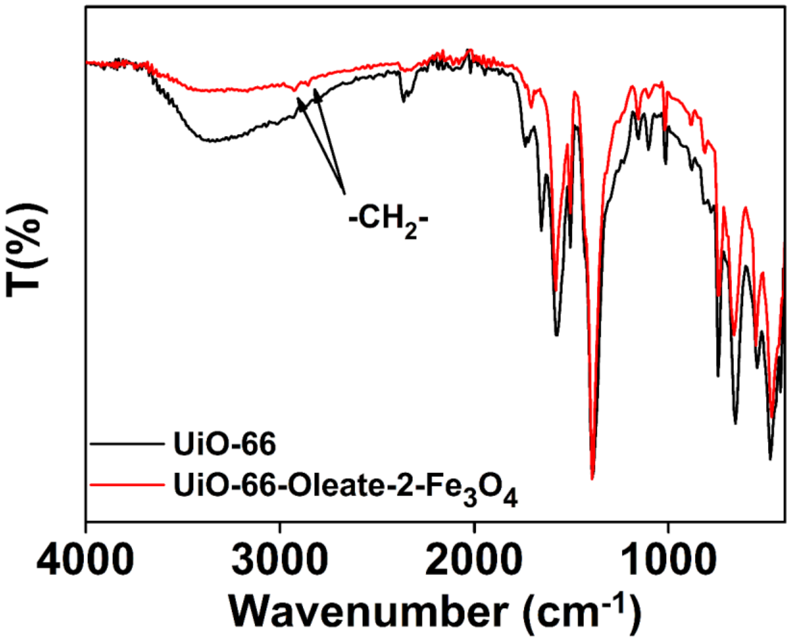

Figure S63. FTIR spectra of **UiO-66** and **UiO-66-Oleate-2-Fe_3O_4**.

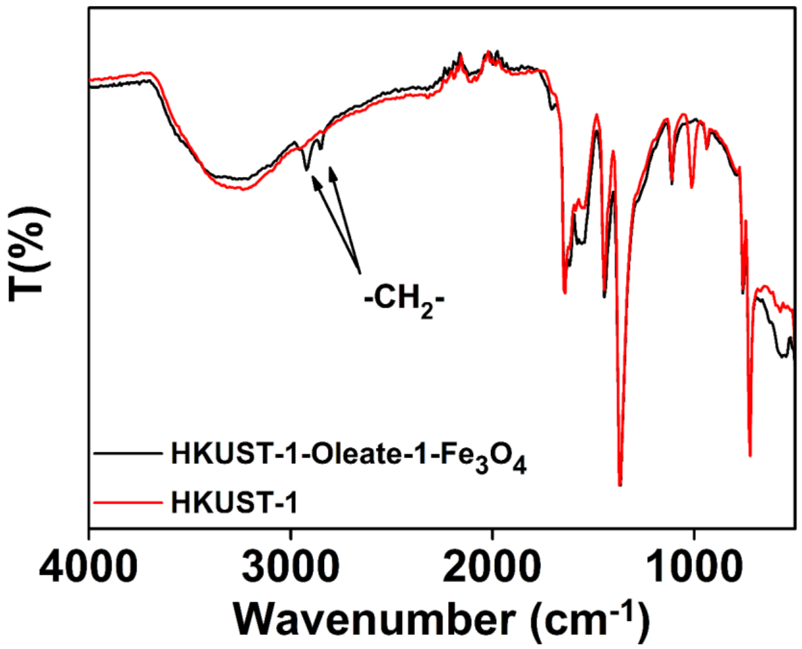

Figure S64. FTIR spectra of **HKUST-1** and **HKUST-1-Oleate-1-Fe_3O_4**.

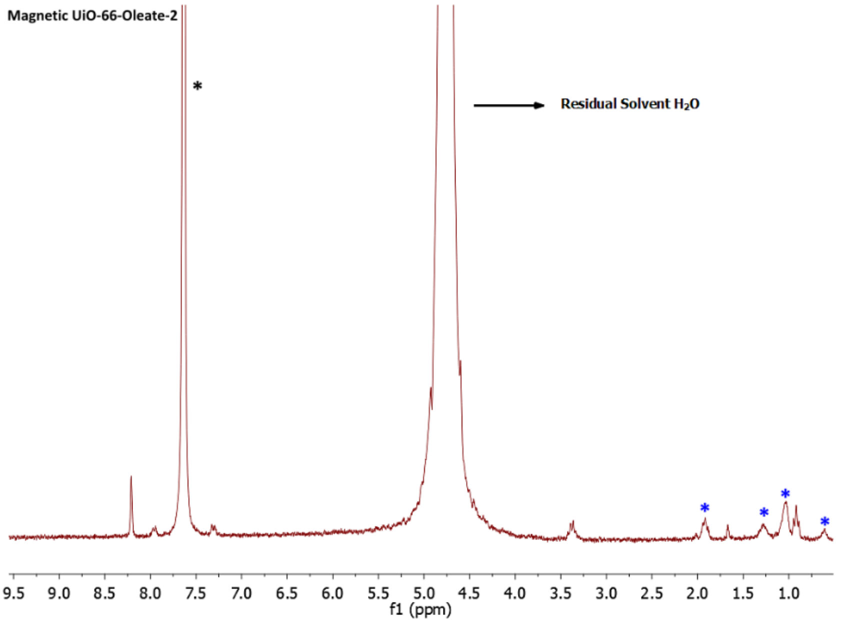

Figure S65. ^1H NMR spectrum of **UiO-66-Oleate-2-Fe_3O_4** after digestion in D_2O/NaOH solution. The peaks which are indicated with blue and black asterisks are assigned to oleate and terephthalate ions respectively. Based on the peak integrals the Oleate to MOF molar ratio was determined to be ~ 0.08. The sharp peak at 8.3 ppm is assigned to formic acid which is a base hydrolysis product of the residual DMF solvent.

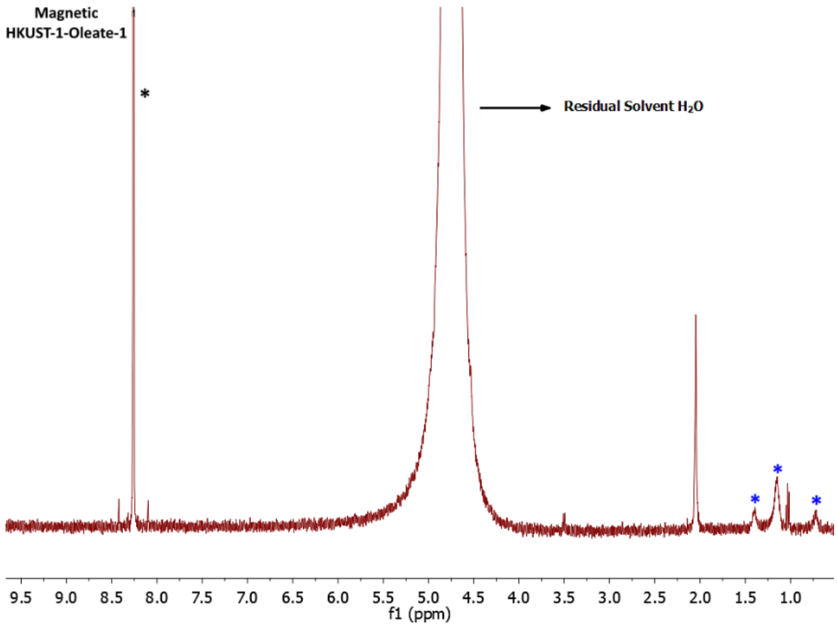

Figure S66. ^{1}H NMR spectrum of **HKUST-1-Oleate-1-Fe_3O_4** after digestion in D_2O/NaOH solution. The peaks which are indicated with blue and black asterisks are assigned to oleate and trimesate ions respectively. The first signal of oleate at 2.05 ppm is overlapped with signals from solvent impurities. Based on the peak integrals the Oleate to MOF molar ratio was determined to be ~ 0.09.

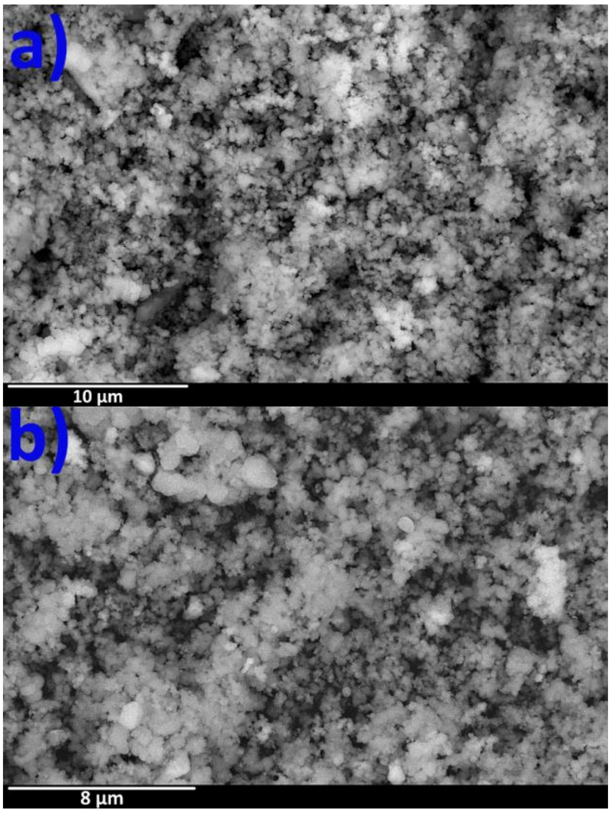

Figure S67. FE-SEM images of a) **UiO-66** and b) **UiO-66-Oleate-2-Fe_3O_4**.

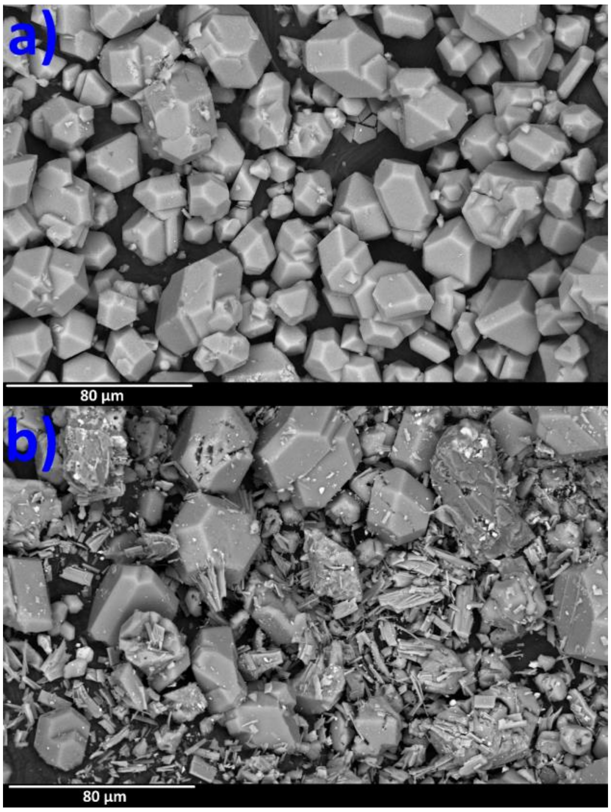

Figure S68. FE-SEM images of a) **HKUST-1** and b) **HKUST-1-Oleate-1-Fe_3O_4**.

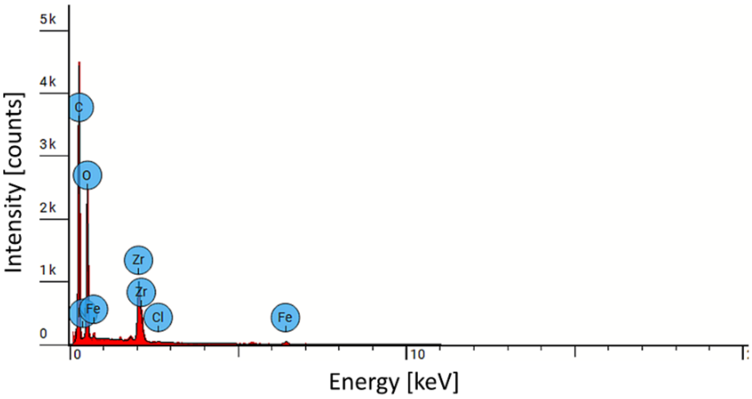

Figure S69. EDS spectrum of **UiO-66-Oleate-2-Fe_3O_4**.

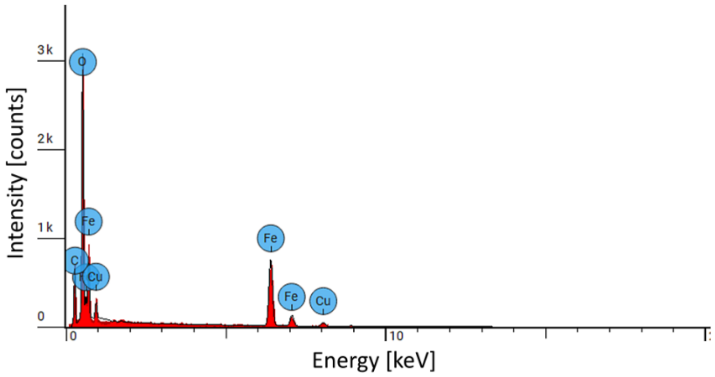

Figure S70. EDS spectrum of **HKUST-1-Oleate-1-Fe_3O_4**.

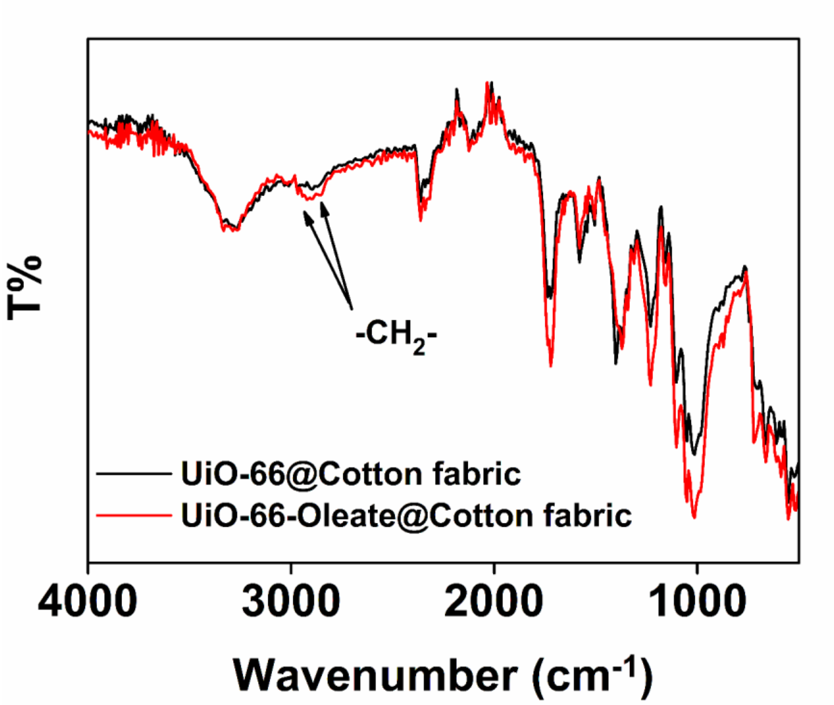

Figure S71. Comparative FTIR spectra of **UiO-66@Cotton fabric** and **UiO-66-Oleate@Cotton fabric**.

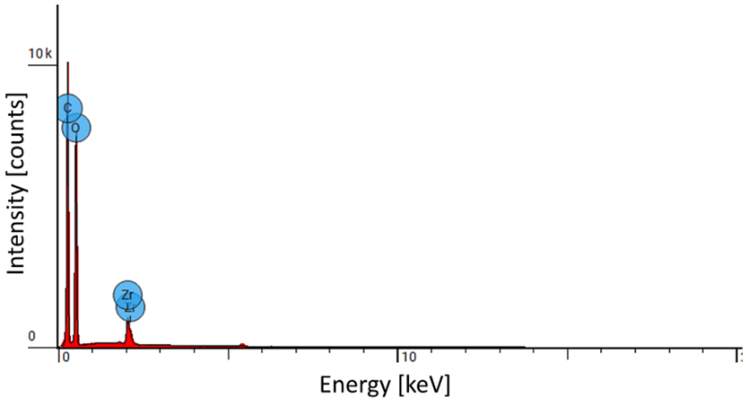

Figure S72. EDS spectrum of **UiO-66-Oleate@Cotton fabric**.

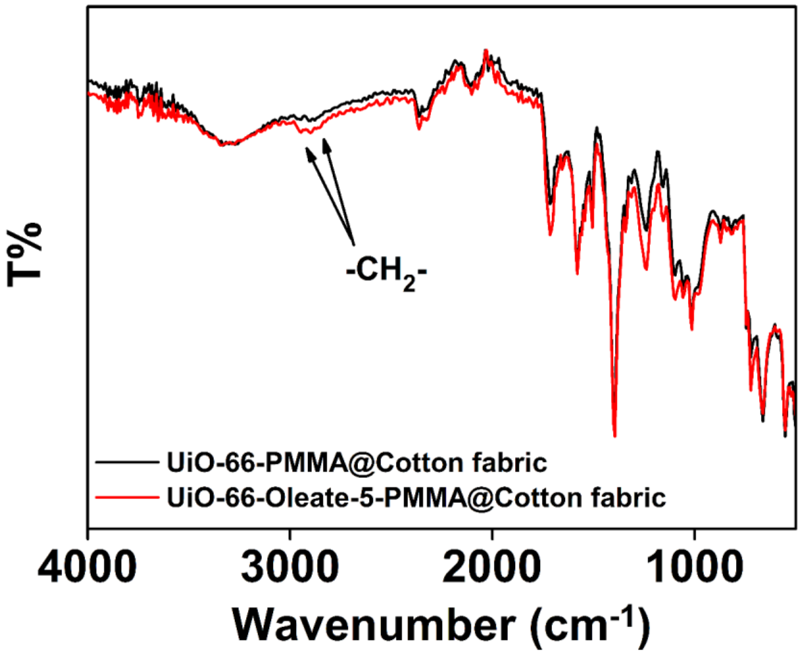

Figure S73. Comparative FTIR spectra of **UiO-66-PMMA@Cotton fabric** and **UiO-66-Oleate-5-PMMA@Cotton fabric**.

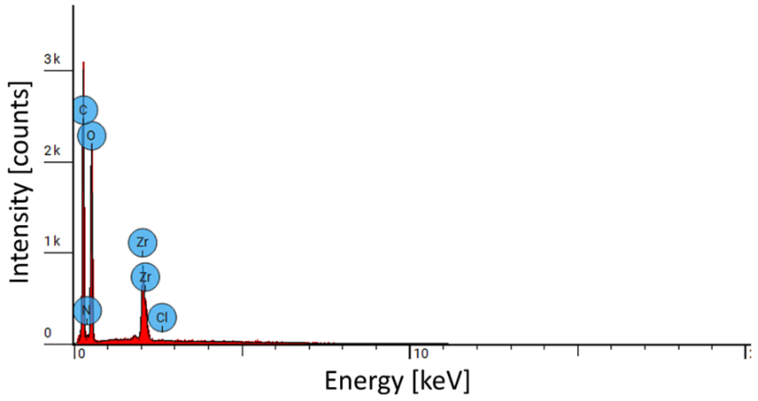

Figure S74. EDS spectrum of **UiO-66-Oleate-5-PMMA@Cotton fabric**.

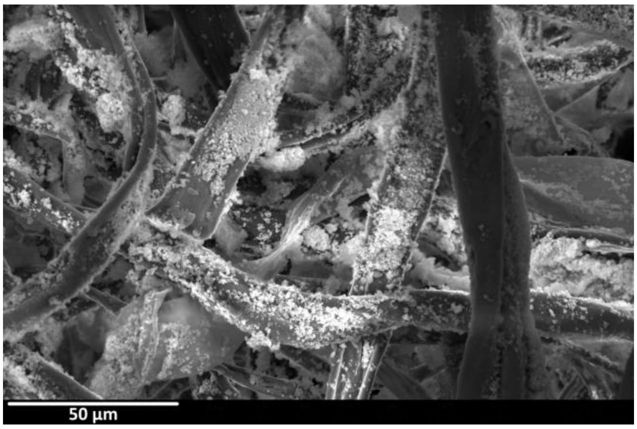

Figure 75. FE-SEM image of **UiO-66-Oleate-3-PMMA@Cotton**.

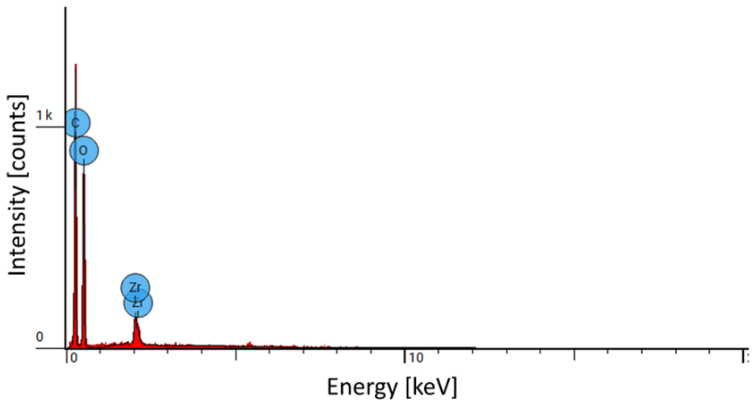

Figure S76. EDS spectrum of **UiO-66-Oleate-3-PMMA@Cotton**.

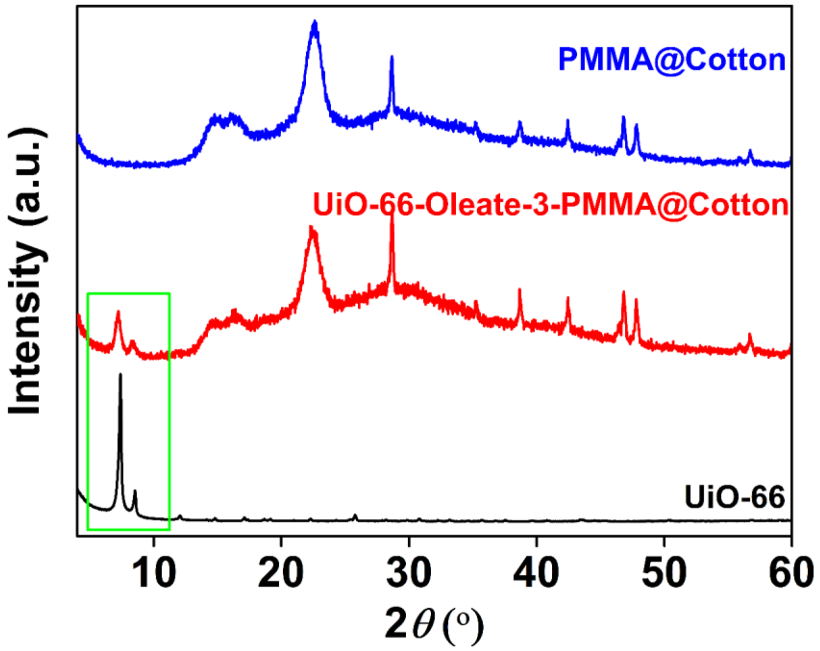

Figure S77. Comparative PXRD data of **UiO-66**, **UiO-66-Oleate-3-PMMA@Cotton** and **PMMA@Cotton**.

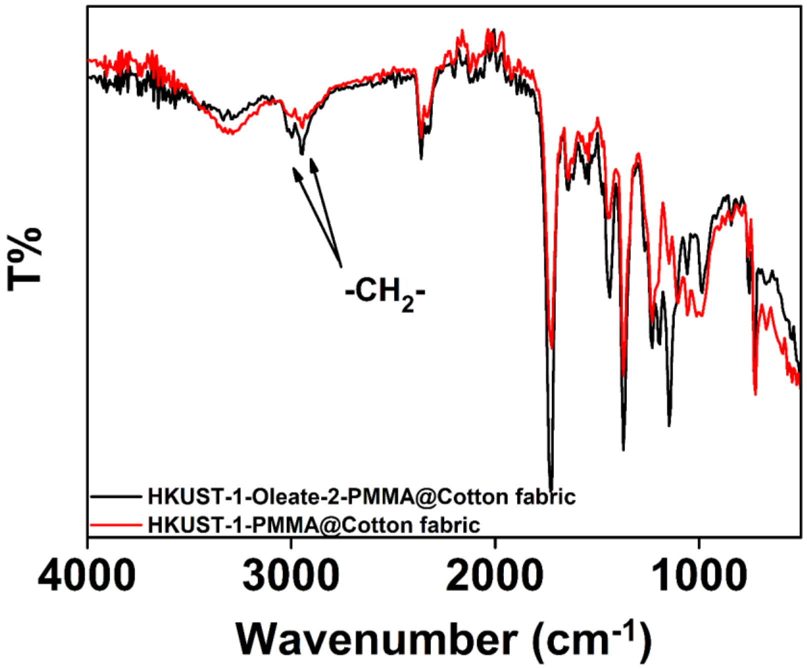

Figure S78. Comparative FTIR spectra of **HKUST-1-PMMA@Cotton fabric** and **HKUST-1-Oleate-2-PMMA@Cotton fabric**.

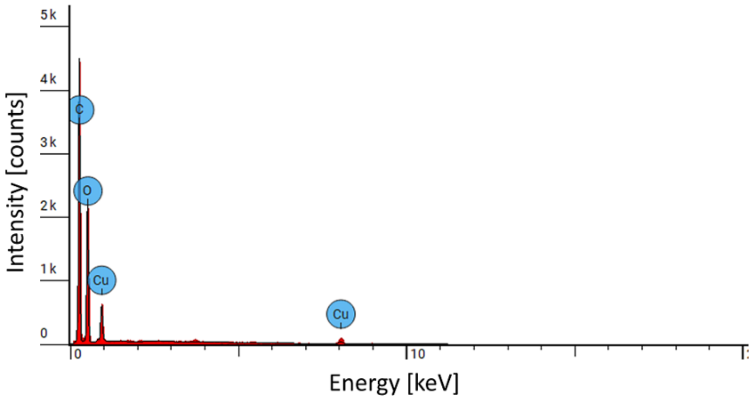

Figure S79. EDS spectrum of **HKUST-1-Oleate-2-PMMA@Cotton fabric**.

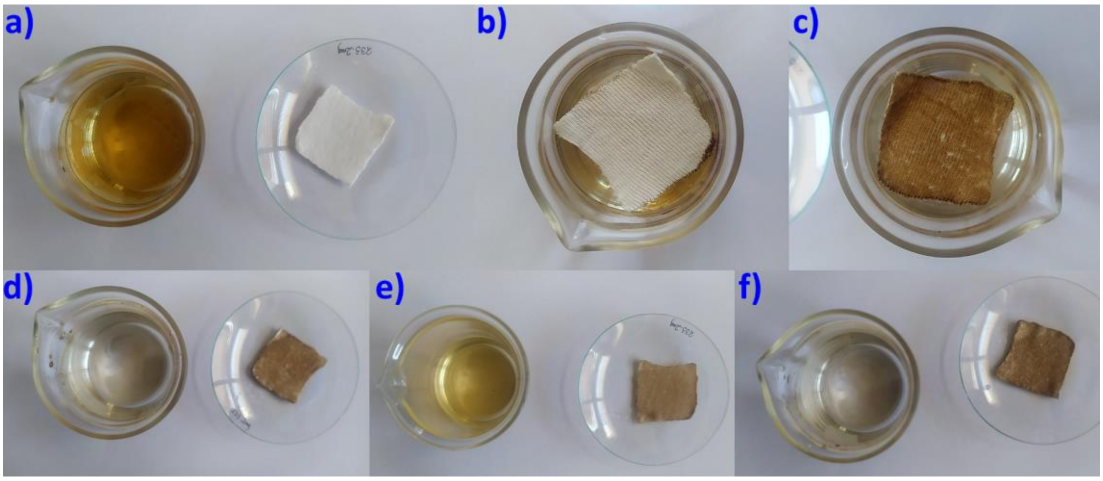

Figure S80. a-d) Crude oil sorption from the water surface by **UiO-66-Oleate@Cotton fabric**. e) Desorbed oil from the oil-laden fabric after its treatment with n-hexane and the regenerated **UiO-66-Oleate@Cotton fabric**. f) The oil-free water solution and the **UiO-66-Oleate@Cotton fabric** after the 10th cycle of sorption/regeneration.

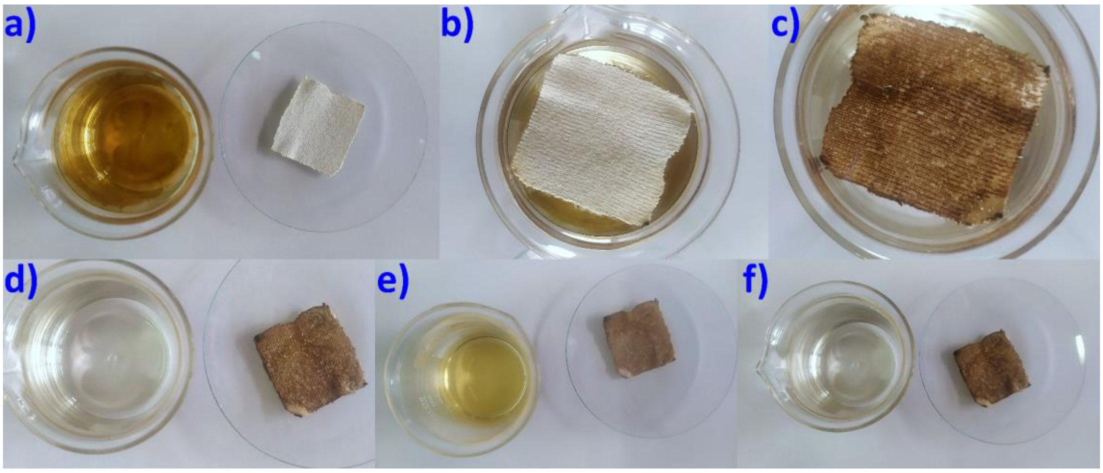

Figure S81. a-d) Crude oil sorption from the water surface by **UiO-66-Oleate-5-PMMA@Cotton fabric**. e) Desorbed oil from the oil-laden fabric after its treatment with n-hexane and the regenerated **UiO-66-Oleate-5-PMMA@Cotton fabric**. f) The oil-free water solution and the **UiO-66-Oleate-5-PMMA@Cotton fabric** after the 10th cycle of sorption/regeneration.

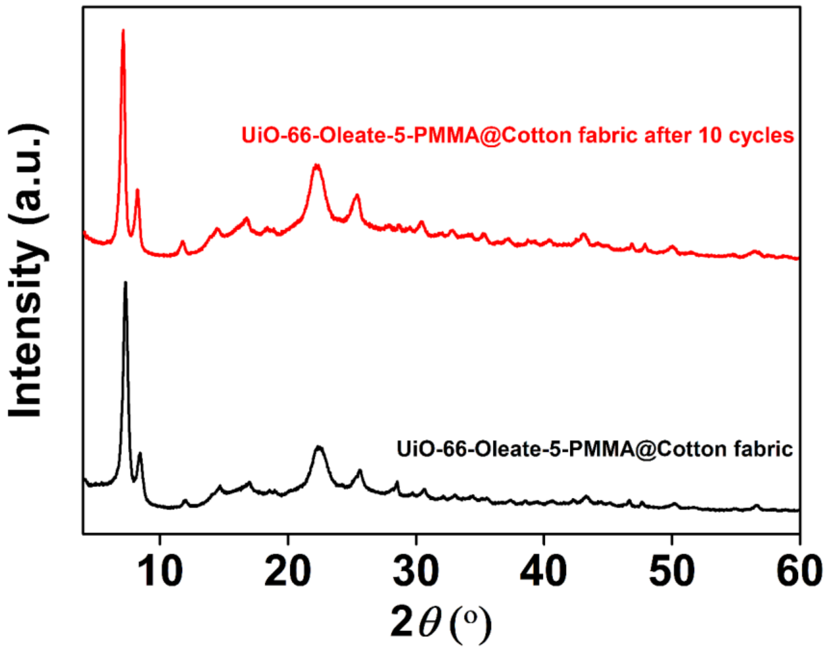

Figure S82. Comparative PXRD data of **UiO-66-Oleate-5-PMMA@Cotton fabric** before and after 10 sorption/regeneration cycles.

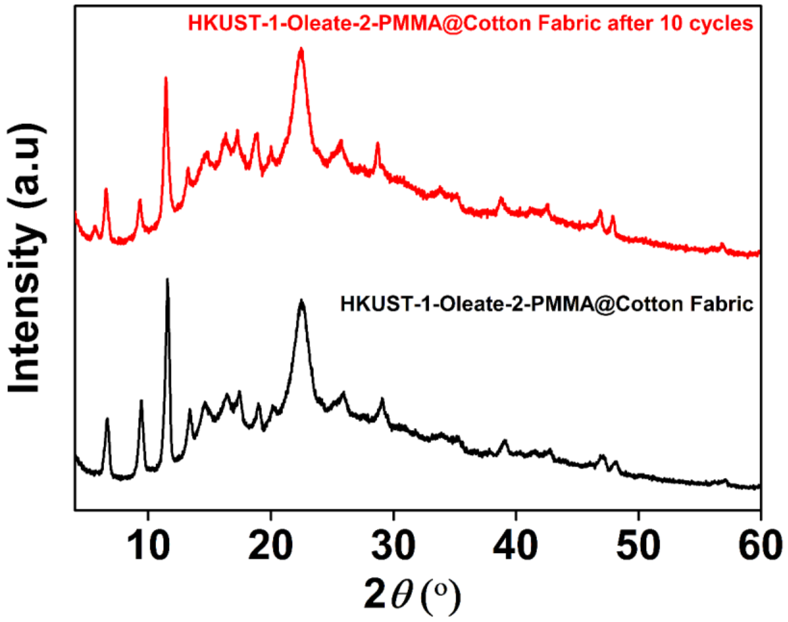

Figure S83. Comparative PXRD data of **HKUST-1-Oleate-2-PMMA@Cotton fabric** before and after 10 sorption/regeneration cycles.

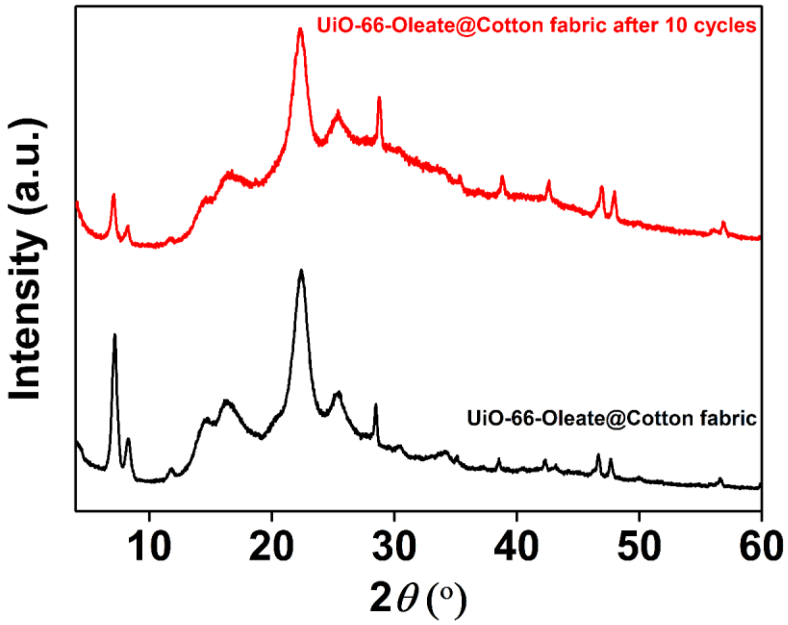

Figure S84. Comparative PXRD data of **UiO-66-Oleate@Cotton fabric** before and after 10 sorption/regeneration cycles.

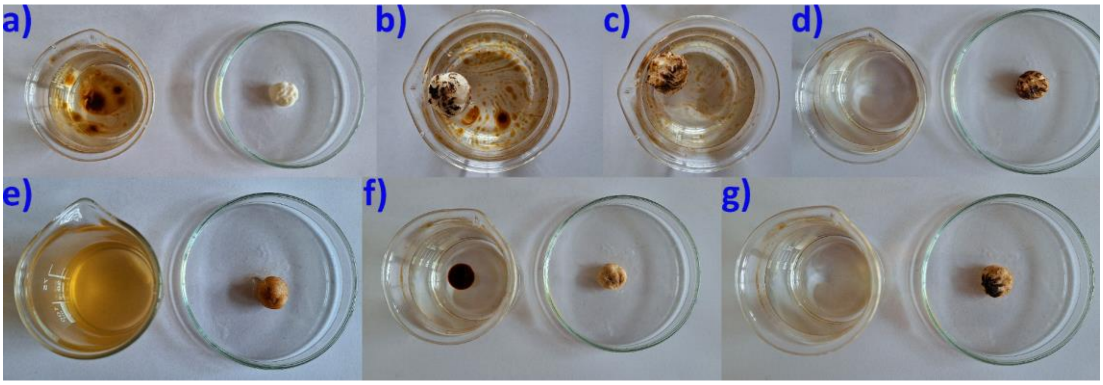

Figure S85. a-d) Crude oil sorption from the water surface by **UiO-66-Oleate-3-PMMA@Cotton**. e) Desorbed oil from the oil-laden cotton after its treatment with n-hexane and the regenerated **UiO-66-Oleate-3-PMMA@Cotton**. f and g) 2nd cycle of sorption.

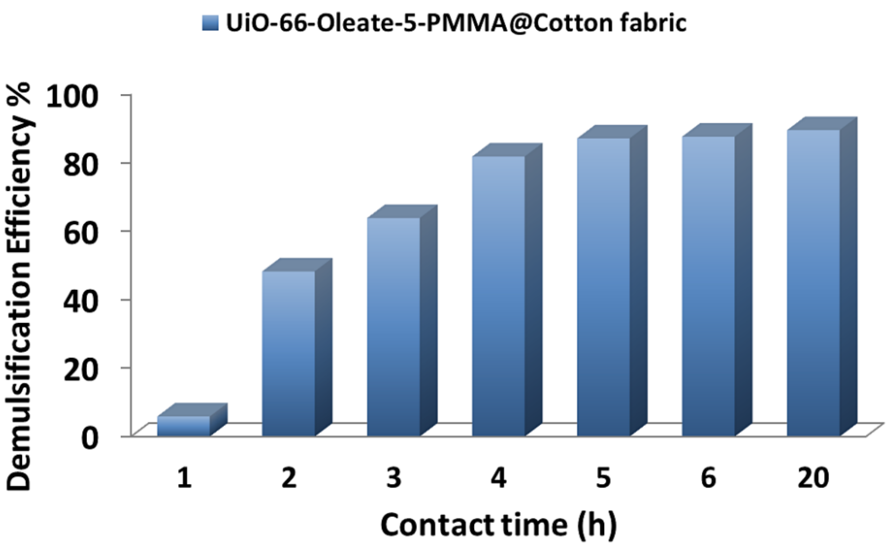

Figure S86. Sorption kinetics of vacuum pump oil-in-water separation for **UiO-66-Oleate-5-PMMA@Cotton fabric** (initial oil concentration ~ 540 ppm).

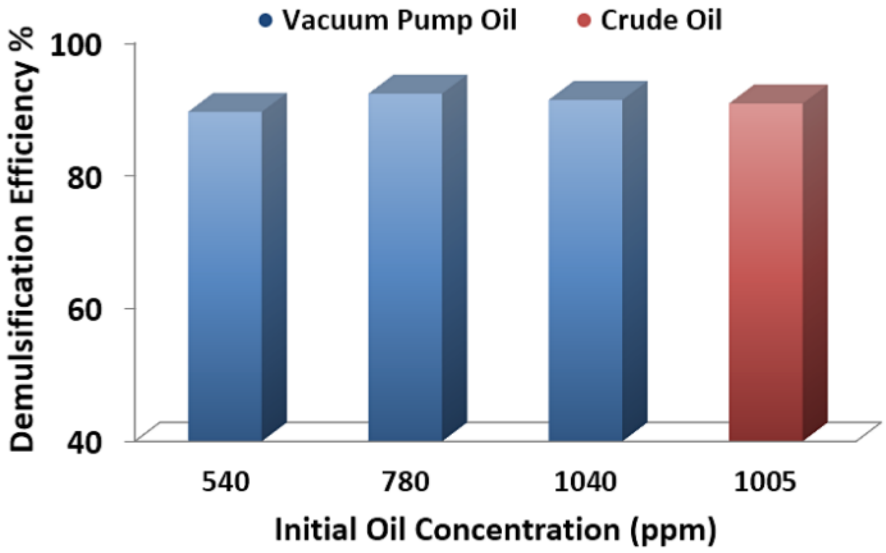

Figure S87. Vacuum pump and crude oil-in-water separation efficiency of **UiO-66-Oleate-5-PMMA@Cotton fabric** (contact time 20 h).

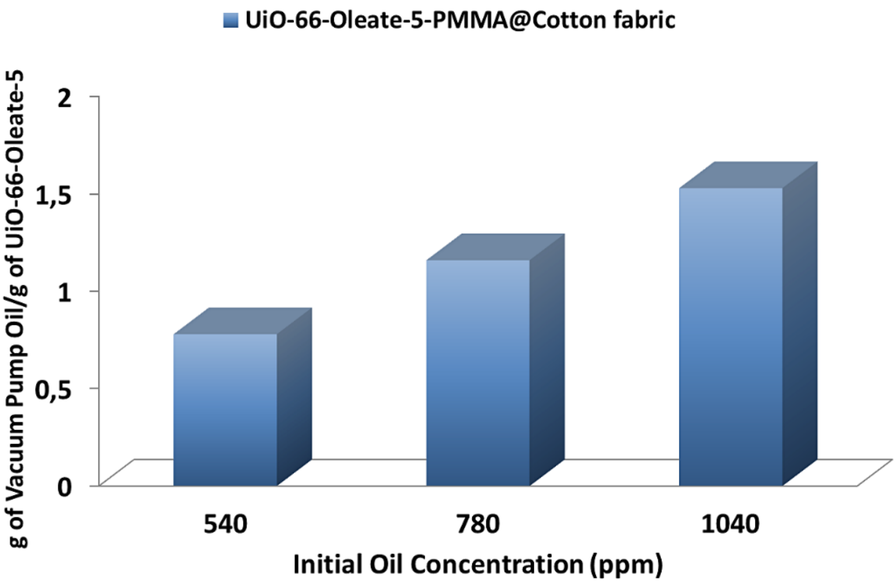

Figure S88. Determined sorption capacities of **UiO-66-Oleate-5-PMMA@Cotton fabric** for vacuum pump oil-in-water emulsions with varied oil concentrations (contact time 20 h).

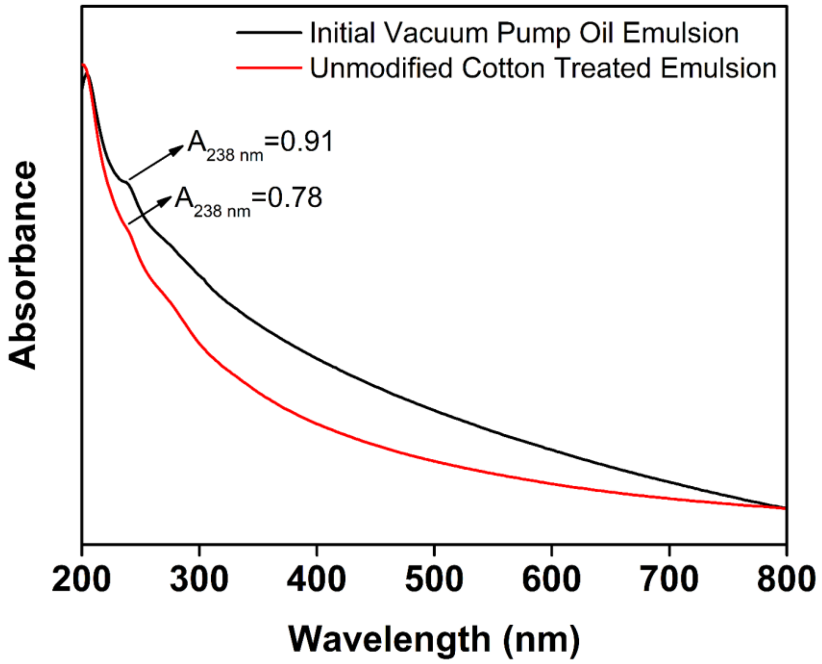

Figure S89. UV-vis spectra of the diluted vacuum pump oil emulsion (~ 500 ppm) before and after treatment with the unmodified cotton fabric.

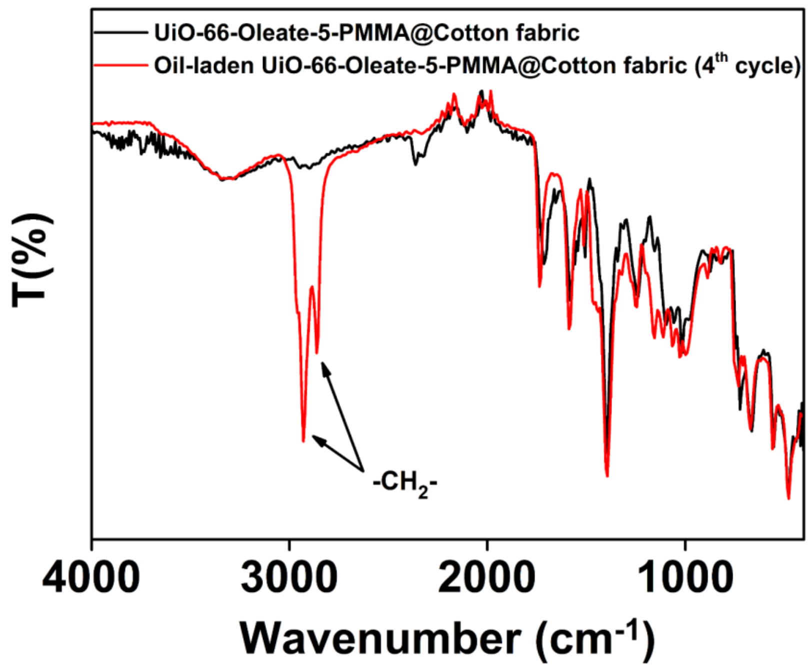

Figure S90. Comparative FTIR spectra of **UiO-66-Oleate-5-PMMA@Cotton fabric** and oil-laden **UiO-66-Oleate-5-PMMA@Cotton fabric** (4th cycle).

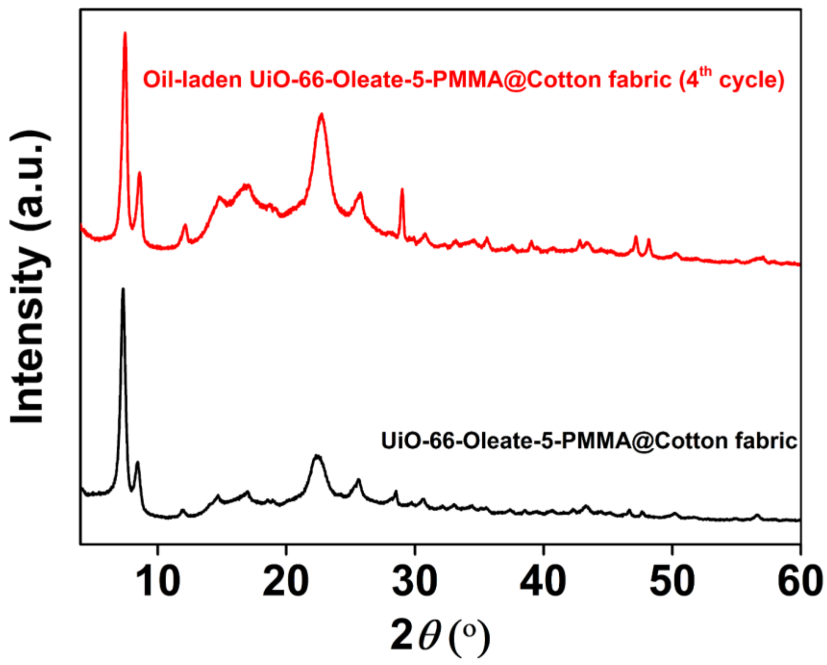

Figure S91. Comparative PXRD data of **UiO-66-Oleate-5-PMMA@Cotton fabric** and oil-laden **UiO-66-Oleate-5-PMMA@Cotton fabric** (4th cycle).

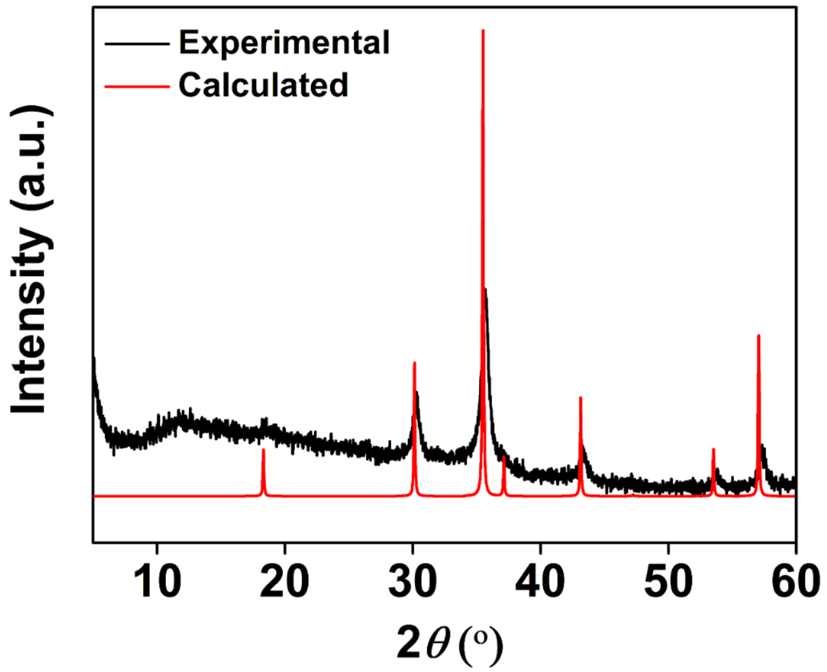

Figure S92. PXRD pattern of the as prepared and theoretical calculated Fe_3O_4.[29]

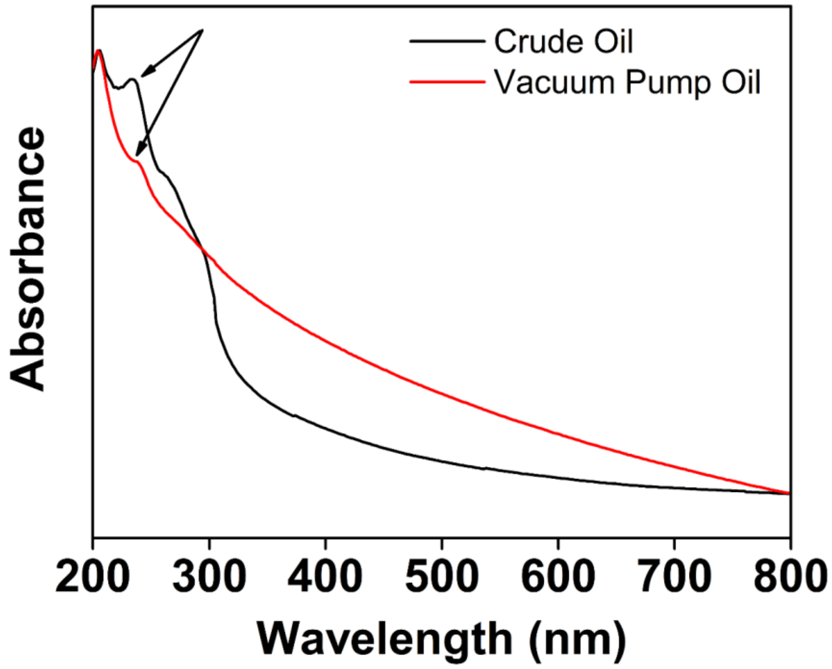

Figure S93. UV-vis spectra of vacuum pump oil and crude oil emulsions.

References

(1) Wagner, M.; Pigliapochi, R.; Di Tullio, V.; Catalano, J.; Zumbulyadis, N.; Centeno, S. A.; Wang, X.; Chen, K.; Hung, I.; Gan, Z.; Dworzak, M. R.; Yap, G.

P. A.; Dybowski, C. Multi-Technique Structural Analysis of Zinc Carboxylates (Soaps). *Dalton Trans.* **2023**, *52* (18), 6152–6165.

(2) Shearer, G. C.; Chavan, S.; Bordiga, S.; Svelle, S.; Olsbye, U.; Lillerud, K. P. Defect Engineering: Tuning the Porosity and Composition of the Metal-Organic Framework UiO-66 via Modulated Synthesis. *Chem. Mater.* **2016**, *28* (11), 3749–3761.

(3) Du, J.; Zhang, C.; Pu, H.; Li, Y.; Jin, S.; Tan, L.; Zhou, C.; Dong, L. HKUST-1 MOFs Decorated 3D Copper Foam with Superhydrophobicity/Superoleophilicity for Durable Oil/Water Separation. *Colloids Surf. A Physicochem. Eng. Asp.* **2019**, *573*, 222–229.

(4) Lei, Z.; Feng, J.; Yang, Y.; Shen, J.; Zhang, W.; Wang, C. An Efficient Polymer Coating for Highly Acid-Stable Zeolitic Imidazolate Frameworks Based Composite Sponges. *J. Hazard. Mater.* **2020**, *382*.

(5) Feng, L.; Lo, S. H.; Tan, K.; Li, B. H.; Yuan, S.; Lin, Y. F.; Lin, C. H.; Wang, S. L.; Lu, K. L.; Zhou, H. C. An Encapsulation-Rearrangement Strategy to Integrate Superhydrophobicity into Mesoporous Metal-Organic Frameworks. *Matter* **2020**, *2* (4), 988–999.

(6) Li, W.; Shi, J.; Zhao, Y.; Huo, Q.; Sun, Y.; Wu, Y.; Tian, Y.; Jiang, Z. Superhydrophobic Metal-Organic Framework Nanocoating Induced by Metal-Phenolic Networks for Oily Water Treatment. *ACS Sustain. Chem. Eng.* **2020**, *8* (4), 1831–1839.

(7) Yuan, S.; Zhu, J.; Li, Y.; Zhao, Y.; Li, J.; Van Puyvelde, P.; Van Der Bruggen, B. Structure Architecture of Micro/Nanoscale ZIF-L on a 3D Printed Membrane for a Superhydrophobic and Underwater Superoleophobic Surface. *J. Mater. Chem. A.* **2019**, *7* (6), 2723–2729.

(8) Shi, M.; Huang, R.; Qi, W.; Su, R.; He, Z. Synthesis of Superhydrophobic and High Stable Zr-MOFs for Oil-Water Separation. *Colloids Surf. A Physicochem. Eng. Asp.* **2020**, *602*.

(9) Gao, M. L.; Zhao, S. Y.; Chen, Z. Y.; Liu, L.; Han, Z. B. Superhydrophobic/Superoleophilic MOF Composites for Oil-Water Separation. *Inorg. Chem.* **2019**, *58* (4), 2261–2264.

(10) Sun, Y.; Sun, Q.; Huang, H.; Aguila, B.; Niu, Z.; Perman, J. A.; Ma, S. A Molecular-Level Superhydrophobic External Surface to Improve the Stability of Metal-Organic Frameworks. *J. Mater. Chem. A.* **2017**, *5* (35), 18770–18776.

(11) Du, J.; Chen, L.; Zhou, C.; Zhou, W.; Shen, H.; Zeng, X.; Zhou, P.; Tan, L.; Dong, L. Stable Zr-UiO-67 Constructed through Polymeric Network Assisted Post-Synthetic Modification and Its Wettability Modulation. *Chem. Commun.* **2021**, *57* (84), 11021–11024.

(12) Liu, C.; Liu, Q.; Huang, A. A Superhydrophobic Zeolitic Imidazolate Framework (ZIF-90) with High Steam Stability for Efficient Recovery of Bioalcohols. *Chem. Commun.* **2016**, *52* (16), 3400–3402.

(13) Nguyen, J. G.; Cohen, S. M. Moisture-Resistant and Superhydrophobic Metal-Organic Frameworks Obtained via Postsynthetic Modification. *J. Am. Chem. Soc.* **2010**, *132* (13), 4560–4561.

(14) Sun, D.; Adiyala, P. R.; Yim, S.; Kim, D. Pore-Surface Engineering by Decorating Metal-Oxo Nodes with Phenylsilane to Give Versatile Super-Hydrophobic Metal–Organic Frameworks (MOFs). *Angew. Chem. Int. Ed.* **2019**, *58* (22), 7405–7409.

(15) Zha, Q.; Sang, X.; Liu, D.; Wang, D.; Shi, G.; Ni, C. Modification of Hydrophilic Amine-Functionalized Metal-Organic Frameworks to Hydrophobic for Dye Adsorption. *J. Solid. State. Chem.* **2019**, *275*, 23–29.

(16) Tan, T. T. Y.; Reithofer, M. R.; Chen, E. Y.; Menon, A. G.; Hor, T. S. A.; Xu, J.; Chin, J. M. Tuning Omniphobicity via Morphological Control of Metal-Organic Framework Functionalized Surfaces. *J. Am. Chem. Soc.* **2013**, *135* (44), 16272–16275.

(17) Yu, H. P.; Bi, X. D.; He, Y. J.; Cui, Y. Y.; Yang, C. X. Microporous Organic Network: Superhydrophobic Coating to Protect Metal-Organic Frameworks from Hydrolytic Degradation. *ACS Appl. Mater. Interfaces.* **2023**, *15* (30), 36822–36830.

(18) Li, Y. X.; Ji, Y. N.; Mao, S. X.; Jin, M. M.; Liu, X. Q.; Sun, L. B. Construction of a Superhydrophobic Microenvironment: Via Polystyrene Coating: An Unexpected Way to Stabilize Cu^{I} against Oxidation. *Inorg. Chem. Front.* **2021**, *8* (24), 5169–5177.

(19) Yang, S.; Peng, L.; Sun, D. T.; Asgari, M.; Oveisi, E.; Trukhina, O.; Bulut, S.; Jamali, A.; Queen, W. L. A New Post-Synthetic Polymerization Strategy Makes Metal-Organic Frameworks More Stable. *Chem. Sci.* **2019**, *10* (17), 4542–4549.

(20) Gu, J.; Fan, H.; Li, C.; Caro, J.; Meng, H. Robust Superhydrophobic/Superoleophilic Wrinkled Microspherical MOF@rGO Composites for Efficient Oil–Water Separation. *Angew. Chem. Int. Ed.* **2019**, *58* (16), 5297–5301.

(21) Jayaramulu, K.; Datta, K. K. R.; Rösler, C.; Petr, M.; Otyepka, M.; Zboril, R.; Fischer, R. A. Biomimetic Superhydrophobic/Superoleophilic Highly Fluorinated Graphene Oxide and ZIF-8 Composites for Oil–Water Separation. *Angew. Chem. Int. Ed.* **2016**, *55* (3), 1178–1182.

(22) Aguado, S.; Canivet, J.; Farrusseng, D. Engineering Structured MOF at Nano and Macroscales for Catalysis and Separation. *J. Mater. Chem.* **2011**, *21* (21), 7582–7588.

(23) Canivet, J.; Aguado, S.; Daniel, C.; Farrusseng, D. Engineering the Environment of a Catalytic Metal-Organic Framework by Postsynthetic Hydrophobization. *ChemCatChem* **2011**, *3* (4), 675–678.

(24) Zhang, G.; Zhang, J.; Su, P.; Xu, Z.; Li, W.; Shen, C.; Meng, Q. Non-Activation MOF Arrays as a Coating Layer to Fabricate a Stable Superhydrophobic Micro/Nano Flower-like Architecture. *Chem. Commun.* **2017**, *53* (59), 8340–8343.

(25) Sun, Q.; He, H.; Gao, W. Y.; Aguila, B.; Wojtas, L.; Dai, Z.; Li, J.; Chen, Y. S.; Xiao, F. S.; Ma, S. Imparting Amphiphobicity on Single-Crystalline Porous Materials. *Nat. Commun.* **2016**, *7*, 13300.

(26) Li, B. H.; Wang, S. L.; Pal, S.; So, P. B.; Chen, G. Y.; Huang, W. J.; Hsu, Y. L.; Kuo, S. Y.; Yeh, J. M.; Lin, C. H. Versatile Reactions on Hydrophobic Functionalization of Metal-Organic Frameworks and Anticorrosion Application. *Microporous and Mesoporous Mater.* **2021**, *325*.

(27) Zhao, Y.; Xu, T.; Zhou, J. H.; Hu, J. M. Superhydrophobic Nanocontainers for Passive and Active Corrosion Protection. *Chem. Eng. J.* **2022**, *433*.

(28) Prasanthi, I.; Datta, K. K. R. Three in One: Superoleophilic, Chemically and Mechanically Resistant ZIF-7 and ZIF-11 Percolation Networks for Selective Permeation of Oils and Chlorinated Solvents. *Inorg. Chem.* **2023**, *62* (43), 17791–17803.

(29) Ferreira, F. F.; Granado, E.; Carvalho, W.; Kycia, S. W.; Bruno, D.; Droppa, R. X-Ray Powder Diffraction Beamline at D10B of LNLS: Application to the Ba_2FeReO_6 Double Perovskite. *J. Synchrotron. Radiat.* **2006**, *13* (1), 46–53.
